# Targeting regulation of ATP synthase 5 alpha/beta dimerization alleviates senescence

**DOI:** 10.18632/aging.203858

**Published:** 2022-01-30

**Authors:** Yun Haeng Lee, Doyoung Choi, Geonhee Jang, Ji Yun Park, Eun Seon Song, Haneur Lee, Myeong Uk Kuk, Junghyun Joo, Soon Kil Ahn, Youngjoo Byun, Joon Tae Park

**Affiliations:** 1 Division of Life Sciences, College of Life Sciences and Bioengineering, Incheon National University, Incheon 22012, Korea; 2 College of Pharmacy, Korea University, Sejong 30019, Republic of Korea

**Keywords:** senescence amelioration, KB1541, 14–3–3ζ, ATPase synthase 5, OXPHOS

## Abstract

Senescence is a distinct set of changes in the senescence-associated secretory phenotype (SASP) and leads to aging and age-related diseases. Here, we screened compounds that could ameliorate senescence and identified an oxazoloquinoline analog (KB1541) designed to inhibit IL-33 signaling pathway. To elucidate the mechanism of action of KB1541, the proteins binding to KB1541 were investigated, and an interaction between KB1541 and 14–3–3ζ protein was found. Specifically, KB1541 interacted with 14–3–3ζ protein and phosphorylated of 14–3–3ζ protein at serine 58 residue. This phosphorylation increased ATP synthase 5 alpha/beta dimerization, which in turn promoted ATP production through increased oxidative phosphorylation (OXPHOS) efficiency. Then, the increased OXPHOS efficiency induced the recovery of mitochondrial function, coupled with senescence alleviation. Taken together, our results demonstrate a mechanism by which senescence is regulated by ATP synthase 5 alpha/beta dimerization upon fine-tuning of KB1541-mediated 14–3–3ζ protein activity.

## INTRODUCTION

Somatic cells lose their ability to proliferate after a finite number of cell divisions, a phenomenon known as senescence [1]. The hallmarks of senescence include permanent cell cycle arrest, marked changes in the morphology of organelles, and a senescence-associated secretory phenotype (SASP) [2]. The most striking change during senescence is SASP, in which senescent cells secrete large amounts of inflammatory cytokines, immunomodulators and proteases [3]. Analysis of changes in the age-dependent gene expression profile of SASP revealed stage-specific SASP expression during senescence [4]. Initially, immunosuppressive cytokines (characterized by TGF-β1 and TGF-β3) are released, but eventually inflammatory cytokines (characterized by IL-1β, IL-6, IL-8 and IL-33) are released [4]. Senescent cells change the neighboring microenvironment by gradually altering SASP expression.

IL-33 belongs to a member of the IL-1 family that stimulates the generation of T helper-2 related cytokines [5]. IL-33 is expressed in several cell types, including fibroblasts, epithelial cells, endothelial cells, and dendritic cells [6]. Increased IL-33 expression is one of the most striking changes associated with senescence [7]. In the central nervous system, upregulation of IL-33 expression was observed with senescence [7]. The significance of IL-33 in senescence is emphasized by the observation that IL-33 expression at both mRNA and protein level increases in age-related macular degeneration [8]. This suggests a prominent role for IL-33 in the aging process and provides an a priori basis for therapeutic strategies to reduce IL-33 as a possible intervention in patients with aging and age-related diseases. In our previous study, 20 oxazoloquinoline analogs were synthesized as IL-33 inhibitors, and 2D-NMR studies showed that the synthesized analogs were bound to structurally important residues of IL-33 [9].

Mitochondria are double-membraned organelles and are found in eukaryotes [10]. The primary function of mitochondria is to produce ATP in order to meet the energy demands of the cell. To this end, the mitochondrial inner membrane (IM) consists of an electron transport system that transfers protons across the IM into inter-membrane spaces, thereby generating a mitochondrial membrane potential (MMP) [10]. Then, ATP synthase 5 in mitochondrial IM synthesizes ATP from ADP using the electrochemical gradient created by the difference in MMPs [11]. ATP synthase 5 consists of two functional domains, F_1_ and F_0_ [11]. F_1_ domain comprises of five different subunits (three α, three β, and one γ, δ and ε) and F_0_ domain contains several accessary subunits. During assembly of the F_1_ domain, ATP synthase 5 alpha and beta subunits form a peripheral stator, the alpha/beta heterodimer [12]. ATP synthase 5 beta subunit contains a catalytic site at the interface with the adjacent alpha subunit, and ATP synthesis occurs at the catalytic site converting ADP to ATP [12]. However, the mechanism leading to alpha/beta heterodimerization has not been elucidated.

In this study, using in-house compound library containing 20 oxazoloquinoline analogs designed to IL-33 inhibitors [9], we aimed to identify compounds capable of ameliorating senescence. As the role of IL-33 in senescence is not clearly elucidated, we performed a pull-down assay and identified an interaction between a novel IL-33 inhibitor and 14–3–3ζ protein. Here, we demonstrate that IL-33 inhibitor can induce senescence improvement by regulating ATP synthase 5 alpha/beta dimerization via phosphorylation of 14–3–3ζ protein.

## RESULTS

### Chemical screening for compounds that ameliorate senescence phenotypes

**Figure 1:**
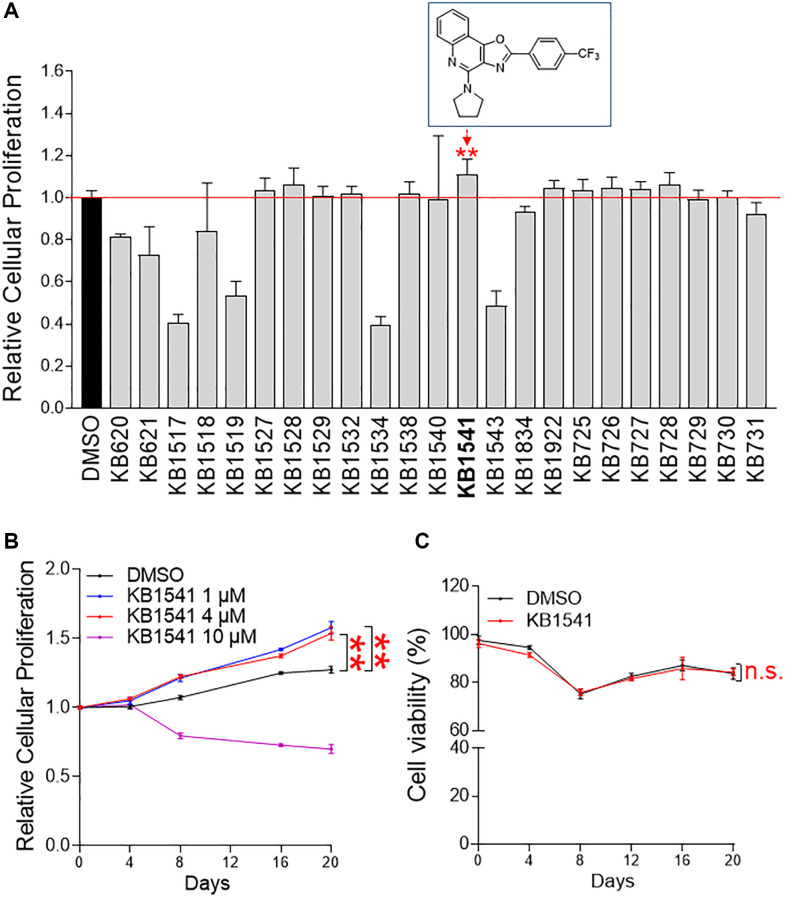
**KB1541 as a potential target for alleviating senescence.** (**A**) The level of cellular proliferation was measured quantitatively using a DNA content-based method. ^**^P < 0.01, Student's *t*-test. Mean ± S.D., *n* = 6. Chemical structure of an oxazoloquinoline derivative KB1541 was shown in rectangle. (**B**) Cell proliferation was assessed at different times (0–20 days) and concentrations of KB1541 (0–10 μM). ^**^P < 0.01, two-way ANOVA followed by Bonferroni’s post test. Mean ± S.D., *n* = 10. (**C**) The toxicity of KB1541 at 4 μM concentration was examined by measuring cell viability. n.s. (not significant), two-way ANOVA followed by Bonferroni’s post test. Mean ± S.D., *n* = 3.

In the present study, we employed a screening strategy measuring the candidate compounds capacity to increase cell number. For the screen, cell numbers were determined using a DNA content-based method [13]. An in-house compound library was treated on senescent fibroblasts and the effect on cell numbers was measured on day 12 (Figure 1A, Supplementary Tables 1 and 2). The compound leading to the maximum increase was considered a potential hit, thereby KB1541 was found as a candidate compound (rectangle in Figure 1A). KB1541 was an oxazoloquinoline derivative and was designed to inhibit IL-33 signal transduction [9] (rectangle in Figure 1A). The detailed reaction conditions and reagents to synthesize KB1541 are described in Supplementary Figure 1.

In order to verify the proliferation-inducing effect seen in screening, cell proliferation was assessed at different times (0–20 days) and concentrations (0–10 μM). The significant effects toward inducing cell proliferation were observed at 1 μM and 4 μM (concentrations used for screening) (Figure 1B). However, 10 μM concentration showed a cell proliferation inhibitory effect, indicating that concentrations exceeding the optimal concentration were toxic to cell proliferation (Figure 1B). Based on the proliferation results, the 4 μM concentration used for screening was selected for subsequent experiments. Next, the toxicity of KB1541 at the selected concentration was investigated by measuring cell viability. Senescent fibroblasts treated with KB1541 showed similar viability to those treated with DMSO, indicating that KB1541 treatment was not toxic to cells (Figure 1C). We then investigated whether KB1541 had an effect on cell proliferation even in young fibroblasts. Young fibroblasts were treated with 4 μM KB1541 at different times (0–20 days). No proliferation-inducing effect was observed in young fibroblasts treated with KB1541, indicating that the cell proliferation-inducing effect of KB1541 is limited to senescent fibroblasts (Supplementary Figure 2).

**Figure 2:**
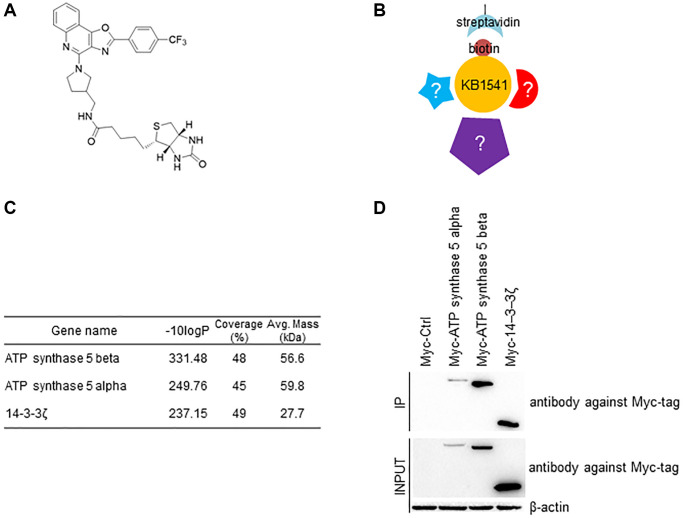
**Identification of KB1541 interacting proteins.** (**A**) Chemical structure of biotinylated KB1541. (**B**) Schematic diagram of immunoprecipitation workflow using biotinylated KB1541. (**C**) Ion Mobility Tandem Mass Spectrometer (IM-MS) identified 837 interacting proteins with biotinylated KB1541. Selected candidates - ATP synthase 5 alpha, ATP synthase 5 beta subunits and 14–3–3ζ protein are shown. -10 logP: -10 log (*p*-value), Coverage (%): a percentage of the total protein sequence covered by observed peptides, Avg. Mass (kDa): Average of protein molecular mass. (**D**) Co-precipitation of biotinylated KB1541 and Myc-tagged candidate proteins (Myc-tagged ATP synthase 5 alpha, Myc-tagged ATP synthase 5 beta subunits or Myc-tagged 14–3–3ζ proteins).

The role of IL-33 in senescence is not clearly elucidated, therefore discovery of a novel interacting partner will provide clues toward revealing its function. Hence, we biotinylated KB1541 for use as bait in pull-down assays (Figure 2A). Supplementary Figure 3 shows the detailed conditions for synthesizing biotinylated KB1541. During a pool-down assay, streptavidin precipitated the biotinylated KB1541, which led to co-precipitation of proteins that were binding to KB1541 (Figure 2B). We subsequently performed Ion Mobility Tandem Mass Spectrometer/Time-of-flight mass spectrometry (IM–MS/MS TOF) and identified 837 interacting proteins (Supplementary Table 3). Among the interacting proteins, a candidate-based approach, focusing on their level of affinity with KB1541 (indicated by *p*-value) and their association with senescence, was used. ATP synthase 5 alpha, ATP synthase 5 beta subunit and 14–3–3ζ protein were identified as most promising candidates, as the ATP synthesis mechanism provides ATP energy, which is essential for ameliorating senescence [14, 15] (Figure 2C). Specifically, ATP synthase 5 alpha and ATP synthase 5 beta subunits are components of ATP synthase 5 with the function of converting ADP to ATP [16], and 14–3–3ζ protein modifies the activity of ATP synthase 5 [17, 18]. To confirm the interaction between KB1541 and most promising candidates, pull-down assay was performed. HEK293T cells were transfected with pCMV-Myc-ATP synthase 5 alpha, pCMV-Myc-ATP synthase 5 beta, or pCMV-Myc-14–3–3ζ plasmids, and were cultured with media containing biotinylated KB1541. Consequently, cell lysates were precipitated with streptavidin and immunoblotted with an antibody against Myc-tag. Pull-down assay result showed that the biotinylated KB1541 interacted with Myc-tagged ATP synthase 5 alpha, Myc-tagged ATP synthase 5 beta, or Myc-tagged 14–3–3ζ proteins, confirming the IM–MS/MS TOF result (Figure 2D).

**Figure 3:**
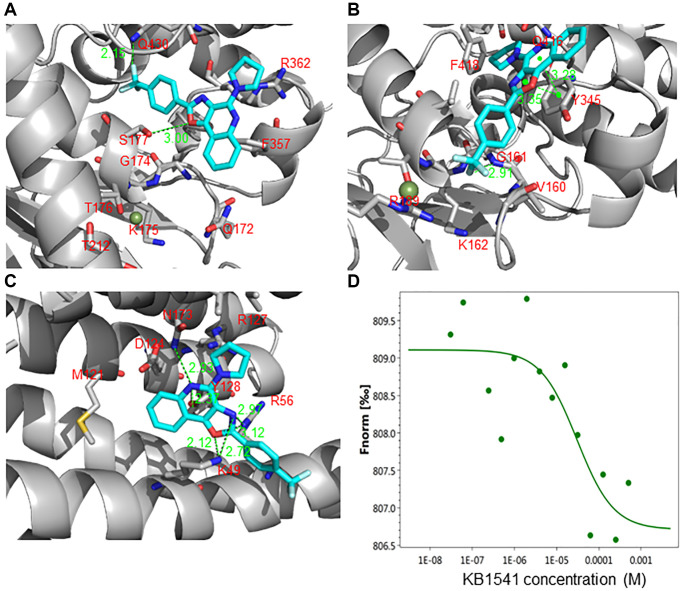
**KB1541 interacts with 14–3–3ζ protein.** (**A**) Binding mode of the KB1541 in ATP synthase 5 alpha (PDB ID: 2JDI). Dotted lines indicate hydrogen bonding interactions. Docking studies exhibited the possible hydrogen bonding interactions of KB1541 with S177 and Q430 in ATP synthase 5 alpha at 3.00 and 2.15 Å, respectively. (**B**) Binding mode of the KB1541 in ATP synthase 5 beta (PDB ID: 2JDI). Dotted lines indicate hydrogen bonding interactions. Docking studies exhibited three possible hydrogen bonding interactions of KB1541 with ATP synthase 5 beta (2.91, 3.28 and 3.35 Å). (**C**) Binding mode of the KB1541 in 14–3–3ζ. (PDB ID: 6FN9). Dotted lines indicate hydrogen bonding interactions. Docking studies of KB1541 with 14–3–3ζ showed that the best-docked pose was surrounded by K49, R56 and Y128. KB1541 was predicted to make strong hydrogen bonds with 14–3–3ζ using the central oxazoloquinoline ring (2.12, 2.97, 3.12 and 2.37 Å). (**D**) Microscale thermophoresis (MST) assay to quantify the binding between KB1541 and 14–3–3ζ protein. Data obtained was plotted with concentration on X-axis and Fnorm on Y-axis to find out the dissociation constant (*K*_d_). Fnorm value is calculated by dividing F_1_ by F_0_. F_1_ is the fluorescence value measured in the heated state, and F_0_ corresponds to the fluorescence value measured in the cold state before turning on the IR laser. *K*_d_ was found to be 29.8 μM, confirming a tight interaction between KB1541 and 14–3–3ζ protein.

We then performed an *in silico* docking study to predict the protein that most closely binds to KB1541 among the candidates. The potential binding modes of KB1541 with ATP synthase 5 alpha and ATP synthase 5 beta are shown in Figure 3A and 3B, respectively. Docking studies exhibited the possible hydrogen bonding interactions of KB1541 with S177 and Q430 in ATP synthase 5 alpha (PDB ID: 2JDI) at 3.00 and 2.15 Å, respectively (Figure 3A). Moreover, KB1541 was predicted to exhibit three hydrogen bonding interactions (2.91, 3.28 and 3.35 Å) with ATP synthase 5 beta (PDB ID: 2JDI) (Figure 3B). We then provided the potential binding mode of KB1541 with 14–3–3ζ. Docking studies of KB1541 with 14–3–3ζ (PDB ID: 6FN9) showed that the best-docked pose was surrounded by K49, R56 and Y128 (Figure 3C). KB1541 was predicted to make strong hydrogen bonds with 14–3–3ζ using the central oxazoloquinoline ring (2.12, 2.97, 3.12 and 2.37 Å) (Figure 3C). These data suggest that KB1541 might bind more tightly to 14–3–3ζ protein than ATP synthase 5 alpha and ATP synthase 5 beta. Thus, we focused on the 14–3–3ζ protein to elucidate the mechanisms that ameliorate senescence phenotypes by KB1541.

We then performed microscale thermophoresis (MST) assay to quantify the thermodynamics of KB1541 and 14–3–3ζ protein interaction. His-tagged 14–3–3ζ protein was purified, labeled and incubated with varying concentration of KB1541. The dissociation constant (*K*_d_) was found to be 29.8 μM, confirming a tight interaction between KB1541 and 14–3–3ζ protein (Figure 3D).

### 14–3–3ζ regulates ATP synthase 5 alpha/beta dimerization

**Figure 4:**
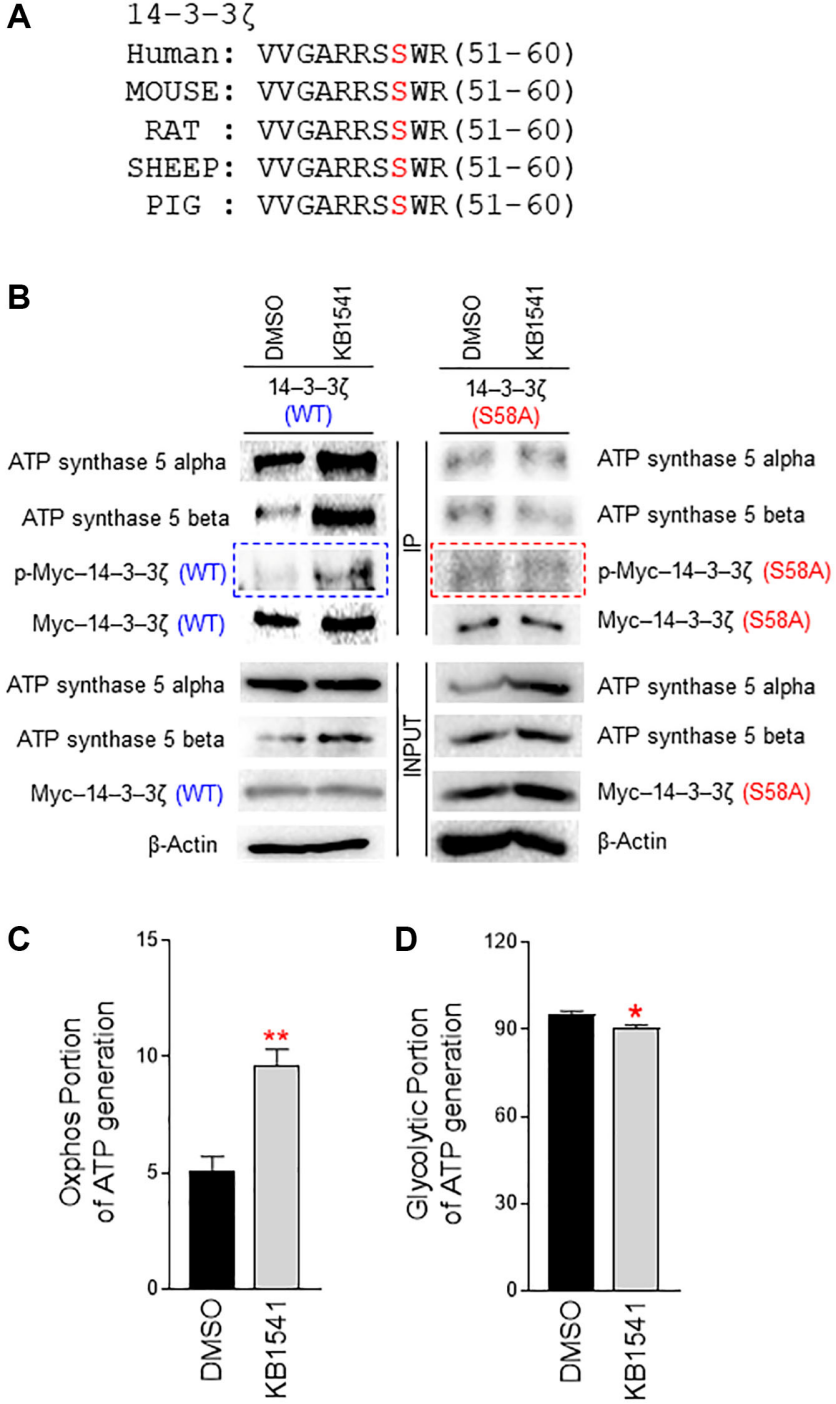
**14–3–3ζ regulates ATP synthase 5 alpha/beta dimerization.** (**A**) Multiple sequence alignment shows a conserved motif in 14–3–3ζ across species (human, mouse, rat, sheep, pig). A potential phosphorylation motif in 14–3–3ζ was predicted by using kinase prediction tool (https://services.healthtech.dtu.dk/service.php?NetPhos-3.1). Ser 58 in 14–3–3ζ was predicted as a potential phosphorylation site. (**B**) Effect of KB1541 on ATP synthase 5 alpha/beta dimerization *in vivo*. Cells were transfected with pCMV–Myc–14–3–3ζ (WT) or pCMV–Myc–14–3–3ζ (S58A) and treated with DMSO or KB1541. Then, immunoprecipitation was performed with an antibody against Myc-tag. 14–3–3ζ (WT) or 14–3–3ζ (S58A) phosphorylation was detected by a phospho-Ser antibody. 14–3–3ζ (WT) phosphorylation was increased upon KB1541 treatment (blue boxed areas). However, 14–3–3ζ (S58A) phosphorylation did not increase upon KB1541 treatment (red boxed areas). 14–3–3ζ (WT) and ATP synthase 5 alpha interaction increased upon KB1541 treatment. The subsequent interaction of 14–3–3ζ (WT) with ATP synthase 5 beta also increased. However, 14–3–3ζ (S58A) and ATP synthase 5 alpha interaction was not affected by KB1541 treatment, nor was the interaction of 14–3–3ζ (S58A) with ATP synthase 5 beta. (**C** and **D**) OXPHOS portion and glycolytic portion of ATP production (^*^P < 0.05, ^**^P < 0.01, student *t*-test). Mean ± S.D., *n* = 3.

14–3–3ζ is an adapter protein that affects a variety of biological processes including proliferation, metabolism, and adhesion [19]. A recent study has shown that 14–3–3ζ regulates mitochondrial ATP synthesis in a non-redundant manner [19]. However, the mechanism through which 14–3–3ζ regulates mitochondrial ATP synthesis remains unknown. 14–3–3ζ interacts with various intracellular molecules and its phosphorylation is thought to play a regulatory role [20]. The identification of a novel phosphorylation site in 14–3–3ζ may aid in elucidating its role in mitochondrial ATP production; thus, we predicted a potential phosphorylation motif in 14–3–3ζ by using kinase prediction tool (https://services.healthtech.dtu.dk/service.php?NetPhos-3.1). Serine (Ser) 58 in 14–3–3ζ was predicted as a potential phosphorylation motif, which is conserved between species (Figure 4A). To validate this prediction, we introduced a point mutation of serine to alanine (S58A) in 14–3–3ζ. Cells were transfected with pCMV–Myc–14–3–3ζ (WT) or pCMV–Myc–14–3–3ζ (S58A), and treated with DMSO or KB1541. Immunoprecipitation was performed with an antibody against Myc-tag. Then, 14–3–3ζ (WT) or 14–3–3ζ (S58A) phosphorylation was detected by a phospho-Ser antibody. 14–3–3ζ (WT) phosphorylation was increased upon KB1541 treatment (Figure 4B; blue boxed areas). However, 14–3–3ζ (S58A) phosphorylation did not increase upon KB1541 treatment, suggesting that KB1541 phosphorylates 14–3–3ζ at Ser 58 (Figure 4B; red boxed areas).

ATP synthase 5 is an energy-generating protein and synthesizes ATP through the assembly of multiple ATP synthase subunits [12]. ATP synthase 5 alpha and ATP synthase 5 beta subunits form an alpha/beta heterodimer, which is crucial for formation of a peripheral stator in F_1_ domain [12]. As protein phosphorylation regulates protein-protein interactions [21], we hypothesized that 14–3–3ζ Ser 58 phosphorylation functions as a post-translational modification facilitating the dimerization of ATP synthase 5 alpha and ATP synthase 5 beta. Thus, we assessed the interaction of 14–3–3ζ with ATP synthase 5 alpha and ATP synthase 5 beta. 14–3–3ζ (WT) and ATP synthase 5 alpha interaction increased upon KB1541 treatment (Figure 4B). Furthermore, 14–3–3ζ (WT) and ATP synthase 5 beta interaction increased upon KB1541 treatment (Figure 4B). However, 14–3–3ζ (S58A) and ATP synthase 5 alpha interaction was not affected by KB1541 treatment, nor was the interaction of 14–3–3ζ (S58A) with ATP synthase 5 beta (Figure 4B). Together, these results imply that KB1541 regulates ATP synthase 5 alpha/beta assembly through 14–3–3ζ phosphorylation at Ser 58.

ATP synthase 5 is a rotary motor that mediates ATP synthesis [22]; thus, we conjectured that KB1541 might regulate ATP synthase 5 function. We then evaluated the effect of KB1541-mediated 14–3–3ζ regulation on ATP production. We measured the oxidative phosphorylation (OXPHOS) portion of ATP generation to identify the ATP production in the mitochondria, whereas the glycolysis portion of ATP generation was measured to quantify the amount of ATP produced during the glycolysis process (Figure 4C and 4D). KB1541 treatment significantly increased the OXPHOS portion of ATP generation but decreased glycolytic portion, indicating that KB1541 improved the efficiency of OXPHOS during ATP synthesis in mitochondria.

### KB1541 ameliorates senescence phenotypes

**Figure 5:**
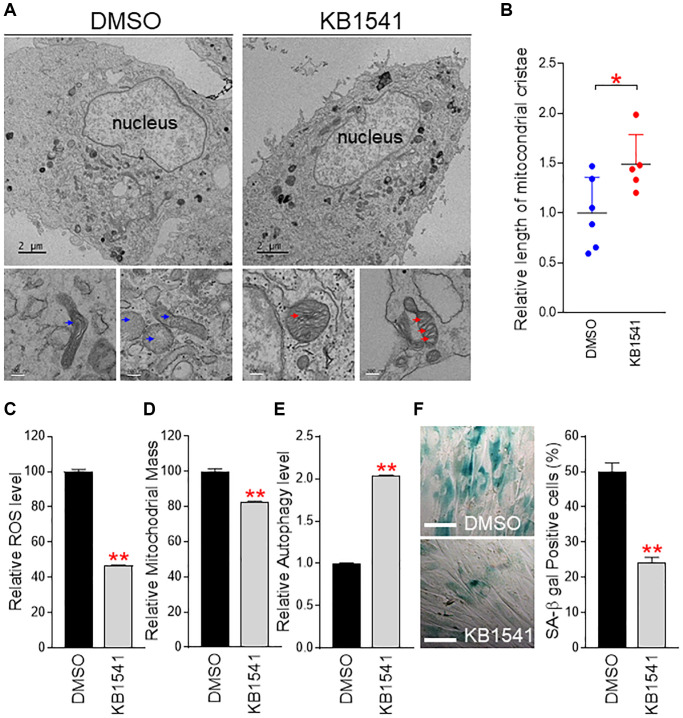
**KB1541 ameliorates senescence phenotypes.** (**A**) Electron microscopy analysis revealed that DMSO-treated senescent fibroblasts exhibited less cristae wrinkle folds (blue arrow), whereas KB1541 treatment in senescent fibroblasts showed increased the number of cristae wrinkle folds (red arrow). Upper image: Scale bar 2 μm. Lower image: Scale bar 200 nm. (**B**) Comparison of cristae length between DMSO and KB1541-treated senescent fibroblasts. ^*^P < 0.05, student *t*-test. Mean ± S.D., *n* = 6. (**C** and **D**) Flow cytometric analysis of ROS and mitochondrial mass using MitoSOX and MitoTracker green, respectively. ^**^P < 0.01, student *t*-test. Mean ± S.D., *n* = 3. Flow cytometry data of ROS and mitochondrial mass were presented in Supplementary Figure 4A. (**E**) Flow cytometric analysis of autophagy level using Cyto–ID assay. ^**^P < 0.01, student *t*-test. Mean ± S.D., *n* = 3. Flow cytometric data of autophagy level were presented in Supplementary Figure 4B. (**F**) Quantification of SA-β gal positive cells. ^**^P < 0.01, student *t*-test. Means ± S.D., *n* = 4. Scale bar 100 μm.

Mitochondrial cristae give the mitochondrial IM a unique wrinkle shape, enabling a lot of surface area for OXPHOS [23]. Mitochondrial cristae contain major OXPHOS components including ATP synthase 5 and various cytochromes [23]. The highly curved nature of mitochondrial cristae increases the IM surface to accommodate electron transport system in OXPHOS. Thus, we evaluated the effect of KB1541 on the morphology of mitochondrial cristae by using electron microscope. Electron microscopy analysis revealed that DMSO-treated senescent fibroblasts exhibited less cristae wrinkle folds (Figure 5A, blue arrow), whereas KB1541 treatment in senescent fibroblasts exhibited an increase in the number of cristae wrinkle folds (Figure 5A, red arrow). As the number of cristae wrinkle folds was indirectly assessed by the length of mitochondrial cristae [24], we used Image J analysis to quantify the length of mitochondrial cristae and saw that KB1541 treatment significantly increased the length of mitochondrial cristae (Figure 5B).

Dysfunctional mitochondria induce excessive reactive oxygen species (ROS) production that causes severe damage to organelles [25]. As we observed the increase in OXPHOS portion of ATP generation but the decrease in glycolysis portion as an energy source, which is the prerequisite phenomenon for mitochondrial functional recovery [26, 27], we hypothesized that KB1541 treatment would reduce ROS generation. Thus, we measured ROS levels and found that ROS levels were significantly reduced upon KB1541 treatment (Figure 5C and Supplementary Figure 4A).

Senescent fibroblasts exhibit an increase in mitochondrial mass and size, suggesting a causative role in senescence [28, 29]. This increase is a compensatory response to the functional reduction of mitochondria as a result of ROS damage [30]. Thus, we examined the mitochondrial mass by using MitoTracker green. Upon KB1541 treatment, mitochondrial mass was significantly reduced (Figure 5D and Supplementary Figure 4A).

The autophagy system is a critical route for the breakdown of dysfunctional mitochondria [31]. Restoration of autophagic function constitutes one of criteria for senescence amelioration [32]. As we observed the significantly decreased mitochondrial mass upon KB1541 treatment, we examined the status of autophagic function. Thus, we measured autophagy levels by using Cyto–ID staining solution. Upon KB1541 treatment, autophagic function was significantly increased, suggesting the KB1541-mediated recovery of autophagy system (Figure 5E and Supplementary Figure 4B).

SA-β-gal activity has been widely used as a surrogate marker for senescence [33]. The proportion of SA-β-gal positive cells was significantly decreased after KB1541 treatment (Figure 5F). Activation of p53 and its downstream target p21 is pivotal in the induction of cell cycle arrest, a hallmark of senescence [34]. KB1541 reduced the levels of phosphorylated p53 (p-p53) and p21, suggesting the KB1541-mediated reduction of senescence marker proteins (Supplementary Figure 5A). Finally, as one of the most pronounced senescence phenotypes is an increase in cell surface area [25], morphological changes of senescent fibroblast were examined after KB1541 treatment. Senescent fibroblasts treated with DMSO showed a large and flat structure (dotted lines), whereas senescent fibroblasts treated with KB1541 showed a small spindle shape (red arrows), which was one of the prominent features seen in young cells (Supplementary Figure 5B).

### Maintenance of *14–3–3ζ* expression is a prerequisite for KB1541-mediated senescence amelioration

**Figure 6:**
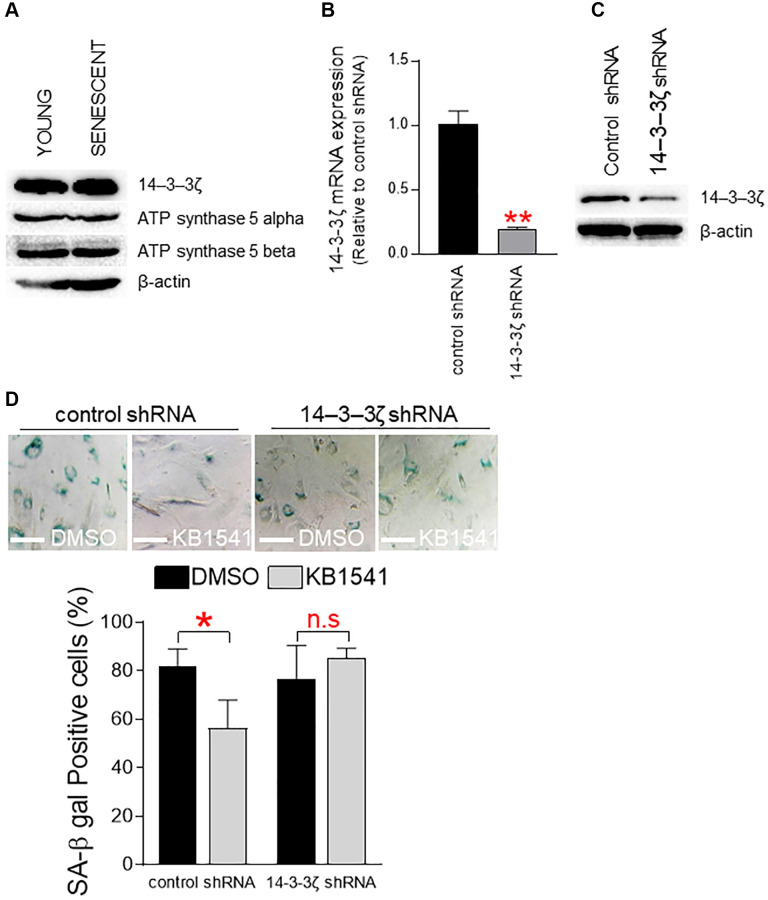
**Maintenance of *14–3–3ζ* expression is a prerequisite for KB1541-mediated senescence alleviation.** (**A**) Expression levels of 14–3–3ζ and ATP synthase 5 alpha/beta proteins in young and senescent fibroblasts. (**B**) shRNA-mediated *14–3–3ζ* knockdown significantly reduced the endogenous expression level of 14–3–3ζ mRNA. ^**^P < 0.01, student *t*-test. Mean ± S.D., *n* = 3. (**C**) Immunoblot of total lysates from senescent cells transduced with lenti-virus expressing control shRNA or 14–3–3ζ shRNA. (**D**) Quantification of SA-β gal positive cells. ^*^P < 0.05, student *t*-test. Mean ± S.D., *n* = 3.

To determine the role of 14–3–3ζ on senescence, we evaluated expression levels of 14–3–3ζ and ATP synthase 5 alpha/beta proteins in senescent and young fibroblasts. Expression levels of 14–3–3ζ and ATP synthase 5 alpha/beta proteins in senescent fibroblasts were similar to those in young fibroblasts, indicating that senescence does not affect the expression of these proteins (Figure 6A). We then examined the effect of shRNA-mediated 14–3–3ζ knockdown on KB1541-induced senescence amelioration. Senescent fibroblasts were transduced with lenti-virus expressing 14–3–3ζ shRNA or control shRNA. shRNA-mediated 14–3–3ζ knockdown significantly decreased the endogenous expression levels of *14–3–3ζ* mRNA and 14–3–3ζ protein (Figure 6B and 6C). Furthermore, shRNA-mediated 14–3–3ζ knockdown rendered senescent fibroblasts unresponsive to KB1541, as shown to fail to reduce SA-β-gal positive cells (Figure 6D). However, senescent cells transduced with lenti-virus expressing control shRNA responded to KB1541 as shown by significant decrease in SA-β-gal positive cells (Figure 6D). Taken together, these data suggest that the maintenance of *14–3–3ζ* expression is a prerequisite for KB1541-mediated senescence alleviation.

## DISCUSSION

In biological systems, ATP is a key energy storage molecule, and inhibition of ATP production induces senescence, as evidenced by increased p53 and p21 expression [35]. Furthermore, a decrease in ATP production creates a bioenergetic imbalance by increasing the AMP (or ADP) to ATP ratio, resulting in cellular growth arrest and upregulation of senescence phenotypes [36]. ATP synthase 5 is present in the cristae of mitochondrial IM and consists of two structural domains: F_1_ and F_0_. F_1_ domain contains a membrane proton channel and F_0_ domain is linked together by the central stem and the peripheral stem [11]. Specifically, F_1_ domain consists of three ATP synthase 5 alpha subunits and three ATP synthase 5 beta subunits, yielding α3β3 hexamer [37]. Atp12p and Atp11p chaperones assists the assembly of α3β3 hexamer [38]. Specifically, Atp12p and Atp11p bind to the alpha and beta subunits, respectively, to form the heterodimers Atp12p-alpha and Atp11p-beta [39]. However, the fine-tuning mechanism regulating ATP synthase 5 alpha/beta heterodimerization is still poorly identified. In the present study, we uncovered a mechanism in which KB1541 regulates ATP synthase 5 alpha/beta heterodimerization by 14–3–3ζ Ser 58 phosphorylation. Specifically, phosphorylation of Ser 58 in 14–3–3ζ serves as a posttranslational modification site linking ATP synthase 5 alpha and beta subunits. Upon KB1541 treatment, 14–3–3ζ functions as an adapter protein to induce alpha/beta heterodimerization. Our findings therefore suggest that KB1541 regulates ATP synthase 5 alpha/beta heterodimerization by direct phosphorylation of 14–3–3ζ protein at Ser 58. Based on our findings, we propose that the proper regulation of ATP synthase 5 alpha/beta heterodimerization by KB1541 would be effective for the treatment of aging and age-related diseases, although further studies are required to confirm this outcome.

The regulation of senescence is inextricably linked with the maintenance of mitochondrial metabolism. The deterioration of mitochondrial metabolism is an age-dependent process, since cells from aged animals have been shown to be more reliant on glycolysis as a source of energy [40]. Support for this result is evident in the observation that severe mitochondrial dysfunction was found in senescent fibroblasts with a decrease in OXPHOS activity and an increase in glycolysis dependence [41]. Furthermore, alterations in mitochondrial metabolism reduced cell proliferation and contributed to premature aging of human fibroblasts [35]; however, how these changes can be delayed or prevented has not been well investigated. In the current study, we identified a small compound KB1541 as a candidate that mediates a novel mechanism controlling senescence by upregulating ATP synthase 5 alpha/beta heterodimerization. Increased assembly enhanced the OXPHOS portion of ATP generation but decreased glycolytic portion, indicating KB1541-mediated metabolic reprogramming. Furthermore, modulation of mitochondrial metabolism by KB1541 restored mitochondrial function, accompanied by recovery of senescent phenotypes, suggesting that KB1541-mediated mitochondrial metabolic reprogramming induced amelioration of senescence. Our study is the first to demonstrate that KB1541 enhances the efficiency of OXPHOS, which is lower in senescent fibroblasts. Extending the relevance of these findings, the enhanced OXPHOS efficiency is accompanied by amelioration of senescent phenotypes, rendering targeting mitochondrial metabolic reprogramming as a potential treatment method for senescence.

Mitochondrial dysfunction has been considered both target and cause of senescence [42]. The main function of mitochondria is highly dependent on the mitochondrial ATP production efficiency [43]. To this end, mitochondrial cristae give the mitochondrial IM a wrinkled appearance, allowing chemical reactions to occur over a large surface area [44]. ATP synthase 5 and cytochrome complexes (COX 1–4) make up the mitochondrial IM [45] and are concentrated in the cristae of the mitochondrial IM [11]. Despite the fact that the role of cristae formation in mitochondrial function has received a lot of attention [46], a consensus on mechanism to increase cristae formation has not been achieved. A current study suggests that caloric restriction increases the number of mitochondrial cristae by preventing an excitotoxic state through an indirect decrease in mitochondrial permeability and calcium retention [47]. Here, we observed that KB1541 also induced an increase in cristae length, allowing more surface area resulting in an increased capacity for ATP generation. The increase in mitochondrial cristae length by KB1541 could be explained by previous findings showing that the increase in ATP generation exerted beneficial effects in mitochondrial function including increases in calcium buffering capacity and decrease in overall ROS production [48]. Extending the relevance of these observations, KB1541 treatment facilitated the mitochondrial functional recovery resulting in reduced ROS generation and decreased mitochondrial mass. Taken together, the results from our study suggest that KB1541 exerts beneficial effects on mitochondrial functional recovery by increasing the mitochondrial cristae length and decreasing the ROS/mitochondrial mass.

Senescence is a multifactorial process that may accompany inflammation and immune dysfunction [49]. Senescent cells secrete inflammatory cytokines (characterized by IL-1β, IL-6, IL-8 and IL-33) that lead to chronic inflammation and fibrosis [4]. IL-33, a cytokine belonging to the IL-1 family, is expressed in epithelial cells and secreted into the extracellular space [5]. Many reports have highlighted the finding that the increase in IL-33 expression is one of the most characteristic changes during senescence. For example, IL-33 expression in astrocytes was considerably elevated by up to 74% in aged mice [50]. Moreover, microarray analysis revealed age-dependent changes in IL-33 expression as indicated by increased IL-33 expression in aged mice compared to young mice [51]. IL-33 is also strongly associated with neuroinflammation in age-related diseases including Alzheimer's disease and multiple sclerosis [52, 53]. Thus, therapeutic strategies to reduce IL-33 have been considered as novel targets for therapeutic intervention in patients with aging and age-related diseases. In the present study, we identified that IL-33 inhibitor KB1541 mediates novel mechanisms to control senescence through regulating ATP synthase 5 alpha/beta heterodimerization by 14–3–3ζ Ser 58 phosphorylation. However, we acknowledged that further studies are required to elucidate the direct relationship of IL-33 with mitochondrial energy metabolism and to investigate whether other IL-33 inhibitors can modulate senescence by the mechanisms we found. These findings will provide an a priori basis for therapeutic strategies using IL-33 inhibitors in patients with aging and age-related diseases.

**Figure 7:**
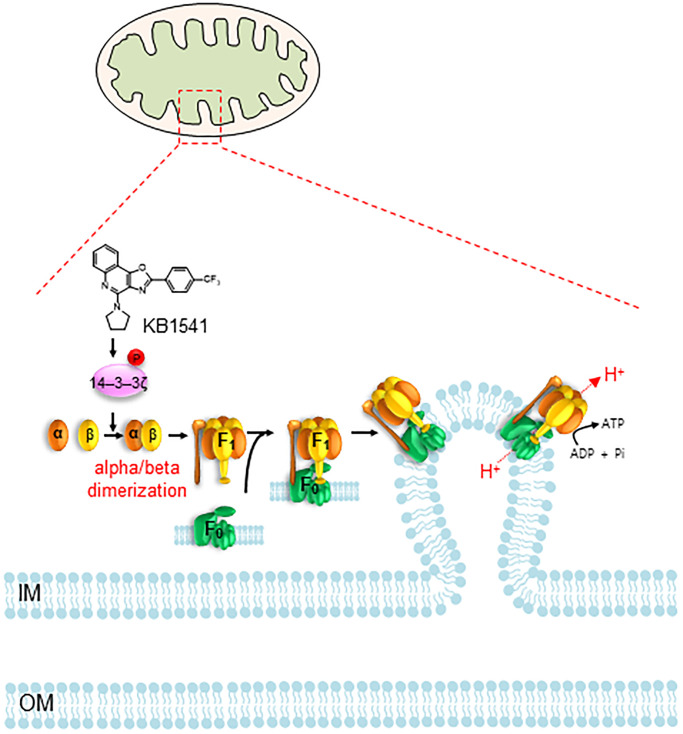
**Proposed mechanism showing how KB1541 controls OXPHOS efficiency by regulating ATP synthase alpha/beta dimerization via phosphorylation of 14–3–3ζ protein.** Abbreviations: IM: inner membrane; OM: outer membrane.

In summary, we identified IL-33 inhibitor KB1541 as a potential module for alleviating senescence. We discovered that KB1541 controls OXPHOS efficiency by regulating ATP synthase 5 alpha/beta dimerization through the direct phosphorylation of 14–3–3ζ protein at Ser 58 (Figure 7). The induced mitochondrial metabolic reprogramming from glycolysis to OXPHOS as an energy source led to a functional recovery of mitochondria, which in turn accelerated the increase in mitochondrial cristae length and decrease in ROS formation. Taken together, our study provides evidence that the fine-tuning of ATP synthase 5 alpha/beta dimerization by KB1541 can induce mitochondrial functional recovery, concomitant recovery of senescent phenotypes, rendering the use of KB1541 as a potentially advantageous therapeutic strategy in aging and age-related diseases.

## MATERIALS AND METHODS

### Synthesis of KB1541 and biotinylated KB1541

Synthetic methods, experimental procedures, and analytical data of KB1541 and biotinylated KB1541 are described in detail in Supplementary Materials.

### Cell culture

Human diploid fibroblasts (HDF; PCS–201–010; ATCC, Manassas, VA, USA) and Human embryonic kidney cells (HEK293T; CRL–11268; ATCC) were used in this study. Cells were cultured in Dulbecco’s modified Eagle’s medium containing 25 mM glucose supplemented with 10% fetal bovine serum (SH30919.03; Hyclone, Waltham, MA, USA) and 100 U/ml penicillin and 100 μg/ml streptomycin (SV30079.01; Hyclone). Cells were passaged serially at a 1:4 dilution during early passages and a 1:2 during late passages. When the population doubling time of the cells was over 14 days and less than 2 days, the cells were considered senescent and young, respectively. Cell viability was assessed using Cedex HiRes Analyzer (05650216001; Roche, Basel, Switzerland).

### Compound screening

Senescent fibroblasts were grown in 96–well plates at a density of 1,000 cells per well. Components from the library were diluted to a final concentration of 4 μM and added to the wells every 4 days. 12 days after drug treatment, cells were washed twice with phosphate–buffered saline (PBS) and lysed in 50 μl of 0.2% SDS. The plates were incubated at 37°C for 1 h. GelGreen^®^ Nucleic Acid Gel Stain (150 μl) (1:1,000 in DW; 41005; Biotium, Fremont, CA, USA) was added to the wells. Cell number was determined by measuring fluorescence intensity with VICTOR Multilabel Plate Reader (2030–0050; PerkinElmer, Waltham, MA, USA). The mean and standard deviation from six replicates were determined for each experimental group.

### IM–MS/MS TOF analysis

Senescent fibroblasts were treated for 14 days with 8 μM biotinylated KB1541. Immunoprecipitation was performed using AccuNanoBead™ Streptavidin Magnetic Nanobeads (TA-1015-1; Bioneer, Daejon, Korea). The eluted proteins were applied on to an Ion Mobility Tandem Mass Spectrometer/Time-of-flight mass spectrometry (IM–MS/MS TOF) (LCQ Deca XP-Plus, Thermo Finnigan, San Jose, CA, USA). Data analysis was performed as previously described [54].

### Plasmid construction

Plasmid pCMV–Myc–ATP synthase 5 alpha was constructed by inserting cDNA encoding human ATP synthase 5 alpha (GenBank accession NM_001001935) into the pCMV–Myc vector (631604; Clontech, Mountain View, CA, USA). Plasmid pCMV–Myc–ATP synthase 5 beta was constructed by inserting cDNA encoding human ATP synthase 5 beta (GenBank accession NM_001686) into the pCMV–Myc vector. Plasmid pCMV–Myc–14–3–3ζ was constructed by inserting cDNA encoding human 14–3–3ζ (GenBank accession NM_001135699) into the pCMV–Myc vector. Plasmid pCMV–Myc–14–3–3ζ (S58A) was constructed using a HiFi DNA Assembly Master Mix (E2621L; New England Biolabs, Ipswich, MA, USA). The following sense and anti-sense oligonucleotide pairs were used to introduce S58A mutation (5′-GCATGGAGGGTCGTCTCAAGTATTGAACAAAAGAC-3′ and 5′-GACGACCCTCCATGCTGACCTACGGGCTCCTACAA-3′). Plasmid pcDNA3.1 His-14–3–3ζ was constructed by inserting cDNA encoding human 14–3–3ζ (GenBank accession NM_001135699) into the pcDNA3.1 His vector (V38520; Invitrogen, Carlsbad, CA, USA).

### *In vivo* pull-down assay

pCMV–Myc–ATP synthase 5 alpha, pCMV–Myc–ATP synthase 5 beta, or pCMV–Myc–14–3–3ζ plasmids were transfected into HEK293T cells (CRL–11268; ATCC). Transfected cells were selected with 3 μM puromycin (BML-GR312-0050; Enzo Life Sciences, Farmingdale, NY, USA) for 14 days. During selection, the medium was changed every 4 day. After selection, cells were treated with 8 μM biotinylated KB1541 for 5 days. Cells were lysed with lysis buffer [50 mM Tris-HCl (pH 7.4), 150 mM KCl, 1 mM PMSF, 2 mM benzamidine, 0.05% NP-40, protease inhibitor cocktail (04693116001; Roche)]. Then, precipitation was performed using AccuNanoBead™ Streptavidin Magnetic Nanobeads (TA-1015-1; Bioneer). Proteins were separated on a 10% SDS–PAGE gel and analyzed by immunoblotting.

### *In silico* docking studies

#### Ligand preparation and optimization

All ligands were generated as 2D and 3D structure by ChemBioDraw (ver. 11.0.1, PerkinElmer) and Chem3D Pro (ver. 11.0.1, PerkinElmer), respectively. All ligands were prepared and optimized using ‘Sanitize’ protocol in SYBYL-X 2.1.1 (Tripos Inc., St Louis, MO, USA) to clean up of the structures involving filling valences, standardizing, removing duplicates and producing only one molecule per input structure.

#### Protein preparation

ATP synthase 5 and 14–3–3ζ structures in PDB format were downloaded from RCSB protein data bank (PDB ID: 2JDI for ATP synthase 5 alpha and beta subunits; 6FN9 for 14–3–3ζ). SYBYL-X 2.1.1 program was employed for protein preparation including conflicted side chains of amino acid residues fixation. Hydrogen atoms were added using TRIPOS Force Field application for both proteins. Minimization process was performed by POWELL method and initial optimization option was set to None for both proteins. Termination gradient and max iteration were set at 0.05 kcal/(mol*Å) and 100 times respectively, for both proteins.

#### Docking and scoring function studies

The docking studies of all prepared ligands were performed by Surflex-Dock GeomX module in SYBYL-X 2.1.1. For ATP synthase 5 alpha and beta subunits, docking was guided by the Surflex-Dock protomol and docking site was defined by the ‘Ligand’ method with the reported ligand phosphoaminophosphonic acid-adenylate ester. For 14–3–3ζ, Surflex-Dock protomol was set to ‘Residues’ method with selected amino acids based on the active sites of 14–3–3ζ (Lys49, Gly53, Arg56, Arg60, Arg127, Tyr128, Glu131, Leu172, Asn173, Val176, Leu220; radius setting: 2.0) was used to guide docking site. Two factors related with a generation of protomol; Bloat (Å) and Threshold were set to 0.5 and 0, respectively. Other parameters were applied with its default settings in all runs.

### Microscale thermophoresis (MST) assay

His-tagged 14–3–3ζ proteins were purified using the Capturem™ His-Tagged Purification Maxiprep Kit (635713; Takara, Mountain View, CA, USA). 1.5 μM of His-tagged 14–3–3ζ protein was labeled using Monolith His-Tag Labeling Kit RED-tris-NTA 2nd Generation (MO-L018; Nano Temper Technologies, München, Germany). MST assay was performed as described previously [55].

### Immunoprecipitation

pCMV–Myc–14–3–3ζ (WT) or pCMV–Myc–14–3–3ζ (S58A) plasmids were transfected into HEK293T cells (CRL–11268; ATCC). Transfected cells were selected with 3 μM puromycin (BML-GR312-0050; Enzo Life Sciences) for 14 days. During selection, the medium was changed every 4 days. After selection, cells were treated with 8 μM biotinylated KB1541 for 5 days. Cells were lysed with lysis buffer [50 mM Tris-HCl (pH 7.4), 150 mM KCl, 1 mM PMSF, 2 mM benzamidine, 0.05% NP-40, protease inhibitor cocktail (04693116001; Roche)]. Then, immunoprecipitation was performed using anti–Myc tag antibody (2276s; cell signaling). The following immunoprecipitation assay was performed as described [56]. Proteins were separated on a 10% SDS–PAGE gel and analyzed by immunoblotting.

### Western blot analysis

Western blotting was performed as previously described [57]. Proteins were detected with the SuperSignal™ West Pico chemiluminescence solution (34577; Thermo Fisher Scientific, Waltham, MA, USA) using a ChemiDoc XRS+ system (1708265; BIO-RAD; Hercules, CA, USA). The primary antibodies used in this study included anti–Myc tag antibody (2276s; 1:200 dilution; cell signaling), anti-ATP synthase 5 alpha antibody (sc136178; 1:500 dilution, Santa Cruz Biotechnology, Dallas, TX, USA), anti- ATP synthase 5 beta antibody (sc55597; 1:500 dilution, Santa Cruz Biotechnology), anti-phosphoserine (phospho-Ser) antibody (05–1000; 1:200 dilution, Millipore, Burlington, MA, USA), and HRP–conjugated β–actin (sc47778; 1:1000 dilution; Santa Cruz Biotechnology, Dallas, TX, USA), anti-p21 antibody (sc-6246; 1:500 dilution, Santa Cruz), anti-phospho-p53 antibody (sc-377561; 1:500 dilution, Santa Cruz), anti-14–3–3ζ antibody (sc-1657; 1:500 dilution, Santa Cruz). The secondary antibodies used in this study included HRP-conjugated anti-rabbit IgG (sc-2004; 1:1,000 dilution; Santa Cruz Biotechnology) and HRP-conjugated anti-mouse IgG (sc-2302; 1:1,000 dilution; Santa Cruz Biotechnology).

### Measurement of cellular ATP levels

Cells were incubated in medium with or without 20 μM oligomycin for 24 h. ATP content was measured using a ViaLight Plus Kit (LT07–221; Lonza, Basel, Switzerland) according to the manufacturer’s instructions. DNA content was measured using AccuBlue broad range dsDNA quantitation kit (31007; Biotium, Fremont, CA, USA). For measurements of relative ATP content, the luminescence of each sample was normalized to the DNA content.

### Electron microscopy (EM)

Senescent fibroblasts were treated with or without 4 μM KB1541 for 21 days prior to electron microscopy analysis. Cells were separated with trypsin-EDTA (25300–054; Gibco, Waltham, MA, USA), washed with PBS (LB004–02; WelGene, Gyeongsan, Korea), and fixed with 2.5% glutaraldehyde (340855; Sigma–Aldrich, St. Louis, MO, USA) in 0.1 M PBS (pH 7.4) for 2 h. EM images were observed with a Bio-transmission electron microscope (Tecnai™ G2 Spirit; FEI company, Hillsboro, OR, USA). To quantify the length of mitochondrial cristae, Image J analysis was performed. Cristae length was measured as the sum of the inwardly extending portion of the mitochondrial lining and the circular double membrane in the mitochondria.

### Measurement of reactive oxygen species (ROS) and mitochondrial mass

For quantification of mitochondrial ROS, the cells were incubated in medium containing 5 μM MitoSOX (M36008; Life Technologies, Carlsbad, CA, USA) for 30 min at 37°C. For quantification of mitochondrial mass, the cells were incubated in medium containing 50 nM MitoTracker green (M7514; Life Technologies) for 30 min at 37°C. After staining, cells were prepared for FACS analysis as previously described [58].

### Measurement of autophagic level

Cells were stained with Cyto–ID staining solution (ENZ–51031–0050; Enzo Life Sciences, Lausen, Switzerland) and 50 nM LysoTracker deep red (LTDR) (L12492; Life Technologies) for 30 min and prepared for FACS analysis. To measure background autofluorescence, cells were incubated in medium without dye. Fluorescence from Cyto–ID was normalized with fluorescence from LTDR.

### Senescent associated-β-galactosidase (SA-β-gal) staining

SA-β-gal staining was performed according to the manufacturer’s protocols (9860; Cell Signaling Technology, Beverly, MA, USA).

### Lenti-viral shRNA production and infection

HEK 293T cells were transfected with 10 μg shRNA plasmid (control shRNA or 14–3–3ζ shRNA (sc-156019-SH; Santa Cruz Biotechnology), 5 μg PAX2 plasmid, and 5 μg VSV-G plasmid using Lipofectamine 2000 (11668019; Invitrogen). Viral supernatant was harvested 24 h after transfection. Polybrene (TR-1003-G; Millipore; 6 μg/ml) was added into viral supernatant. Virus infection was performed as previously described [26].

### Preparation of cDNA (complementary DNA)

Total RNA was isolated from 5 × 10^5^ cells using RNase Mini Kit (74104; QIAGEN, Hilden, Germany) according to the manufacturer’s instructions. Total RNA was reverse-transcribed using a DiaStar™ RT Kit (DR22-R10k; SolGent, Seoul, Korea) according to the manufacturer’s instructions. The purity and concentration of the cDNA were determined using a DS-11 Spectrophotometer (DS-11; DeNovix, Wilmington, DE, USA).

### Quantitative polymerase chain reaction (qPCR)

qPCR was performed using a Solg™ 2× real-time PCR smart mix (SRH83-M40h; Solgent) in a CFX Connect™ Real-Time PCR Detection System (Bio-Rad, Hercules, CA, USA). qPCR was performed with denaturation at 95°C for 4 min followed by 40 cycles of 94°C for 30 s, 57°C for 30 s, and 70°C for 10 s. qPCR was done using the following primer: 5′-CGCAATGGTGAAGGTC-3′ (*GAPDH*-forward), 5′-CGCCAGCATCACCCC-3′ (*GAPDH*-reverse); 5′-AAGACGAAGGTGCTG-3′ (*14–3–3ζ*-forward), 5′-GCTTGTGAAGCATTGGG-3′ (*14–3–3ζ*-reverse).

### Statistical analysis

Statistical analysis were performed using a standard statistical software package (SigmaPlot 12.5; Systat Software, San Jose, CA, USA). The Student’s *t*-test and two-way ANOVA followed by Bonferroni’s post test were used to determine whether differences were significant.

## Supplementary Materials

Supplementary Methods

Supplementary Figures

Supplementary Tables 1 and 2

Supplementary Table 3

## References

[1] Hayflick L. The limited in vitro lifetime of human diploid cell strains. Exp Cell Res. 1965; 37:614–36. 10.1016/0014-4827(65)90211-9 14315085

[2] Childs BG, Durik M, Baker DJ, van Deursen JM. Cellular senescence in aging and age-related disease: from mechanisms to therapy. Nat Med. 2015; 21:1424–35. 10.1038/nm.4000 26646499 PMC4748967

[3] Coppé JP, Desprez PY, Krtolica A, Campisi J. The senescence-associated secretory phenotype: the dark side of tumor suppression. Annu Rev Pathol. 2010; 5:99–118. 10.1146/annurev-pathol-121808-102144 20078217 PMC4166495

[4] Ito Y, Hoare M, Narita M. Spatial and Temporal Control of Senescence. Trends Cell Biol. 2017; 27:820–32. 10.1016/j.tcb.2017.07.004 28822679

[5] Miller AM. Role of IL-33 in inflammation and disease. J Inflamm (Lond). 2011; 8:22. 10.1186/1476-9255-8-22 21871091 PMC3175149

[6] Mirchandani AS, Salmond RJ, Liew FY. Interleukin-33 and the function of innate lymphoid cells. Trends Immunol. 2012; 33:389–96. 10.1016/j.it.2012.04.005 22609147

[7] Carlock C, Wu J, Shim J, Moreno-Gonzalez I, Pitcher MR, Hicks J, Suzuki A, Iwata J, Quevado J, Lou Y. Interleukin33 deficiency causes tau abnormality and neurodegeneration with Alzheimer-like symptoms in aged mice. Transl Psychiatry. 2017; 7:e1191. 10.1038/tp.2017.176 28675392 PMC5538122

[8] Theodoropoulou S, Copland DA, Liu J, Wu J, Gardner PJ, Ozaki E, Doyle SL, Campbell M, Dick AD. Interleukin-33 regulates tissue remodelling and inhibits angiogenesis in the eye. J Pathol. 2017; 241:45–56. 10.1002/path.4816 27701734 PMC5683707

[9] Kim Y, Ma C, Park S, Shin Y, Lee T, Paek J, Hoon Kim K, Jang G, Cho H, Son S, Son SH, Yong Lee K, Lee K, et al. Rational Design, Synthesis and Evaluation of Oxazolo[4,5-c]-quinolinone Analogs as Novel Interleukin-33 Inhibitors. Chem Asian J. 2021; 16:3702–12. 10.1002/asia.202100896 34553505

[10] Sherratt HS. Mitochondria: structure and function. Rev Neurol (Paris). 1991; 147:417–30. 1962047

[11] Kühlbrandt W. Structure and function of mitochondrial membrane protein complexes. BMC Biol. 2015; 13:89. 10.1186/s12915-015-0201-x 26515107 PMC4625866

[12] Féthière J, Venzke D, Diepholz M, Seybert A, Geerlof A, Gentzel M, Wilm M, Böttcher B. Building the stator of the yeast vacuolar-ATPase: specific interaction between subunits E and G. J Biol Chem. 2004; 279:40670–6. 10.1074/jbc.M407086200 15292229

[13] Silva LP, Lorenzi PL, Purwaha P, Yong V, Hawke DH, Weinstein JN. Measurement of DNA concentration as a normalization strategy for metabolomic data from adherent cell lines. Anal Chem. 2013; 85:9536–42. 10.1021/ac401559v 24011029 PMC3868625

[14] Landis GN, Bhole D, Tower J. A search for doxycycline-dependent mutations that increase Drosophila melanogaster life span identifies the VhaSFD, Sugar baby, filamin, fwd and Cctl genes. Genome Biol. 2003; 4:R8. 10.1186/gb-2003-4-2-r8 12620118 PMC151307

[15] Hughes AL, Gottschling DE. An early age increase in vacuolar pH limits mitochondrial function and lifespan in yeast. Nature. 2012; 492:261–5. 10.1038/nature11654 23172144 PMC3521838

[16] Kane LA, Youngman MJ, Jensen RE, Van Eyk JE. Phosphorylation of the F(1)F(o) ATP synthase beta subunit: functional and structural consequences assessed in a model system. Circ Res. 2010; 106:504–13. 10.1161/CIRCRESAHA.109.214155 20035080 PMC2835499

[17] Aducci P, Camoni L, Marra M, Visconti S. From cytosol to organelles: 14-3-3 proteins as multifunctional regulators of plant cell. IUBMB Life. 2002; 53:49–55. 10.1080/15216540210813 12018408

[18] de Boer AH. Plant 14-3-3 proteins assist ion channels and pumps. Biochem Soc Trans. 2002; 30:416–21. 10.1042/bst0300416 12196106

[19] Schoenwaelder SM, Darbousset R, Cranmer SL, Ramshaw HS, Orive SL, Sturgeon S, Yuan Y, Yao Y, Krycer JR, Woodcock J, Maclean J, Pitson S, Zheng Z, et al. 14-3-3ζ regulates the mitochondrial respiratory reserve linked to platelet phosphatidylserine exposure and procoagulant function. Nat Commun. 2016; 7:12862. 10.1038/ncomms12862 27670677 PMC5052641

[20] Stevers LM, Sijbesma E, Botta M, MacKintosh C, Obsil T, Landrieu I, Cau Y, Wilson AJ, Karawajczyk A, Eickhoff J, Davis J, Hann M, O’Mahony G, et al. Modulators of 14-3-3 Protein-Protein Interactions. J Med Chem. 2018; 61:3755–78. 10.1021/acs.jmedchem.7b00574 28968506 PMC5949722

[21] Tudor CO, Ross KE, Li G, Vijay-Shanker K, Wu CH, Arighi CN. Construction of phosphorylation interaction networks by text mining of full-length articles using the eFIP system. Database (Oxford). 2015; 2015:bav020. 10.1093/database/bav020 25833953 PMC4381107

[22] Forgac M. Vacuolar ATPases: rotary proton pumps in physiology and pathophysiology. Nat Rev Mol Cell Biol. 2007; 8:917–29. 10.1038/nrm2272 17912264

[23] Mannella CA. Consequences of Folding the Mitochondrial Inner Membrane. Front Physiol. 2020; 11:536. 10.3389/fphys.2020.00536 32581834 PMC7295984

[24] Cogliati S, Enriquez JA, Scorrano L. Mitochondrial Cristae: Where Beauty Meets Functionality. Trends Biochem Sci. 2016; 41:261–73. 10.1016/j.tibs.2016.01.001 26857402

[25] Hwang ES, Yoon G, Kang HT. A comparative analysis of the cell biology of senescence and aging. Cell Mol Life Sci. 2009; 66:2503–24. 10.1007/s00018-009-0034-2 19421842 PMC11115533

[26] Kang HT, Park JT, Choi K, Kim Y, Choi HJC, Jung CW, Lee YS, Park SC. Chemical screening identifies ATM as a target for alleviating senescence. Nat Chem Biol. 2017; 13:616–23. 10.1038/nchembio.2342 28346404

[27] Kim JW, Kuk MU, Choy HE, Park SC, Park JT. Mitochondrial metabolic reprograming via BRAF inhibition ameliorates senescence. Exp Gerontol. 2019; 126:110691. 10.1016/j.exger.2019.110691 31421186

[28] Lee HC, Yin PH, Chi CW, Wei YH. Increase in mitochondrial mass in human fibroblasts under oxidative stress and during replicative cell senescence. J Biomed Sci. 2002; 9:517–26. 10.1007/BF02254978 12372989

[29] Passos JF, Saretzki G, Ahmed S, Nelson G, Richter T, Peters H, Wappler I, Birket MJ, Harold G, Schaeuble K, Birch-Machin MA, Kirkwood TB, von Zglinicki T. Mitochondrial dysfunction accounts for the stochastic heterogeneity in telomere-dependent senescence. PLoS Biol. 2007; 5:e110. 10.1371/journal.pbio.0050110 17472436 PMC1858712

[30] Westermann B. Bioenergetic role of mitochondrial fusion and fission. Biochim Biophys Acta. 2012; 1817:1833–8. 10.1016/j.bbabio.2012.02.033 22409868

[31] Wang HL, Chou AH, Wu AS, Chen SY, Weng YH, Kao YC, Yeh TH, Chu PJ, Lu CS. PARK6 PINK1 mutants are defective in maintaining mitochondrial membrane potential and inhibiting ROS formation of substantia nigra dopaminergic neurons. Biochim Biophys Acta. 2011; 1812:674–84. 10.1016/j.bbadis.2011.03.007 21421046

[32] Herranz N, Gil J. Mechanisms and functions of cellular senescence. J Clin Invest. 2018; 128:1238–46. 10.1172/JCI95148 29608137 PMC5873888

[33] Dimri GP, Lee X, Basile G, Acosta M, Scott G, Roskelley C, Medrano EE, Linskens M, Rubelj I, Pereira-Smith O. A biomarker that identifies senescent human cells in culture and in aging skin in vivo. Proc Natl Acad Sci U S A. 1995; 92:9363–7. 10.1073/pnas.92.20.9363 7568133 PMC40985

[34] Rufini A, Tucci P, Celardo I, Melino G. Senescence and aging: the critical roles of p53. Oncogene. 2013; 32:5129–43. 10.1038/onc.2012.640 23416979

[35] Stöckl P, Hütter E, Zwerschke W, Jansen-Dürr P. Sustained inhibition of oxidative phosphorylation impairs cell proliferation and induces premature senescence in human fibroblasts. Exp Gerontol. 2006; 41:674–82. 10.1016/j.exger.2006.04.009 16713693

[36] Zwerschke W, Mazurek S, Stöckl P, Hütter E, Eigenbrodt E, Jansen-Dürr P. Metabolic analysis of senescent human fibroblasts reveals a role for AMP in cellular senescence. Biochem J. 2003; 376:403–11. 10.1042/bj20030816 12943534 PMC1223775

[37] Song J, Pfanner N, Becker T. Assembling the mitochondrial ATP synthase. Proc Natl Acad Sci U S A. 2018; 115:2850–2. 10.1073/pnas.1801697115 29514954 PMC5866614

[38] Ackerman SH, Tzagoloff A. Identification of two nuclear genes (ATP11, ATP12) required for assembly of the yeast F1-ATPase. Proc Natl Acad Sci U S A. 1990; 87:4986–90. 2142305 10.1073/pnas.87.13.4986 PMC54246

[39] Wang ZG, Sheluho D, Gatti DL, Ackerman SH. The alpha-subunit of the mitochondrial F(1) ATPase interacts directly with the assembly factor Atp12p. EMBO J. 2000; 19:1486–93. 10.1093/emboj/19.7.1486 10747017 PMC310218

[40] James EL, Michalek RD, Pitiyage GN, de Castro AM, Vignola KS, Jones J, Mohney RP, Karoly ED, Prime SS, Parkinson EK. Senescent human fibroblasts show increased glycolysis and redox homeostasis with extracellular metabolomes that overlap with those of irreparable DNA damage, aging, and disease. J Proteome Res. 2015; 14:1854–71. 10.1021/pr501221g 25690941

[41] Rivera-Torres J, Acín-Perez R, Cabezas-Sánchez P, Osorio FG, Gonzalez-Gómez C, Megias D, Cámara C, López-Otín C, Enríquez JA, Luque-García JL, Andrés V. Identification of mitochondrial dysfunction in Hutchinson-Gilford progeria syndrome through use of stable isotope labeling with amino acids in cell culture. J Proteomics. 2013; 91:466–77. 10.1016/j.jprot.2013.08.008 23969228

[42] Bratic A, Larsson NG. The role of mitochondria in aging. J Clin Invest. 2013; 123:951–7. 10.1172/JCI64125 23454757 PMC3582127

[43] Korolchuk VI, Miwa S, Carroll B, von Zglinicki T. Mitochondria in Cell Senescence: Is Mitophagy the Weakest Link? EBioMedicine. 2017; 21:7–13. 10.1016/j.ebiom.2017.03.020 28330601 PMC5514379

[44] Leveille CF, Mikhaeil JS, Turner KD, Silvera S, Wilkinson J, Fajardo VA. Mitochondrial cristae density: a dynamic entity that is critical for energy production and metabolic power in skeletal muscle. J Physiol. 2017; 595:2779–80. 10.1113/JP274158 28217967 PMC5407988

[45] Hessenberger M, Zerbes RM, Rampelt H, Kunz S, Xavier AH, Purfürst B, Lilie H, Pfanner N, van der Laan M, Daumke O. Regulated membrane remodeling by Mic60 controls formation of mitochondrial crista junctions. Nat Commun. 2017; 8:15258. 10.1038/ncomms15258 28561061 PMC5460017

[46] Blum TB, Hahn A, Meier T, Davies KM, Kühlbrandt W. Dimers of mitochondrial ATP synthase induce membrane curvature and self-assemble into rows. Proc Natl Acad Sci U S A. 2019; 116:4250–55. 10.1073/pnas.1816556116 30760595 PMC6410833

[47] Khraiwesh H, López-Domínguez JA, Fernández del Río L, Gutierrez-Casado E, López-Lluch G, Navas P, de Cabo R, Ramsey JJ, Burón MI, Villalba JM, González-Reyes JA. Mitochondrial ultrastructure and markers of dynamics in hepatocytes from aged, calorie restricted mice fed with different dietary fats. Exp Gerontol. 2014; 56:77–88. 10.1016/j.exger.2014.03.023 24704714 PMC4104696

[48] Gollihue JL, Rabchevsky AG. Prospects for therapeutic mitochondrial transplantation. Mitochondrion. 2017; 35:70–9. 10.1016/j.mito.2017.05.007 28533168 PMC5518605

[49] Borgoni S, Kudryashova KS, Burka K, de Magalhães JP. Targeting immune dysfunction in aging. Ageing Res Rev. 2021; 70:101410. 10.1016/j.arr.2021.101410 34280555

[50] Fairlie-Clarke K, Barbour M, Wilson C, Hridi SU, Allan D, Jiang HR. Expression and Function of IL-33/ST2 Axis in the Central Nervous System Under Normal and Diseased Conditions. Front Immunol. 2018; 9:2596. 10.3389/fimmu.2018.02596 30515150 PMC6255965

[51] Loeser RF, Olex AL, McNulty MA, Carlson CS, Callahan MF, Ferguson CM, Chou J, Leng X, Fetrow JS. Microarray analysis reveals age-related differences in gene expression during the development of osteoarthritis in mice. Arthritis Rheum. 2012; 64:705–17. 10.1002/art.33388 21972019 PMC3269534

[52] Cao K, Liao X, Lu J, Yao S, Wu F, Zhu X, Shi D, Wen S, Liu L, Zhou H. IL-33/ST2 plays a critical role in endothelial cell activation and microglia-mediated neuroinflammation modulation. J Neuroinflammation. 2018; 15:136. 10.1186/s12974-018-1169-6 29728120 PMC5935936

[53] Vainchtein ID, Chin G, Cho FS, Kelley KW, Miller JG, Chien EC, Liddelow SA, Nguyen PT, Nakao-Inoue H, Dorman LC, Akil O, Joshita S, Barres BA, et al. Astrocyte-derived interleukin-33 promotes microglial synapse engulfment and neural circuit development. Science. 2018; 359:1269–73. 10.1126/science.aal3589 29420261 PMC6070131

[54] Chung JH, Choi HJ, Kim SY, Hong KS, Min SK, Nam MH, Kim CW, Koh YH, Seo JB. Proteomic and biochemical analyses reveal the activation of unfolded protein response, ERK-1/2 and ribosomal protein S6 signaling in experimental autoimmune myocarditis rat model. BMC Genomics. 2011; 12:520. 10.1186/1471-2164-12-520 22014063 PMC3209477

[55] Seidel SA, Dijkman PM, Lea WA, van den Bogaart G, Jerabek-Willemsen M, Lazic A, Joseph JS, Srinivasan P, Baaske P, Simeonov A, Katritch I, Melo FA, Ladbury JE, et al. Microscale thermophoresis quantifies biomolecular interactions under previously challenging conditions. Methods. 2013; 59:301–15. 10.1016/j.ymeth.2012.12.005 23270813 PMC3644557

[56] Wei JD, Kim JY, Kim AK, Jang SK, Kim JH. RanBPM protein acts as a negative regulator of BLT2 receptor to attenuate BLT2-mediated cell motility. J Biol Chem. 2013; 288:26753–63. 10.1074/jbc.M113.470260 23928309 PMC3772221

[57] Hwang SY, Kuk MU, Kim JW, Lee YH, Lee YS, Choy HE, Park SC, Park JT. ATM mediated-p53 signaling pathway forms a novel axis for senescence control. Mitochondrion. 2020; 55:54–63. 10.1016/j.mito.2020.09.002 32949791

[58] Kang HT, Hwang ES. Nicotinamide enhances mitochondria quality through autophagy activation in human cells. Aging Cell. 2009; 8:426–38. 10.1111/j.1474-9726.2009.00487.x 19473119

