# Supplementary material for: Targeting regulation of ATP synthase 5 alpha/beta dimerization alleviates senescence
Source: Aging (Albany NY). 2022 Jan 30;14(2):678–707. doi: 10.18632/aging.203858 (PMC8833107; doi:10.18632/aging.203858)
Supplement: Supplementary Methods [file aging-14-203858-s001.pdf]

SUPPLEMENTARY METHODS

Synthesis of KB1541 and biotinylated KB1541

General

All chemicals and solvents used in the reaction were purchased from Sigma-Aldrich, TCI, and Acros and were used without further purification. Reaction progress was monitored by TLC on pre-coated silica gel plates with silica gel $60F_{254}$ (Merck; Darmstadt, Germany) and visualized by UV254 light and/or KMnO4 staining for detection purposes. Column chromatography was performed on silica gel (Silica gel 60; 230–400 mesh ASTM, Merck, Darmstadt, Germany). Nuclear magnetic resonance (NMR) spectra were recorded at room temperature and 50°C on either a Bruker BioSpin Advance 300 MHz NMR (^{1}H, 300 MHz; ^{13}C, 75 MHz) or a Bruker Ultrashield 600 MHz Plus (^{1}H, 600 MHz; ^{13}C, 150 MHz) spectrometer. All chemical shifts are reported in parts per million (ppm) from tetramethylsilane (δ = 0) and were measured relative to the solvent in which the sample was analyzed ($CDCl_3$: δ 7.26 for ^{1}H NMR, δ 77.0 for ^{13}C NMR; MeOH-d_4: δ 3.31 for ^{1}H NMR, δ 49.0 for ^{13}C NMR). The ^{1}H NMR shift values are reported as chemical shift (δ), the corresponding integral, multiplicity (s = singlet, br = broad, d = doublet, t = triplet, q = quartet, m = multiplet, dd = doublet of doublets, td = triplet of doublets, qd = quartet of doublets), coupling constant (J in Hz) and assignments. High-resolution mass spectra (HRMS) were recorded on an Agilent 6530 Accurate Mass Q-TOF LC/MS spectrometer. The purity of all final compounds was measured by analytical reverse-phase HPLC on an Agilent 1260 Infinity (Agilent) with a C18 column (Phenomenex, 150 mm × 4.6 mm, 3 µm, 110Å). RP-HPLC was performed using the following isocratic conditions: for method A, mobile phase was acetonitrile and water (48:52, v/v); for method B, mobile phase was acetonitrile and water (30:70, v/v); for method C, mobile phase was methanol and water (35:65, v/v). All compounds were eluted with a flow rate of 1 mL/min and monitored at UV detector (220 nm). The purity of the tested compounds was >97%.

Synthesis

Ethyl 2-chlorooxazole-4-carboxylate (1)

Ethyl 2-aminooxazole-4-carboxylate (468 mg, 3 mmol) was added in portions to a solution of *tert*-butyl nitrite (540 µl, 0.45 mmol) and copper (II) chloride (600 mg, 4.5 mmol) in acetonitrile (22 mL) at 60°C. The mixture was then stirred at 80°C for 1 h. The mixture was cooled and partitioned between dichloromethane, ice, and concentrated hydrochloric acid. The aqueous layer was further extracted with dichloromethane and the combined organics washed with brine, dried ($MgSO_4$), and evaporated. The crude products were purified by column chromatography on silica gel (eluting with hexane/Et_2O = 7:1 to 4:1, v/v) to afford pure compound 1 as a fluffy white solid (338 mg, 64%). R_f = 0.38 (hexane/Et_2O = 2:1, v/v). ^{1}H NMR (300 MHz, $CDCl_3$) δ 8.20 (s, 1H), 4.40 (q, J = 7.2 Hz, 2H), 1.39 (t, J = 6.9 Hz, 3H). LRMS (ESI) m/z 176.1 $[M+H]^+$. All spectroscopic data were in complete agreement with those reported previously[1].

Ethyl 2-(4-(trifluoromethyl)phenyl)oxazole-4-carboxylate (2)

The ethyl 2-Chlorooxazole-4-carboxylate 1 (258 mg, 1.47 mmol), 4-(trifluoromethyl)phenylboronic acid (342 mg, 1.8 mmol, 1.2 eq), and tetrakis(triphenylphosphine) palladium (0) (81 mg, 0.07 mmol, 0.05 eq) were dissolved in toluene (20 mL) and 2 M potassium carbonate solution (2.0 mL, 4.0 mmol) at room temperature under a nitrogen atmosphere. The reaction mixture was stirred under reflux for 1 h. After being cooled at room temperature, the reaction mixture and partitioned between ethyl acetate and 2 M sodium hydroxide solution. The aqueous layer was further washed with ethyl acetate twice. The combined organic layer was washed with brine, dried ($MgSO_4$), and concentrated *in vacuo*. The crude products were purified by column chromatography on silica gel (eluting with hexane/Et_2O = 5:1 to 3:1, v/v) to afford pure compound 2 as a fluffy white solid (268 mg, 64%). R_f = 0.38 (hexane/Et_2O = 2:1, v/v). ^{1}H NMR (300 MHz, $CDCl_3$) δ 8.32 (s, 1H), 8.24 (d, J = 8.1 Hz, 2H), 7.74 (d, J = 8.1 Hz, 2H), 4.45 (q, J = 7.2 Hz, 2H), 1.42 (t, J = 7.2 Hz, 3H). LRMS (ESI) m/z 286.0 $[M+H]^+$ and 308.1 $[M+Na]^+$. All spectroscopic data are in complete agreement with those reported[2].

Ethyl 5-(2-nitrophenyl)-2-(4-(trifluoromethyl)phenyl) oxazole-4-carboxylate (3)

A mixture of 2 (228 mg, 0.8 mmol), 2-iodonitrobenzene (398 mg, 1.6 mmol, 2.0 eq), palladium acetate (11.2 mg, 0.05 mmol, 0.06 eq), triphenyl phosphine (21 mg, 0.08 mmol, 0.1 eq), cesium carbonate (651.6 mg, 2.0 mmol, 2.5 eq), and DMF (4 mL) was flushed with nitrogen and heated at 140°C for 3 h. The cooled mixture was diluted with ethyl acetate and washed with water, brine, dried ($MgSO_4$), and concentrated under reduced pressure. The crude products were purified by column chromatography on silica gel (eluting with hexane/ Et_2O = 5:1 to 1:1, v/v) to afford pure compound 3 as a white needlelike crystal (143 mg, 44%). R_f = 0.20

(hexane/Et_2O = 1:1, v/v). ^1H NMR (300 MHz, $CDCl_3$) δ 8.24 (d, J = 8.1 Hz, 2H), 8.21 (d, J = 10.5 Hz, 1H), 7.86–7.68 (m, 5H), 4.32 (q, J = 7.2 Hz, 2H), 1.27 (t, J = 7.2 Hz, 3H); ^{13}C NMR (75 MHz, $CDCl_3$) δ 166.1, 159.9, 151.7, 148.5, 133.0, 132.6, 131.5, 130.5, 129.3, 127.3, 126.0, 125.9, 124.9, 122.4, 61.8, 14.0. LRMS (ESI) *m/z* 407.0 $[M+H]^+$, 428.7 $[M+Na]^+$, and 445.3 $[M+K]^+$. HRMS (ESI) *m/z* calculated for $C_{19}H_{14}F_3N_2O_5^+$ $[M+H]^+$: 407.0849; found: 407.0809.

Ethyl 5-(2-aminophenyl)-2-(4-(trifluoromethyl)phenyl) oxazole-4-carboxylate (4)

To a solution of 3 (219 mg, 0.54 mmol) in MeOH (15 mL) was added catalytic amount of 10 wt. % palladium on activated carbon. The mixture was shaken under H_2 gas (50 psi) for 1 h. The reaction mixture was filtered through Celite bed. The volatiles were removed under reduced pressure to give 4 (199 mg, 98%) as a white needlelike crystal. R_f = 0.29 (CH_2Cl_2/MeOH = 20:1, v/v). ^1H NMR (300 MHz, $CDCl_3$) δ 8.25 (d, J = 8.1 Hz, 2H), 7.74 (d, J = 8.1 Hz, 2H), 7.44 (t, J = 7.8 Hz, 1H), 7.31 (t, J = 7.8 Hz, 1H), 6.86 (t, J = 7.5 Hz, 1H), 6.83 (d, J = 7.8 Hz, 1H), 4.40 (q, J = 7.2 Hz, 2H), 4.17 (s, 2H), 1.29 (t, J = 7.2 Hz, 3H); ^{13}C NMR (75 MHz, $CDCl_3$) δ 162.0, 159.2, 155.1, 145.8, 132.0, 131.6, 129.5, 127.1, 126.0, 125.9, 118.2, 116.9, 112.4, 61.7, 14.2. LRMS (ESI) *m/z* 377.1 $[M+H]^+$ and 399.1 $[M+Na]^+$. HRMS (ESI) *m/z* calculated for $C_{19}H_{16}F_3N_2O_3^+$ $[M+H]^+$: 377.1108; found: 377.1094.

2-(4-(Trifluoromethyl)phenyl)oxazolo[4,5-c]quinolin-4(5H)-one (5)

A mixture of 4 (101 mg, 0.27 mmol), DME (7 mL), and 2 M potassium carbonate solution (0.5 mL, 1.0 mmol) was stirred under reflux for 12 h. The solvent was removed under reduced pressure and water added. The white needlelike crystal was collected by filtration, washed with Et_2O, and dried *in vacuo* to give 5 (64 mg, 72%). R_f = 0.38 (CH_2Cl_2/MeOH = 20:1, v/v). ^1H NMR (300 MHz, DMSO-d_6) δ 8.44 (d, J = 8.1 Hz, 2H), 8.08 (d, J = 7.8 Hz, 1H), 8.03 (d, J = 8.4 Hz, 2H), 7.63 (t, J = 7.2 Hz, 1H), 7.53 (d, J = 8.1 Hz, 1H), 7.38 (t, J = 7.5 Hz, 1H). LRMS (ESI) *m/z* 353.5 $[M+Na]^+$ and 369.0 $[M+K]^+$. HRMS (ESI) *m/z* calculated for $C_{17}H_{10}F_3N_2O_2^+$ $[M+H]^+$: 331.0689; found: 331.0682.

4-Chloro-2-(4-(trifluoromethyl)phenyl)oxazolo[4,5-c] quinoline (6)

A flame-dried, 100 mL two-necked round-bottomed flask, equipped with magnetic stir bar and charged with compound 5 (296 mg, 0.90 mmol) in dry toluene (15 mL). Phosphorus oxychloride (835 μl, 8.96 mmol) was then added and the reaction mixture was refluxed under argon for 4 h. The progress of the reaction was monitored with TLC (hexane/Et_2O = 1:1, v/v). After the reaction was cooled to room temperature, it was carefully poured into ice brine and basified with aqueous NH_4OH. The reaction mixture was extracted with EtOAc, dried over $MgSO_4$, and concentrated *in vacuo*. The crude product was purified by column chromatography on silica gel (eluting with hexane/Et_2O = 2:1, v/v) to afford product 6 (263 mg, 84%) as a white needlelike crystal. R_f = 0.75 (hexane/Et_2O = 1:1, v/v). ^1H NMR (300 MHz, $CDCl_3$) δ 8.50 (d, J = 8.1 Hz, 2H), 8.29 (d, J = 8.1 Hz, 1H), 8.21 (d, J = 8.1 Hz, 1H), 8.21 (d, J = 8.1 Hz, 1H), 7.86 (d, J = 8.1 Hz, 2H), 7.84–7.69 (m, 2H); ^{13}C NMR (150 MHz, $CDCl_3$) δ 161.9, 153.1, 145.4, 142.5, 133.9, 133.6, 133.5, 130.3, 129.4, 129.3, 128.2, 127.9, 126.3, 126.2, 126.2, 126.1, 124.5, 122.7, 120.5, 115.7. LRMS (ESI) *m/z* 349.0 $[M+H]^+$.

4-(Pyrrolidin-1-yl)-2-(4-(trifluoromethyl)phenyl)oxazolo [4,5-c]quinoline (7, KB1541)

A stirred mixture of the compound 6 (36 mg, 0.10 mmol) and pyrrolidine (345 μl, 4.13 mmol) was heated to 80°C for 3 h under an argon atmosphere. Completion of the reaction was monitored with TLC, as appropriate. After the reaction was complete, excess pyrrolidine was evaporated *in vacuo* if possible. The crude product was purified by column chromatography on silica gel (eluting with hexane/Et_2O = 9:1 to 5:1, v/v) as a yellow solid (26 mg, 68%). R_f = 0.35 (hexane/Et_2O = 3:1, v/v). ^1H NMR (300 MHz, $CDCl_3$) δ 8.37 (d, J = 8.1 Hz, 2H), 8.01 (dd, J = 1.2 and 8.0 Hz, 1H), 7.79 (d, J = 8.4 Hz, 3H), 7.55 (t, J = 6.9 Hz, 1H), 7.28 (t, J = 7.8 Hz, 1H), 4.08 (brs, 4H), 2.08 (t, J = 6.6 Hz, 4H); ^{13}C NMR (150 MHz, $CDCl_3$) δ 158.7, 1535., 149.5, 146.9, 132.5, 132.3, 130.4, 129.3, 127.2, 126.5, 126.4, 125.9, 125.9, 125.9, 125.8, 124.7, 122.9, 121.7, 120.1, 112.2, 48.3, 25.5. LRMS (ESI) *m/z* 384.3 $[M+H]^+$. HRMS (ESI) *m/z* calculated for $C_{21}H_{17}F_3N_3O^+$ $[M+H]^+$: 384.1318; found: 384.1307. >98% purity as determined by RP-HPLC, method D, t_R = 7.932 min.

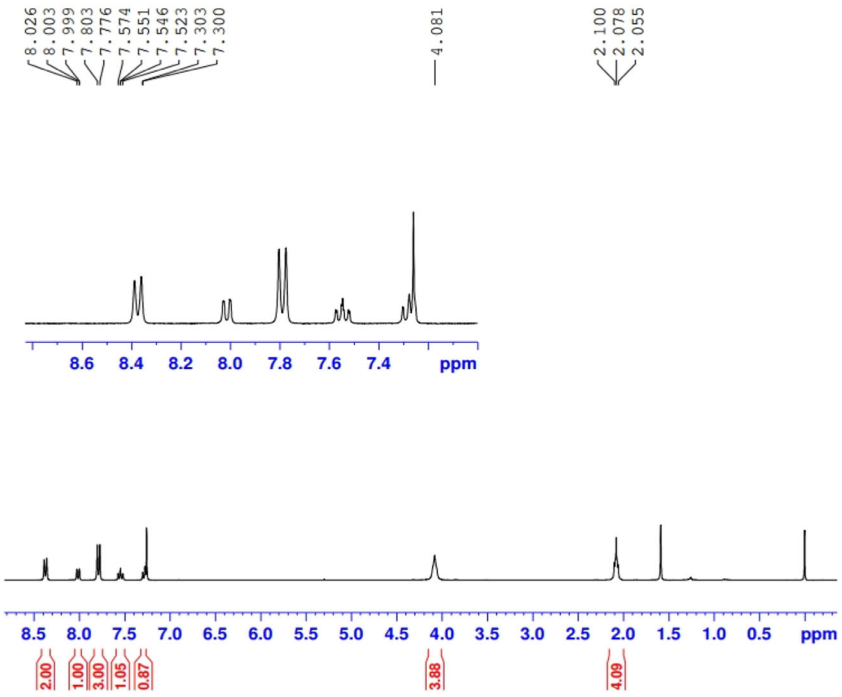

^{1}H NMR spectra of KB1541 measure in $CDCl_3$ at 300 MHz.

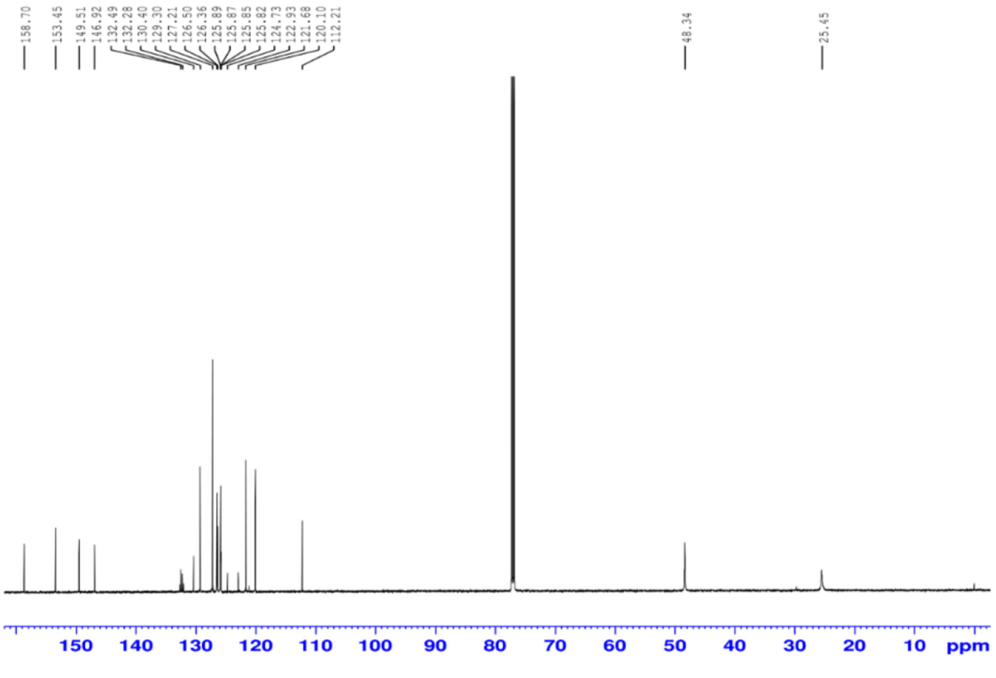

^{13}C NMR spectra of KB-1541 measure in $CDCl_3$ at 75 MHz.

tert-Butyl (pyrrolidine-3-ylmethyl)carbamate (12)

To a stirred solution of tert-Butyl ((1-benzylpyrrolidin-3-yl)methyl)carbamate (11) (200 mg, 0.69 mmol) in methanol (3 mL) was added 10 wt. % palladium on activated carbon (20 mg) and catalytic amount of acetic acid. The solution was then stirred in an atmosphere of H_2 gas for 8 h. The reaction mixture was filtered through a celite pad and concentrated under reduced pressure. The crude residue was used in the next step without further purification. R_f = 0.07 (CH_2Cl_2/MeOH = 10:1, v/v).

tert-Butyl ((1-(2-(4-(trifluoromethyl)phenyl)oxazolo[4,5-c]quinolin-4-yl)pyrrolidin-3-yl)methyl)carbamate (13)

4-Chloro-2-(4-(trifluoromethyl)phenyl)oxazolo[4,5-c]quinoline (12) (88.7 mg, 0.25 mmol) and tert-Butyl (pyrrolidin-3-ylmethyl)carbamate (6) (300 mg, 1.50 mmol) were placed in an oven dried 100 mL two-necked round bottom flask that was then fitted with a rubber septum and a three-way connected to a balloon filled with argon. The flask was flushed with argon and anhydrous THF (5 mL) was added, followed by triethylamine (400 µl, 2.87 mmol). The reaction mixture was stirred at 60°C for 19 h. The resulting reaction mixture was cooled to room temperature and the solvent was evaporated in vacuo. The crude residue was purified by silica gel column chromatography (CH_2Cl_2/MeOH, 160:1 to 60:1, v/v) to afford compound 13 (122 mg, 95%) as a dark green solid. R_f = 0.50 (CH_2Cl_2/MeOH = 20:1, v/v). ^{1}H NMR (600 MHz, MeOD) δ 8.28 (d, J = 7.8 Hz, 2H), 7.90 (d, J = 7.8 Hz, 1H), 7.83 (d, J = 7.8 Hz, 2H), 7.68 (d, J = 8.4 Hz, 1H), 7.51 (t, J = 7.2 Hz, 2H), 7.26 (t, J = 7.2 Hz, 2H), 4.07 (brs, 2H), 3.83 (brs, 1H), 3.62 (brs, 1H), 3.20 (d, J = 7.2 Hz, 2H), 2.59–2.43 (m, 1H), 2.19–2.11 (m, 1H), 1.83–1.72 (m, 1H), 1.48 (s, 9H); ^{13}C NMR (150 MHz, $CDCl_3$) δ 158.95, 156.04, 153.58, 149.46, 146.79, 132.64, 132.43, 130.37, 129.43, 127.35, 126.57, 126.36, 125.94, 124.71, 121.98, 120.17, 112.35, 79.46, 71.88, 62.80, 58.43, 57.63, 55.35, 51.62, 47.86, 46.54, 43.24, 39.16, 28.43. HRMS m/z calculated for $C_{27}H_{27}F_3N_4O_3$ $[M+H]^+$: 513.2108; found: 513.2086. >95% purity (as determined by RP-HPLC, method A, t_R = 6.704 min).

(1-(2-(4-(Trifluoromethyl)phenyl)oxazolo[4,5-c]quinolin-4-yl)pyrrolidin-3-yl)methanamine (14)

The compound 13 (122 mg, 0.24 mmol) was dissolved in anhydrous CH_2Cl_2 (6 mL) in a 100 mL two-necked round bottom flask containing a magnetic stir bar and purged with argon gas. The reaction vessel was maintained in an ice-water bath, and trifluoroacetic acid (2.5 mL) was added slowly dropwise. The ice-water bath was removed after 30 min, and the reaction mixture was stirred at room temperature for 1 h. The solvent was removed and concentrated under reduced pressure to afford the corresponding crude product (109 mg, 86%) 14 as a yellow oil, which was used in the next step without further purification. R_f = 0.08 (CH_2Cl_2/MeOH = 10:1, v/v). ^{1}H NMR (600 MHz, MeOD) δ 8.53 (d, J = 8.4 Hz, 2H), 8.31 (d, J = 8.4 Hz, 1H), 8.13 (d, J = 9.0 Hz, 1H), 7.96 (d, J = 7.8 Hz, 2H), 7.87 (t, J = 8.4 Hz, 1H), 7.69 (t, J = 7.8 Hz, 1H), 4.24 (brs, 1H), 4.09 (brs, 1H), 3.37–3.26 (m, 3H), 3.25–3.18 (m, 1H), 2.98–2.87 (m, 1H), 2.58–2.50 (m, 1H), 2.16–2.07 (m, 1H); ^{13}C NMR (150 MHz, MeOD) δ 161.59, 161.23, 154.72, 145.80, 136.60, 133.70, 133.05, 128.59, 127.91, 126.41, 125.99, 125.52, 124.61, 118.33, 118.20, 113.69, 110.77, 54.78, 52.90, 49.04, 37.41, 31.64. HRMS m/z calculated for $C_{22}H_{19}F_3N_4O$ $[M+H]^+$: 413.1584; found: 413.1590. >95% purity (as determined by RP-HPLC, method B, t_R = 6.330 min).

5-((3aS,4S,6aR)-2-Oxohexahydro-1H-thieno[3,4-d]imidazol-4-yl)-N-((1-(2-(4-(trifluoromethyl)phenyl)oxazolo[4,5-c]quinolin-4-yl)pyrrolidin-3-yl)methyl)pentanamide (15, Biotinylated KB1541)

The compound 14 (50.0 mg, 0.12 mmol) and *N*-succinimidyl D-biotinate (82 mg, 0.24 mmol, 2.0 eq.) were placed in an oven dried 100 mL two-necked round bottom flask that was then fitted with a rubber septum and a three-way connected to a balloon filled with argon. The flask was flushed with argon and anhydrous DMF (5 mL) was added, followed by triethylamine (84 µl, 0.6 mmol, 5.0 eq.). The reaction mixture was stirred at room temperature for 12 h. TLC (CH_2Cl_2: MeOH = 8:1, v/v) showed a complete conversion, the solvent was co-evaporated with toluene. The crude product was purified by silica gel column chromatography (CH_2Cl_2/MeOH = 50:1 to 6:1, v/v) to afford compound 15 (41.0 mg, 53%) as a green solid. R_f = 0.55 (CH_2Cl_2/MeOH = 8:1, v/v). ^{1}H NMR (600 MHz, MeOD) δ 8.39 (d, J = 7.8 Hz, 2H), 8.00 (d, J = 7.8 Hz, 1H), 7.87 (d, J = 7.8 Hz, 2H), 7.74 (d, J = 8.4 Hz, 1H), 7.54 (t, J = 7.2 Hz, 1H), 7.29 (t, J = 7.2 Hz, 1H), 4.42–4.35 (m, 1H), 4.25–4.13 (m, 3H), 3.99–3.91 (m, 1H), 3.79–3.71 (m, 1H), 3.44–3.28 (m, 2H), 3.17–3.09 (m, 1H), 2.86–2.78 (m, 1H), 2.67–2.55 (m, 2H), 2.31–2.23 (m, 2H), 2.22–2.15 (m, 1H), 1.89–1.80 (m, 1H), 1.77–1.62 (m, 3H), 1.61–1.53 (m, 1H), 1.50–1.41 (m, 2H); ^{13}C NMR (150 MHz, $CDCl_3$) δ 174.85, 164.59, 159.10, 153.50, 149.54, 146.61, 132.46, 130.29, 129.18, 127.24, 126.08, 125.73, 125.69, 124.84, 121.86, 119.76, 112.08, 61.97, 60.23, 55.48, 51.62, 41.39, 41.33, 39.52, 38.59, 35.46, 28.63, 28.31, 28.07, 25.47. HRMS m/z calculated for $C_{32}H_{33}F_3N_6O_3S$ $[M+H]^+$: 639.2360; found: 639.2333. >95% purity (as determined by RP-HPLC, method C, t_R = 9.499 min).

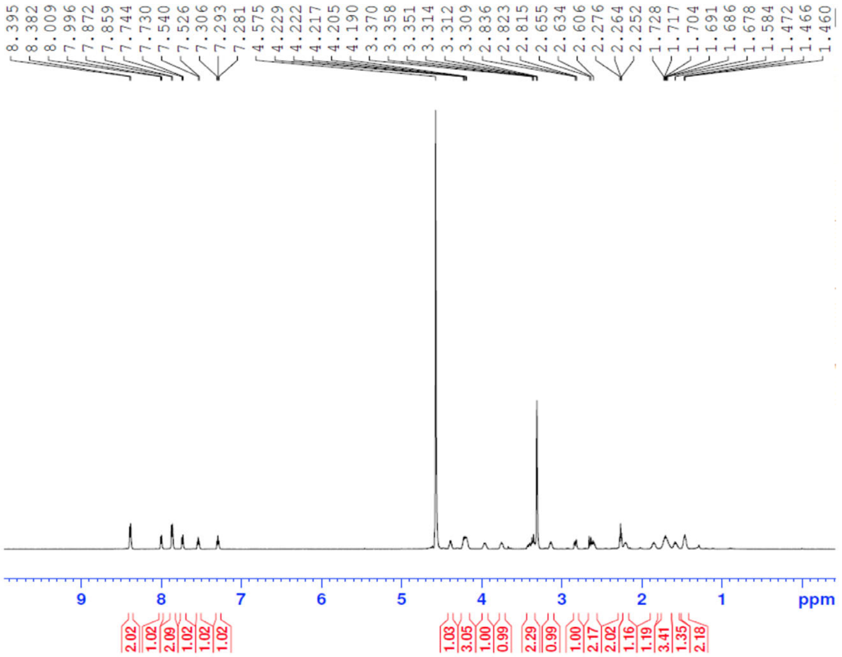

^{1}H NMR spectra of biotinylated KB1541 measure in MeOD at 600 MHz.

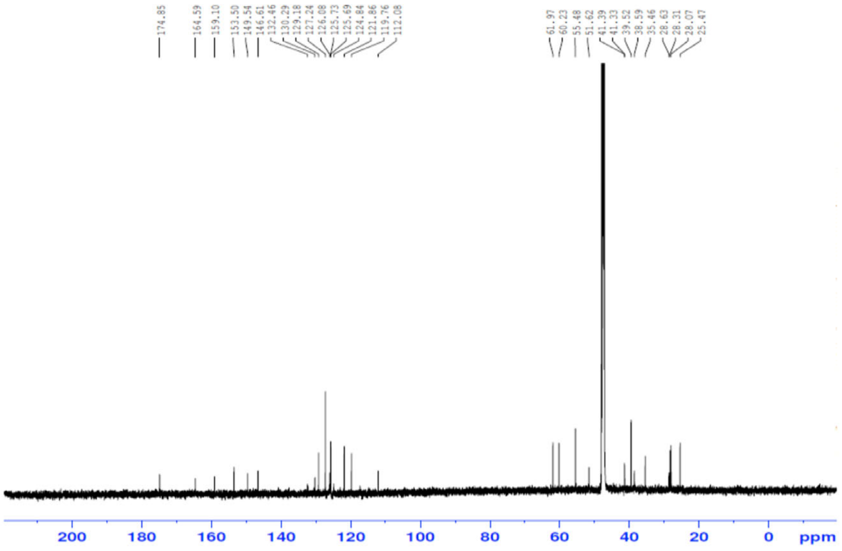

^{13}C NMR spectra of biotinylated KB1541 in MeOD at 150 MHz.
