# Supplementary material for: Targeting regulation of ATP synthase 5 alpha/beta dimerization alleviates senescence
Source: Aging (Albany NY). 2022 Jan 30;14(2):678–707. doi: 10.18632/aging.203858 (PMC8833107; doi:10.18632/aging.203858)
Supplement: Supplementary Figures [file aging-14-203858-s002.pdf]

SUPPLEMENTARY FIGURES

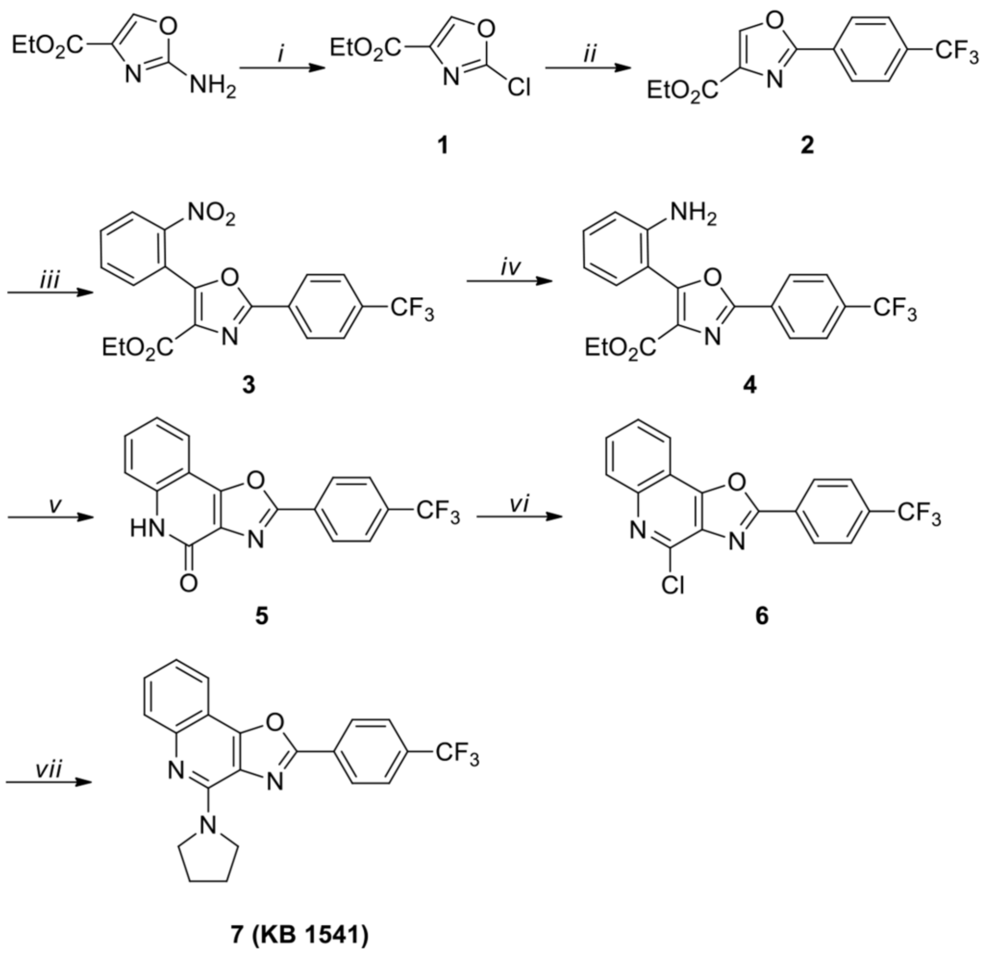

Supplementary Figure 1. Synthesis of KB 1541. Compound 1 was synthesized from commercially available ethyl 2-aminooxazole-4-carboxylate by treating tert-butyl nitrile and copper (II) chloride in acetonitrile at 80°C for 2 h in 64% yield. Compound 2 was obtained by reacting compound 1 with 4-(trifluoromethyl)phenylboronic acid, tetrakis(triphenylphosphine)palladium(0) and 2 M potassium carbonate solution in toluene at 80°C for 1 h in 64% yield. Compound 3 was obtained by reacting 2 with 2-iodonitrobenzene, palladium acetate, triphenyl phosphine, and cesium carbonate in toluene at 90°C for 3 h in 44% yield. The nitro group of compound 3 was reduced with catalytic amount of 10 wt. % palladium on activated carbon in methanol to provide compound 4. The mixture was shaken under hydrogen gas (50 psi) at room temperature for 1 h in 98% yield. Intramolecular cyclization of compound 4 was accomplished with ethylene glycol dimethyl ether (DME) and 2 M potassium carbonate solution at 90°C for 12 h to afford compound 5 in 72% yield. Compound 6 was obtained by reacting compound 5 with phosphorus oxychloride in toluene at 120°C for 4 h in 84% yield. Compound 7 (KB 1541) was obtained by reacting 6 with pyrrolidine at 80°C for 3 h in 68% yield. Briefly, a total of 7 steps of reaction were carried out using ethyl 2-aminooxazole-4-carboxylate purchased from a commercial source. In order, they are Sandmeyer reaction, Suzuki reaction, Heck reaction, Hydrogenation, Cyclization, Chlorination and Alkylation.

Reagents and Conditions: (i) *t*-BuONO,$CuCl_2$, acetonitrile, 80°C, 2 h; (ii) *p*-$CF_3PhB(OH)_2$, $Pd(PPh_3)_4$, toluene, H_2O, K_2CO_3, 80°C, 1 h; (iii) 2-nitroiodobenzene, $Pd(OAc)_2$, $P(o\text{-}tol)_3$, Cs_2CO_3, toluene, 90°C, 3 h; (iv) 10% Pd/C, H_2, MeOH, rt; (v) 2 M K_2CO_3, DME, 90°C, 12 h; (vi) $POCl_3$, toluene, 120°C, 4 h; (vii) Pyrrolidine, 80°C, 3 h.

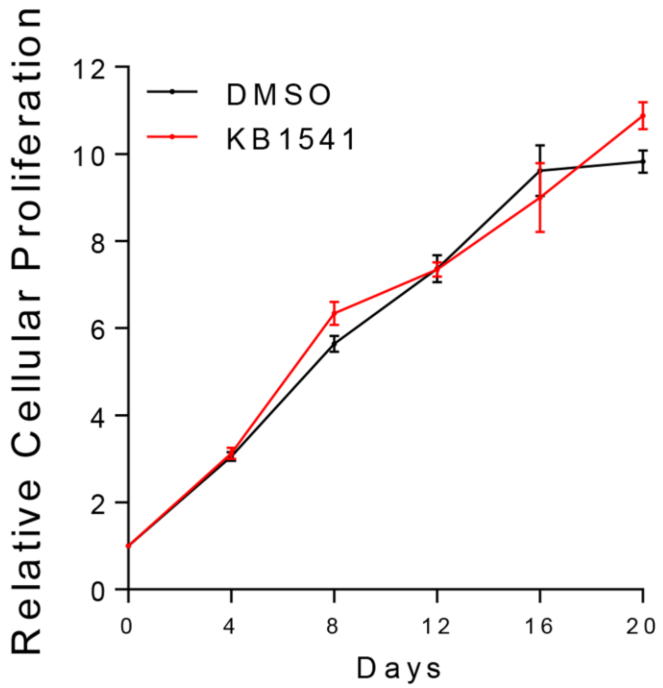

Supplementary Figure 2. Effect of KB1541 on the proliferation of young fibroblasts. Cell proliferation of young fibroblasts treated with 4 µM KB1541 was evaluated at different times (0–20 days). Mean ± S.D., *n* = 10.

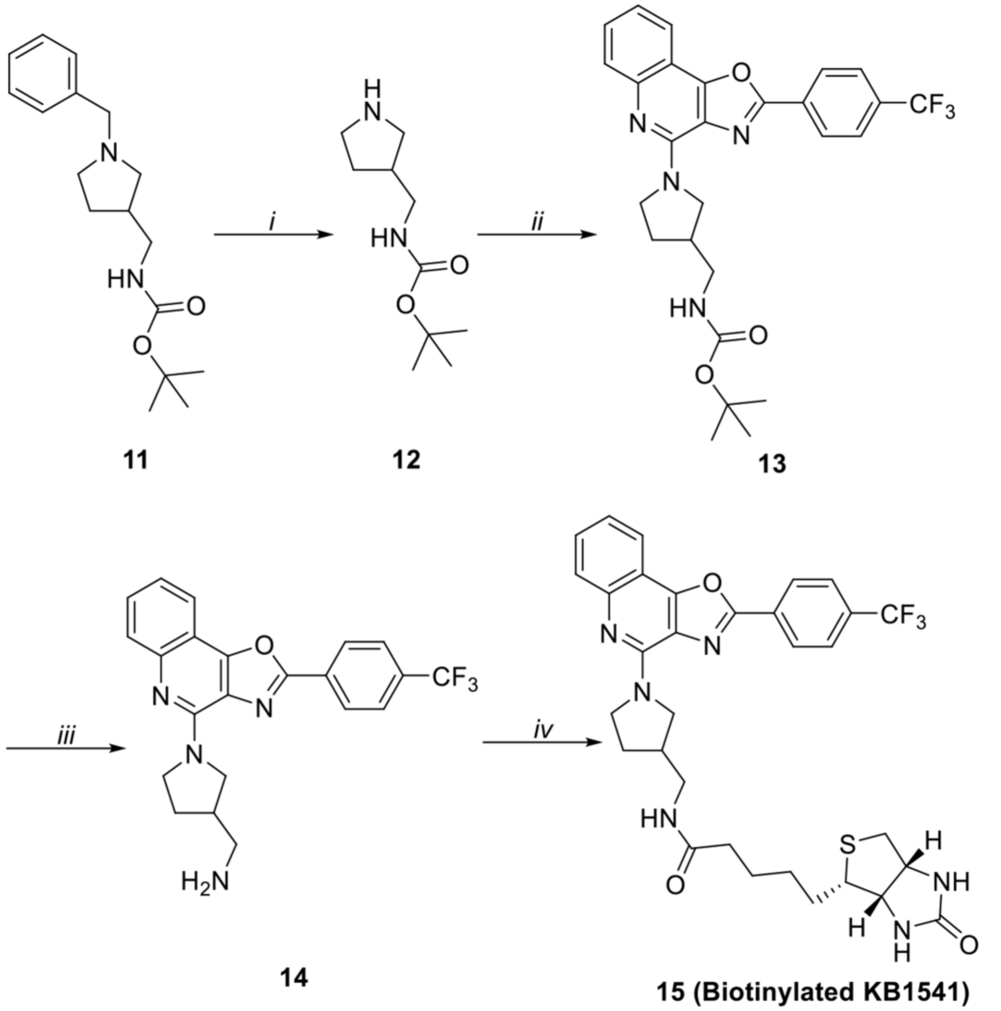

Supplementary Figure 3. Synthesis of biotinylated KB1541. The synthesis of biotinylated KB1541 (compound 12) was not possible using the scheme used in Supplementary Figure 1. Therefore, the synthetic scheme for biotinylated KB1541 (compound 12) was re-established as summarized in Supplementary Figure 2. Compound 9 was obtained through debenzylation by reacting commercially available compound 8 with 10 wt % palladium on activated carbon and catalytic amount of acetic acid in methanol. The mixture was shaken under hydrogen gas (50 psi) at room temperature for 8 h. Compound 10 was obtained by reacting 9 with compound 6 from Scheme 1 and excess amount of triethylamine (TEA) in tetrahydrofuran (THF) at 60°C. Deprotection of Boc group in compound 10 was accomplished by treating trifluoroacetic acid (TFA) in dichloromethane at room temperature for 3 h to afford compound 11. Through this reaction, we were able to obtain a compound in which a linker is conjugated to compound 7. The crude product 11 was used for the final step without further purification. Compound 12 was obtained by reacting 11 with N-succinimidyl D-biotinate, TEA in dimethylformamide (DMF) at room temperature in 53% yield.

Reagents and conditions: (i) Pd/C, H_2, acetic acid, MeOH, rt, 8 h; (ii) compound 6, TEA, THF, 60°C, 19 h; (iii) TFA, CH_2Cl_2, rt, 3 h; (iv) *N*-succinimidyl *D*-biotinate, TEA, DMF, rt, 12 h.

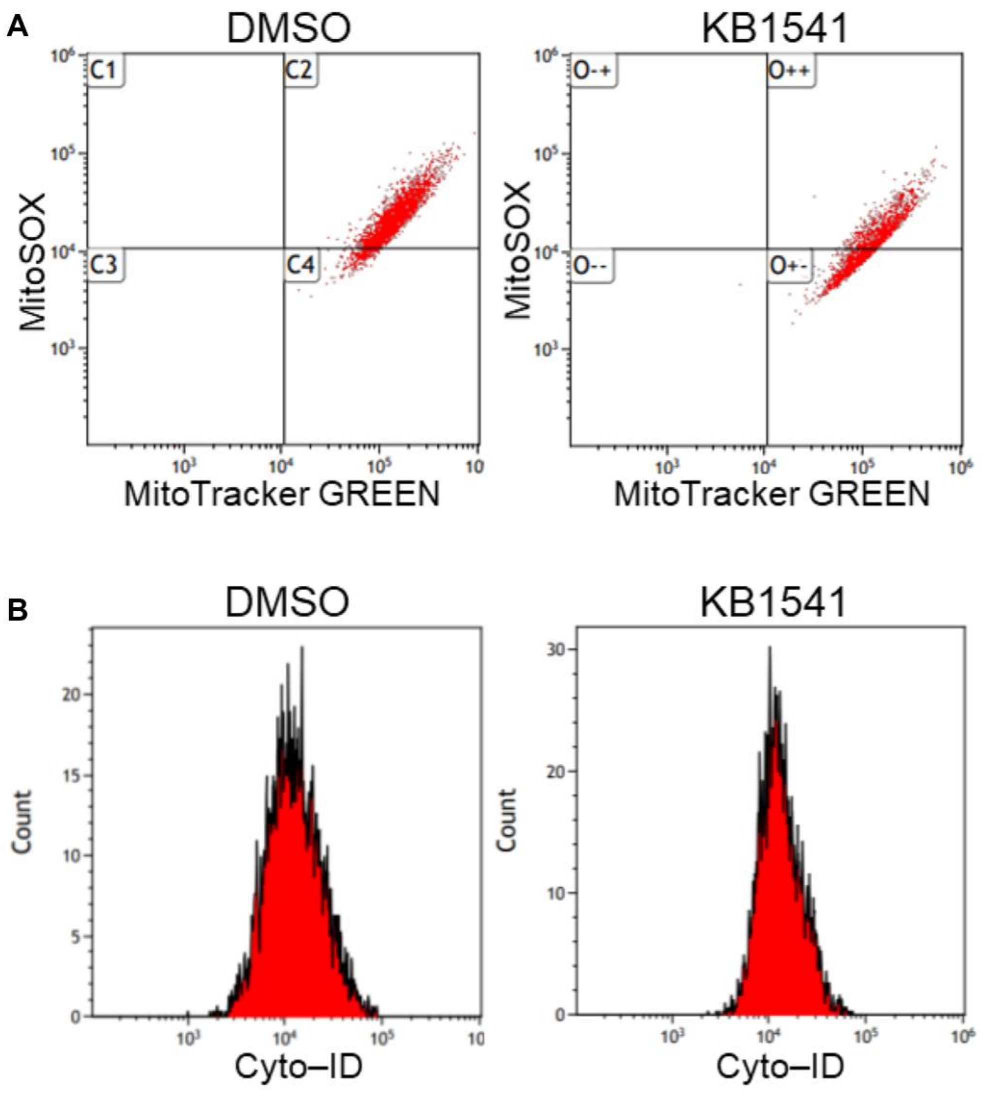

Supplementary Figure 4. Flow cytometry data used to generate graph in Figure 5C, 5D and 5E. (**A**) Flow cytometry data of ROS (Figure 5C) and mitochondrial mass (Figure 5D) using MitoSOX and MitoTracker green, respectively, were presented. $^{**}P < 0.01$, student *t*-test. Mean ± S.D., $n = 3$. (**B**) Flow cytometric data of autophagy level (Figure 5E) using Cyto–ID assay were presented. $^{**}P < 0.01$, student *t*-test. Mean ± S.D., $n = 3$.

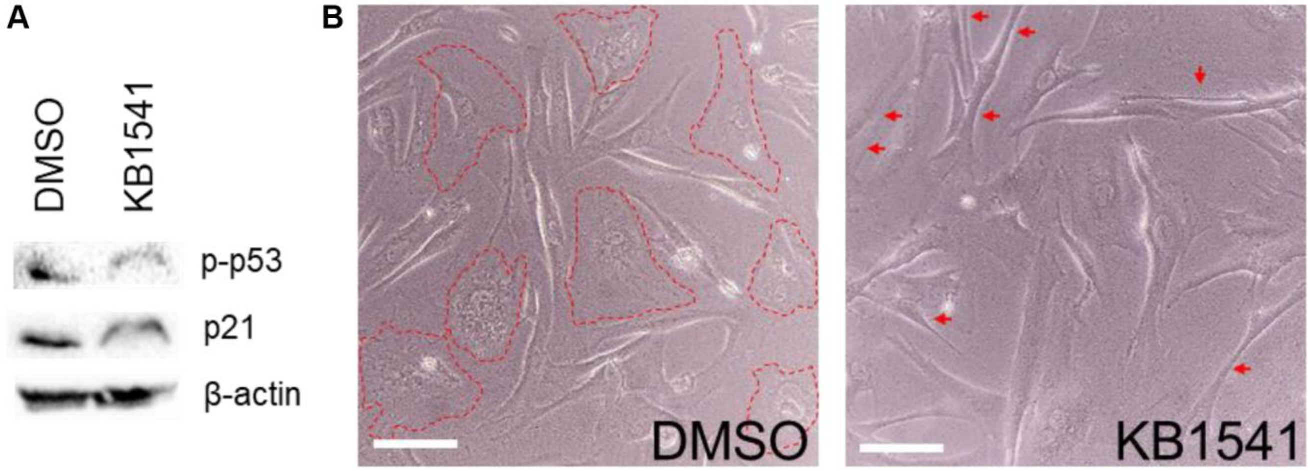

Supplementary Figure 5. KB1541 ameliorates senescence phenotypes. (**A**) Western blot analysis of senescent fibroblasts after treatment with DMSO or KB1541. The primary antibodies included anti-phospho-p53 antibody (sc-377561; 1:500 dilution, Santa Cruz), anti-p21 antibody (sc-6246; 1:500 dilution, Santa Cruz) and HRP–conjugated β–actin (sc47778; 1:1000 dilution; Santa Cruz). (**B**) Morphologies of senescence fibroblasts after treatment with DMSO or KB1541. Senescent fibroblasts treated with DMSO showed a large and flat structure (dotted lines), whereas senescent fibroblasts treated with KB1541 showed a small spindle shape (red arrows). Scale bar 100 µm.
