# Supplementary material for: Targeting regulation of ATP synthase 5 alpha/beta dimerization alleviates senescence
Source: Aging (Albany NY). 2022 Jan 30;14(2):678–707. doi: 10.18632/aging.203858 (PMC8833107; doi:10.18632/aging.203858)
Supplement: Supplementary Tables 1 and 2 [file aging-14-203858-s003.pdf]

SUPPLEMENTARY TABLES

Supplementary Table 1. Reporting parameters for compound screening data.

Category	Parameter	Description
Assay	Type of assay	Cell-based phenotypic assays.
	Target	Cell proliferation
	Primary measurement	Detection of double-stranded DNA using intercalated fluorescence enhancement of fluorophore
	Key reagents	Gel Green nucleic acid gel stain and 0.2% SDS
	Assay protocol	Key steps are outlined in Supplementary Table 2
	Additional comments	
Library	Library size	23 compounds arrayed in 96-well plates as single compounds at 10 mM in DMSO
	Library composition	A unique collection of IL-33 inhibitors
	Source	Drug library for IL-33 inhibitors
	Additional comments	
Screen	Format	96-well plate (353072; BD BioSciences)
	Concentration(s) tested	4 μM concentration, 1:2,500 dilution
	Plate controls	Negative control: No cells (A1-A12)
	Reagent/compound dispensing system	
	Detection instrument and software	VICTOR Multilabel Plate Reader (PerkinElmer, USA)
	Assay validation/QC	
	Correction factors	
	Normalization	(average of six replicates – average of negative control)/ (average of DMSO control – average of negative control)
	Additional comments	
Post-HTS analysis	Hit criteria	The inhibitor that led to the highest increase was considered potential hit.
	Hit rate	1 out of 23 (4.35%)
	Additional assay(s)	
	Confirmation of hit purity and structure	
	Additional comments	

Supplementary Table 2. HTS assay protocol table.

Step	Parameter	Value	Description
1	Plate cells	1,000 cells/well	1,000 senescent fibroblasts
2	Library compounds	200 µl	4 µM concentration, 1:2,500 dilution
3	Incubation time	21 days	37°C
4	Wash cells	200 µl	PBS
5	Cell lysis	50 µl	0.2% SDS
6	Incubation time	2 hr	37°C
7	Staining of double-stranded DNA	150 µl	Diluted Gel Green solution (1:1,1,000 in D.W.)
8	Incubation time	10 min	Gel Green nucleic acid gel stain and
9	Assay readout	480 and 520 nm	VICTOR Multilabel Plate Reader (PerkinElmer, USA)

Step	Notes
1	Senescent fibroblasts were plated in 96-well plates at a density of 1,000 cells per well
2	Components of the IL-33 inhibitor library were diluted to a final concentration of 4 µM in media.
3	Diluted compounds in media was added to wells every 4 days with 12 channel multi pipette.
4	At 21 days after drug treatment, cells were washed twice with phosphate-buffered saline (PBS)
5	Cells were lysed in 50 µl of 0.2% SDS.
6	The plates were incubated at 37°C for 1 hr.
7	Gel Green (150 µl) nucleic acid gel stain (1:1,000 in DW) was added to the wells.
8	The plates were incubated at 37°C for 10 min.
9	(average of six replicates – average of negative control)/(average of DMSO control – average of negative control)
