# Supplementary material for: Targeting regulation of ATP synthase 5 alpha/beta dimerization alleviates senescence
Source: Aging (Albany NY). 2022 Jan 30;14(2):678–707. doi: 10.18632/aging.203858 (PMC8833107; doi:10.18632/aging.203858)
Supplement: Supplementary Table 3 [file aging-14-203858-s004.docx]

Supplementary Table 3. Detailed list of the IM-MS/MS TOF analysis.

| Accession | -10lgP | Avg. Mass | Description |
| --- | --- | --- | --- |
| P08670\|VIME_HUMAN | 703.72 | 53652 | Vimentin OS=Homo sapiens OX=9606 GN=VIM PE=1 SV=4 |
| P60709\|ACTB_HUMAN | 632.83 | 41737 | Actin cytoplasmic 1 OS=Homo sapiens OX=9606 GN=ACTB PE=1 SV=1 |
| P63261\|ACTG_HUMAN | 630.54 | 41793 | Actin cytoplasmic 2 OS=Homo sapiens OX=9606 GN=ACTG1 PE=1 SV=1 |
| Q15149\|PLEC_HUMAN | 602.75 | 531796 | Plectin OS=Homo sapiens OX=9606 GN=PLEC PE=1 SV=3 |
| P35579\|MYH9_HUMAN | 563.77 | 226530 | Myosin-9 OS=Homo sapiens OX=9606 GN=MYH9 PE=1 SV=4 |
| P04264\|K2C1_HUMAN | 550.43 | 66039 | Keratin type II cytoskeletal 1 OS=Homo sapiens OX=9606 GN=KRT1 PE=1 SV=6 |
| P68032\|ACTC_HUMAN | 528.45 | 42019 | Actin alpha cardiac muscle 1 OS=Homo sapiens OX=9606 GN=ACTC1 PE=1 SV=1 |
| P35527\|K1C9_HUMAN | 519.3 | 62064 | Keratin type I cytoskeletal 9 OS=Homo sapiens OX=9606 GN=KRT9 PE=1 SV=3 |
| P21333\|FLNA_HUMAN | 466.15 | 280737 | Filamin-A OS=Homo sapiens OX=9606 GN=FLNA PE=1 SV=4 |
| Q13813\|SPTN1_HUMAN | 452.34 | 284538 | Spectrin alpha chain non-erythrocytic 1 OS=Homo sapiens OX=9606 GN=SPTAN1 PE=1 SV=3 |
| P02545\|LMNA_HUMAN | 449.56 | 74140 | Prelamin-A/C OS=Homo sapiens OX=9606 GN=LMNA PE=1 SV=1 |
| P68104\|EF1A1_HUMAN | 438.63 | 50141 | Elongation factor 1-alpha 1 OS=Homo sapiens OX=9606 GN=EEF1A1 PE=1 SV=1 |
| P05783\|K1C18_HUMAN | 425.31 | 48058 | Keratin type I cytoskeletal 18 OS=Homo sapiens OX=9606 GN=KRT18 PE=1 SV=2 |
| P48681\|NEST_HUMAN | 422.65 | 177438 | Nestin OS=Homo sapiens OX=9606 GN=NES PE=1 SV=2 |
| P13645\|K1C10_HUMAN | 421.34 | 58827 | Keratin type I cytoskeletal 10 OS=Homo sapiens OX=9606 GN=KRT10 PE=1 SV=6 |
| Q8WWI1\|LMO7_HUMAN | 412.78 | 192694 | LIM domain only protein 7 OS=Homo sapiens OX=9606 GN=LMO7 PE=1 SV=3 |
| Q9UHD8\|SEPT9_HUMAN | 408.04 | 65402 | Septin-9 OS=Homo sapiens OX=9606 GN=SEPTIN9 PE=1 SV=2 |
| Q01082\|SPTB2_HUMAN | 401.72 | 274608 | Spectrin beta chain non-erythrocytic 1 OS=Homo sapiens OX=9606 GN=SPTBN1 PE=1 SV=2 |
| Q9UHB6\|LIMA1_HUMAN | 390.8 | 85226 | LIM domain and actin-binding prote in 1 OS=Homo sapiens OX=9606 GN=LIMA1 PE=1 SV=1 |
| P02538\|K2C6A_HUMAN | 383.62 | 60045 | Keratin type II cytoskeletal 6A OS=Homo sapiens OX=9606 GN=KRT6A PE=1 SV=3 |
| P67936\|TPM4_HUMAN | 381.86 | 28522 | Tropomyosin alpha-4 chain OS=Homo sapiens OX=9606 GN=TPM4 PE=1 SV=3 |
| Q05682\|CALD1_HUMAN | 378.03 | 93231 | Caldesmon OS=Homo sapiens OX=9606 GN=CALD1 PE=1 SV=3 |
| Q09666\|AHNK_HUMAN | 376.58 | 629114 | Neuroblast differentiation-associated protein AHNAK OS=Homo sapiens OX=9606 GN=AHNAK PE=1 SV=2 |
| P12814\|ACTN1_HUMAN | 375.19 | 103058 | Alpha-actinin-1 OS=Homo sapiens OX=9606 GN=ACTN1 PE=1 SV=2 |
| P11142\|HSP7C_HUMAN | 374.39 | 70898 | Heat shock cognate 71 kDa protein OS=Homo sapiens OX=9606 GN=HSPA8 PE=1 SV=1 |
| Q14764\|MVP_HUMAN | 370.6 | 99327 | Major vault protein OS=Homo sapiens OX=9606 GN=MVP PE=1 SV=4 |
| P07951\|TPM2_HUMAN | 369.96 | 32851 | Tropomyosin beta chain OS=Homo sapiens OX=9606 GN=TPM2 PE=1 SV=1 |
| P02533\|K1C14_HUMAN | 369.61 | 51562 | Keratin type I cytoskeletal 14 OS=Homo sapiens OX=9606 GN=KRT14 PE=1 SV=4 |
| P09493\|TPM1_HUMAN | 366.53 | 32709 | Tropomyosin alpha-1 chain OS=Homo sapiens OX=9606 GN=TPM1 PE=1 SV=2 |
| P04259\|K2C6B_HUMAN | 362.93 | 60067 | Keratin type II cytoskeletal 6B OS=Homo sapiens OX=9606 GN=KRT6B PE=1 SV=5 |
| P35908\|K22E_HUMAN | 359.72 | 65433 | Keratin type II cytoskeletal 2 epidermal OS=Homo sapiens OX=9606 GN=KRT2 PE=1 SV=2 |
| P08779\|K1C16_HUMAN | 359.35 | 51268 | Keratin type I cytoskeletal 16 OS=Homo sapiens OX=9606 GN=KRT16 PE=1 SV=4 |
| O43707\|ACTN4_HUMAN | 356.85 | 104854 | Alpha-actinin-4 OS=Homo sapiens OX=9606 GN=ACTN4 PE=1 SV=2 |
| Q15019\|SEPT2_HUMAN | 352.96 | 41487 | Septin-2 OS=Homo sapiens OX=9606 GN=SEPTIN2 PE=1 SV=1 |
| P35580\|MYH10_HUMAN | 352.69 | 228997 | Myosin-10 OS=Homo sapiens OX=9606 GN=MYH10 PE=1 SV=3 |
| P22626\|ROA2_HUMAN | 348.74 | 37430 | Heterogeneous nuclear ribonucleoproteins A2/B1 OS=Homo sapiens OX=9606 GN=HNRNPA2B1 PE=1 SV=2 |
| O00159\|MYO1C_HUMAN | 347.02 | 121682 | Unconventional myosin-Ic OS=Homo sapiens OX=9606 GN=MYO1C PE=1 SV=4 |
| P07355\|ANXA2_HUMAN | 346.28 | 38604 | Annexin A2 OS=Homo sapiens OX=9606 GN=ANXA2 PE=1 SV=2 |
| Q562R1\|ACTBL_HUMAN | 345.22 | 42003 | Beta-actin-like protein 2 OS=Homo sapiens OX=9606 GN=ACTBL2 PE=1 SV=2 |
| P13647\|K2C5_HUMAN | 339.87 | 62378 | Keratin type II cytoskeletal 5 OS=Homo sapiens OX=9606 GN=KRT5 PE=1 SV=3 |
| Q9NVA2\|SEP11_HUMAN | 339.66 | 49398 | Septin-11 OS=Homo sapiens OX=9606 GN=SEPTIN11 PE=1 SV=3 |
| Q16352\|AINX_HUMAN | 337.06 | 55391 | Alpha-internexin OS=Homo sapiens OX=9606 GN=INA PE=1 SV=2 |
| P08729\|K2C7_HUMAN | 335.92 | 51386 | Keratin type II cytoskeletal 7 OS=Homo sapiens OX=9606 GN=KRT7 PE=1 SV=5 |
| Q00839\|HNRPU_HUMAN | 334.69 | 90585 | Heterogeneous nuclear ribonucleoprotein U OS=Homo sapiens OX=9606 GN=HNRNPU PE=1 SV=6 |
| P11021\|BIP_HUMAN | 334.38 | 72333 | Endoplasmic reticulum chaperone BiP OS=Homo sapiens OX=9606 GN=HSPA5 PE=1 SV=2 |
| P26599\|PTBP1_HUMAN | 333 | 57221 | Polypyrimidine tract-binding protein 1 OS=Homo sapiens OX=9606 GN=PTBP1 PE=1 SV=1 |
| Q15746\|MYLK_HUMAN | 332.49 | 210713 | Myosin light chain kinase smooth muscle OS=Homo sapiens OX=9606 GN=MYLK PE=1 SV=4 |
| Q16181\|SEPT7_HUMAN | 331.81 | 50680 | Septin-7 OS=Homo sapiens OX=9606 GN=SEPTIN7 PE=1 SV=2 |
| P06576\|ATPB_HUMAN | 331.48 | 56560 | ATP synthase subunit beta mitochondrial OS=Homo sapiens OX=9606 GN=ATP5F1B PE=1 SV=3 |
| P06753\|TPM3_HUMAN | 329.36 | 32950 | Tropomyosin alpha-3 chain OS=Homo sapiens OX=9606 GN=TPM3 PE=1 SV=2 |
| Q9P2E9\|RRBP1_HUMAN | 327.41 | 152456 | Ribosome-binding protein 1 OS=Homo sapiens OX=9606 GN=RRBP1 PE=1 SV=5 |
| P21980\|TGM2_HUMAN | 323.68 | 77329 | Protein-glutamine gamma-glutamyltransferase 2 OS=Homo sapiens OX=9606 GN=TGM2 PE=1 SV=2 |
| P06748\|NPM_HUMAN | 322.73 | 32575 | Nucleophosmin OS=Homo sapiens OX=9606 GN=NPM1 PE=1 SV=2 |
| Q05639\|EF1A2_HUMAN | 320.9 | 50470 | Elongation factor 1-alpha 2 OS=Homo sapiens OX=9606 GN=EEF1A2 PE=1 SV=1 |
| P21281\|VATB2_HUMAN | 320.6 | 56501 | V-type proton ATPase subunit B brain isoform OS=Homo sapiens OX=9606 GN=ATP6V1B2 PE=1 SV=3 |
| P38159\|RBMX_HUMAN | 320.34 | 42332 | RNA-binding motif protein X chromosome OS=Homo sapiens OX=9606 GN=RBMX PE=1 SV=3 |
| Q9P0K7\|RAI14_HUMAN | 318.52 | 110041 | Ankycorbin OS=Homo sapiens OX=9606 GN=RAI14 PE=1 SV=2 |
| P16402\|H13_HUMAN | 318.22 | 22350 | Histone H1.3 OS=Homo sapiens OX=9606 GN=HIST1H1D PE=1 SV=2 |
| P46821\|MAP1B_HUMAN | 316.77 | 270632 | Microtubule-associated protein 1B OS=Homo sapiens OX=9606 GN=MAP1B PE=1 SV=2 |
| P10412\|H14_HUMAN | 316.62 | 21865 | Histone H1.4 OS=Homo sapiens OX=9606 GN=HIST1H1E PE=1 SV=2 |
| P16403\|H12_HUMAN | 316.42 | 21365 | Histone H1.2 OS=Homo sapiens OX=9606 GN=HIST1H1C PE=1 SV=2 |
| P27816\|MAP4_HUMAN | 316.35 | 121005 | Microtubule-associated protein 4 OS=Homo sapiens OX=9606 GN=MAP4 PE=1 SV=3 |
| Q9Y2D5\|AKAP2_HUMAN | 316.34 | 94661 | A-kinase anchor protein 2 OS=Homo sapiens OX=9606 GN=AKAP2 PE=1 SV=3 |
| Q16891\|MIC60_HUMAN | 314.27 | 83678 | MICOS complex subunit MIC60 OS=Homo sapiens OX=9606 GN=IMMT PE=1 SV=1 |
| P09651\|ROA1_HUMAN | 311.93 | 38747 | Heterogeneous nuclear ribonucleoprotein A1 OS=Homo sapiens OX=9606 GN=HNRNPA1 PE=1 SV=5 |
| P16401\|H15_HUMAN | 311.83 | 22580 | Histone H1.5 OS=Homo sapiens OX=9606 GN=HIST1H1B PE=1 SV=3 |
| Q14315\|FLNC_HUMAN | 310.7 | 291020 | Filamin-C OS=Homo sapiens OX=9606 GN=FLNC PE=1 SV=3 |
| P23284\|PPIB_HUMAN | 308.04 | 23743 | Peptidyl-prolyl cis-trans isomerase B OS=Homo sapiens OX=9606 GN=PPIB PE=1 SV=2 |
| O75369\|FLNB_HUMAN | 307.82 | 278162 | Filamin-B OS=Homo sapiens OX=9606 GN=FLNB PE=1 SV=2 |
| Q99623\|PHB2_HUMAN | 307.59 | 33296 | Prohibitin-2 OS=Homo sapiens OX=9606 GN=PHB2 PE=1 SV=2 |
| Q12906\|ILF3_HUMAN | 306.9 | 95339 | Interleukin enhancer-binding factor 3 OS=Homo sapiens OX=9606 GN=ILF3 PE=1 SV=3 |
| P29692\|EF1D_HUMAN | 302.48 | 31122 | Elongation factor 1-delta OS=Homo sapiens OX=9606 GN=EEF1D PE=1 SV=5 |
| Q9Y490\|TLN1_HUMAN | 302.14 | 269765 | Talin-1 OS=Homo sapiens OX=9606 GN=TLN1 PE=1 SV=3 |
| P60660\|MYL6_HUMAN | 300.43 | 16930 | Myosin light polypeptide 6 OS=Homo sapiens OX=9606 GN=MYL6 PE=1 SV=2 |
| Q9Y3I0\|RTCB_HUMAN | 297.51 | 55210 | tRNA-splicing ligase RtcB homolog OS=Homo sapiens OX=9606 GN=RTCB PE=1 SV=1 |
| P38606\|VATA_HUMAN | 296.93 | 68304 | V-type proton ATPase catalytic subunit A OS=Homo sapiens OX=9606 GN=ATP6V1A PE=1 SV=2 |
| Q9H2D6\|TARA_HUMAN | 296.39 | 261373 | TRIO and F-actin-binding protein OS=Homo sapiens OX=9606 GN=TRIOBP PE=1 SV=3 |
| Q13347\|EIF3I_HUMAN | 296.03 | 36502 | Eukaryotic translation initiation factor 3 subunit I OS=Homo sapiens OX=9606 GN=EIF3I PE=1 SV=1 |
| Q9UMS6\|SYNP2_HUMAN | 292.98 | 117514 | Synaptopodin-2 OS=Homo sapiens OX=9606 GN=SYNPO2 PE=1 SV=2 |
| Q14247\|SRC8_HUMAN | 290.58 | 61586 | Src substrate cortactin OS=Homo sapiens OX=9606 GN=CTTN PE=1 SV=2 |
| Q96PK6\|RBM14_HUMAN | 286.96 | 69492 | RNA-binding protein 14 OS=Homo sapiens OX=9606 GN=RBM14 PE=1 SV=2 |
| P0DP24\|CALM2_HUMAN | 286.05 | 16838 | Calmodulin-2 OS=Homo sapiens OX=9606 GN=CALM2 PE=1 SV=1 |
| P0DP23\|CALM1_HUMAN | 286.05 | 16838 | Calmodulin-1 OS=Homo sapiens OX=9606 GN=CALM1 PE=1 SV=1 |
| P0DP25\|CALM3_HUMAN | 286.05 | 16838 | Calmodulin-3 OS=Homo sapiens OX=9606 GN=CALM3 PE=1 SV=1 |
| Q04695\|K1C17_HUMAN | 285.96 | 48106 | Keratin type I cytoskeletal 17 OS=Homo sapiens OX=9606 GN=KRT17 PE=1 SV=2 |
| P07910\|HNRPC_HUMAN | 285.77 | 33670 | Heterogeneous nuclear ribonucleoproteins C1/C2 OS=Homo sapiens OX=9606 GN=HNRNPC PE=1 SV=4 |
| Q15155\|NOMO1_HUMAN | 284.8 | 134324 | Nodal modulator 1 OS=Homo sapiens OX=9606 GN=NOMO1 PE=1 SV=5 |
| Q5JPE7\|NOMO2_HUMAN | 284.04 | 139439 | Nodal modulator 2 OS=Homo sapiens OX=9606 GN=NOMO2 PE=1 SV=1 |
| P40939\|ECHA_HUMAN | 283.47 | 83000 | Trifunctional enzyme subunit alpha mitochondrial OS=Homo sapiens OX=9606 GN=HADHA PE=1 SV=2 |
| Q9ULV4\|COR1C_HUMAN | 282.3 | 53249 | Coronin-1C OS=Homo sapiens OX=9606 GN=CORO1C PE=1 SV=1 |
| P38646\|GRP75_HUMAN | 277.93 | 73681 | Stress-70 protein mitochondrial OS=Homo sapiens OX=9606 GN=HSPA9 PE=1 SV=2 |
| P23396\|RS3_HUMAN | 277.87 | 26688 | 40S ribosomal protein S3 OS=Homo sapiens OX=9606 GN=RPS3 PE=1 SV=2 |
| P06396\|GELS_HUMAN | 275.82 | 85697 | Gelsolin OS=Homo sapiens OX=9606 GN=GSN PE=1 SV=1 |
| P61978\|HNRPK_HUMAN | 272.26 | 50976 | Heterogeneous nuclear ribonucleoprotein K OS=Homo sapiens OX=9606 GN=HNRNPK PE=1 SV=1 |
| P54652\|HSP72_HUMAN | 271.5 | 70021 | Heat shock-related 70 kDa protein 2 OS=Homo sapiens OX=9606 GN=HSPA2 PE=1 SV=1 |
| O95425\|SVIL_HUMAN | 270.86 | 247744 | Supervillin OS=Homo sapiens OX=9606 GN=SVIL PE=1 SV=2 |
| Q9Y262\|EIF3L_HUMAN | 270.74 | 66727 | Eukaryotic translation initiation factor 3 subunit L OS=Homo sapiens OX=9606 GN=EIF3L PE=1 SV=1 |
| Q969G5\|CAVN3_HUMAN | 270.21 | 27701 | Caveolae-associated protein 3 OS=Homo sapiens OX=9606 GN=CAVIN3 PE=1 SV=3 |
| Q96E39\|RMXL1_HUMAN | 269.35 | 42142 | RNA binding motif protein X-linked-like-1 OS=Homo sapiens OX=9606 GN=RBMXL1 PE=1 SV=1 |
| P15924\|DESP_HUMAN | 269.05 | 331774 | Desmoplakin OS=Homo sapiens OX=9606 GN=DSP PE=1 SV=3 |
| P35232\|PHB_HUMAN | 269.01 | 29804 | Prohibitin OS=Homo sapiens OX=9606 GN=PHB PE=1 SV=1 |
| Q99715\|COCA1_HUMAN | 268.17 | 333147 | Collagen alpha-1(XII) chain OS=Homo sapiens OX=9606 GN=COL12A1 PE=1 SV=2 |
| P11940\|PABP1_HUMAN | 267.44 | 70671 | Polyadenylate-binding protein 1 OS=Homo sapiens OX=9606 GN=PABPC1 PE=1 SV=2 |
| P41219\|PERI_HUMAN | 267.36 | 53651 | Peripherin OS=Homo sapiens OX=9606 GN=PRPH PE=1 SV=2 |
| Q86V81\|THOC4_HUMAN | 267.11 | 26888 | THO complex subunit 4 OS=Homo sapiens OX=9606 GN=ALYREF PE=1 SV=3 |
| Q86YZ3\|HORN_HUMAN | 265.9 | 282389 | Hornerin OS=Homo sapiens OX=9606 GN=HRNR PE=1 SV=2 |
| O15372\|EIF3H_HUMAN | 265.36 | 39930 | Eukaryotic translation initiation factor 3 subunit H OS=Homo sapiens OX=9606 GN=EIF3H PE=1 SV=1 |
| Q0ZGT2\|NEXN_HUMAN | 264.78 | 80658 | Nexilin OS=Homo sapiens OX=9606 GN=NEXN PE=1 SV=1 |
| P17661\|DESM_HUMAN | 264.18 | 53536 | Desmin OS=Homo sapiens OX=9606 GN=DES PE=1 SV=3 |
| Q03252\|LMNB2_HUMAN | 263.98 | 69948 | Lamin-B2 OS=Homo sapiens OX=9606 GN=LMNB2 PE=1 SV=4 |
| Q9NR12\|PDLI7_HUMAN | 263.91 | 49845 | PDZ and LIM domain protein 7 OS=Homo sapiens OX=9606 GN=PDLIM7 PE=1 SV=1 |
| P26038\|MOES_HUMAN | 262.78 | 67820 | Moesin OS=Homo sapiens OX=9606 GN=MSN PE=1 SV=3 |
| P20700\|LMNB1_HUMAN | 262.73 | 66408 | Lamin-B1 OS=Homo sapiens OX=9606 GN=LMNB1 PE=1 SV=2 |
| Q15233\|NONO_HUMAN | 262.49 | 54232 | Non-POU domain-containing octamer-binding protein OS=Homo sapiens OX=9606 GN=NONO PE=1 SV=4 |
| O00303\|EIF3F_HUMAN | 261.57 | 37564 | Eukaryotic translation initiation factor 3 subunit F OS=Homo sapiens OX=9606 GN=EIF3F PE=1 SV=1 |
| P55884\|EIF3B_HUMAN | 261.52 | 92482 | Eukaryotic translation initiation factor 3 subunit B OS=Homo sapiens OX=9606 GN=EIF3B PE=1 SV=3 |
| Q16643\|DREB_HUMAN | 261.43 | 71429 | Drebrin OS=Homo sapiens OX=9606 GN=DBN1 PE=1 SV=4 |
| P41091\|IF2G_HUMAN | 259.6 | 51110 | Eukaryotic translation initiation factor 2 subunit 3 OS=Homo sapiens OX=9606 GN=EIF2S3 PE=1 SV=3 |
| P52907\|CAZA1_HUMAN | 258.17 | 32923 | F-actin-capping protein subunit alpha-1 OS=Homo sapiens OX=9606 GN=CAPZA1 PE=1 SV=3 |
| P07305\|H10_HUMAN | 258 | 20863 | Histone H1.0 OS=Homo sapiens OX=9606 GN=H1F0 PE=1 SV=3 |
| Q13283\|G3BP1_HUMAN | 257.61 | 52164 | Ras GTPase-activating protein-binding protein 1 OS=Homo sapiens OX=9606 GN=G3BP1 PE=1 SV=1 |
| Q07065\|CKAP4_HUMAN | 257.6 | 66023 | Cytoskeleton-associated protein 4 OS=Homo sapiens OX=9606 GN=CKAP4 PE=1 SV=2 |
| P51991\|ROA3_HUMAN | 257.46 | 39595 | Heterogeneous nuclear ribonucleoprotein A3 OS=Homo sapiens OX=9606 GN=HNRNPA3 PE=1 SV=2 |
| Q02539\|H11_HUMAN | 256.56 | 21842 | Histone H1.1 OS=Homo sapiens OX=9606 GN=H1-1 PE=1 SV=3 |
| P43235\|CATK_HUMAN | 255.59 | 36966 | Cathepsin K OS=Homo sapiens OX=9606 GN=CTSK PE=1 SV=1 |
| P02751\|FINC_HUMAN | 253.63 | 272318 | Fibronectin OS=Homo sapiens OX=9606 GN=FN1 PE=1 SV=5 |
| P04792\|HSPB1_HUMAN | 252.41 | 22783 | Heat shock protein beta-1 OS=Homo sapiens OX=9606 GN=HSPB1 PE=1 SV=2 |
| P07237\|PDIA1_HUMAN | 251.09 | 57116 | Protein disulfide-isomerase OS=Homo sapiens OX=9606 GN=P4HB PE=1 SV=3 |
| P22087\|FBRL_HUMAN | 250.73 | 33784 | rRNA 2'-O-methyltransferase fibrillarin OS=Homo sapiens OX=9606 GN=FBL PE=1 SV=2 |
| O75083\|WDR1_HUMAN | 249.89 | 66194 | WD repeat-containing protein 1 OS=Homo sapiens OX=9606 GN=WDR1 PE=1 SV=4 |
| P25705\|ATPA_HUMAN | 249.76 | 59751 | ATP synthase subunit alpha mitochondrial OS=Homo sapiens OX=9606 GN=ATP5F1A PE=1 SV=1 |
| O95816\|BAG2_HUMAN | 249.03 | 23772 | BAG family molecular chaperone regulator 2 OS=Homo sapiens OX=9606 GN=BAG2 PE=1 SV=1 |
| Q6WCQ1\|MPRIP_HUMAN | 248.68 | 116533 | Myosin phosphatase Rho-interacting protein OS=Homo sapiens OX=9606 GN=MPRIP PE=1 SV=3 |
| P62263\|RS14_HUMAN | 248.38 | 16273 | 40S ribosomal protein S14 OS=Homo sapiens OX=9606 GN=RPS14 PE=1 SV=3 |
| P39019\|RS19_HUMAN | 246.53 | 16060 | 40S ribosomal protein S19 OS=Homo sapiens OX=9606 GN=RPS19 PE=1 SV=2 |
| P07996\|TSP1_HUMAN | 245.28 | 129383 | Thrombospondin-1 OS=Homo sapiens OX=9606 GN=THBS1 PE=1 SV=2 |
| P47756\|CAPZB_HUMAN | 245.14 | 31350 | F-actin-capping protein subunit beta OS=Homo sapiens OX=9606 GN=CAPZB PE=1 SV=4 |
| P05787\|K2C8_HUMAN | 241.76 | 53704 | Keratin type II cytoskeletal 8 OS=Homo sapiens OX=9606 GN=KRT8 PE=1 SV=7 |
| P04406\|G3P_HUMAN | 241.57 | 36053 | Glyceraldehyde-3-phosphate dehydrogenase OS=Homo sapiens OX=9606 GN=GAPDH PE=1 SV=3 |
| P51659\|DHB4_HUMAN | 240.71 | 79686 | Peroxisomal multifunctional enzyme type 2 OS=Homo sapiens OX=9606 GN=HSD17B4 PE=1 SV=3 |
| Q02413\|DSG1_HUMAN | 240.65 | 113748 | Desmoglein-1 OS=Homo sapiens OX=9606 GN=DSG1 PE=1 SV=2 |
| P50454\|SERPH_HUMAN | 239.95 | 46441 | Serpin H1 OS=Homo sapiens OX=9606 GN=SERPINH1 PE=1 SV=2 |
| Q13310\|PABP4_HUMAN | 239.78 | 70783 | Polyadenylate-binding protein 4 OS=Homo sapiens OX=9606 GN=PABPC4 PE=1 SV=1 |
| P19338\|NUCL_HUMAN | 238.95 | 76615 | Nucleolin OS=Homo sapiens OX=9606 GN=NCL PE=1 SV=3 |
| P14866\|HNRPL_HUMAN | 237.78 | 64133 | Heterogeneous nuclear ribonucleoprotein L OS=Homo sapiens OX=9606 GN=HNRNPL PE=1 SV=2 |
| P13646\|K1C13_HUMAN | 237.69 | 49588 | Keratin type I cytoskeletal 13 OS=Homo sapiens OX=9606 GN=KRT13 PE=1 SV=4 |
| Q13151\|ROA0_HUMAN | 237.52 | 30841 | Heterogeneous nuclear ribonucleoprotein A0 OS=Homo sapiens OX=9606 GN=HNRNPA0 PE=1 SV=1 |
| Q5SSJ5\|HP1B3_HUMAN | 237.5 | 61207 | Heterochromatin protein 1-binding protein 3 OS=Homo sapiens OX=9606 GN=HP1BP3 PE=1 SV=1 |
| P63104\|1433Z_HUMAN | 237.15 | 27745 | 14-3-3 protein zeta/delta OS=Homo sapiens OX=9606 GN=YWHAZ PE=1 SV=1 |
| Q12905\|ILF2_HUMAN | 236.88 | 43062 | Interleukin enhancer-binding factor 2 OS=Homo sapiens OX=9606 GN=ILF2 PE=1 SV=2 |
| P61247\|RS3A_HUMAN | 236.5 | 29945 | 40S ribosomal protein S3a OS=Homo sapiens OX=9606 GN=RPS3A PE=1 SV=2 |
| P67809\|YBOX1_HUMAN | 236.39 | 35924 | Y-box-binding protein 1 OS=Homo sapiens OX=9606 GN=YBX1 PE=1 SV=3 |
| P19105\|ML12A_HUMAN | 235.29 | 19794 | Myosin regulatory light chain 12A OS=Homo sapiens OX=9606 GN=MYL12A PE=1 SV=2 |
| O14950\|ML12B_HUMAN | 235.29 | 19779 | Myosin regulatory light chain 12B OS=Homo sapiens OX=9606 GN=MYL12B PE=1 SV=2 |
| P28331\|NDUS1_HUMAN | 234.67 | 79468 | NADH-ubiquinone oxidoreductase 75 kDa subunit mitochondrial OS=Homo sapiens OX=9606 GN=NDUFS1 PE=1 SV=3 |
| Q14204\|DYHC1_HUMAN | 233.19 | 532412 | Cytoplasmic dynein 1 heavy chain 1 OS=Homo sapiens OX=9606 GN=DYNC1H1 PE=1 SV=5 |
| O43809\|CPSF5_HUMAN | 232.94 | 26227 | Cleavage and polyadenylation specificity factor subunit 5 OS=Homo sapiens OX=9606 GN=NUDT21 PE=1 SV=1 |
| P55084\|ECHB_HUMAN | 232.44 | 51294 | Trifunctional enzyme subunit beta mitochondrial OS=Homo sapiens OX=9606 GN=HADHB PE=1 SV=3 |
| Q92599\|SEPT8_HUMAN | 232.41 | 55756 | Septin-8 OS=Homo sapiens OX=9606 GN=SEPTIN8 PE=1 SV=4 |
| Q9BY77\|PDIP3_HUMAN | 231.96 | 46089 | Polymerase delta-interacting protein 3 OS=Homo sapiens OX=9606 GN=POLDIP3 PE=1 SV=2 |
| P52272\|HNRPM_HUMAN | 231.75 | 77516 | Heterogeneous nuclear ribonucleoprotein M OS=Homo sapiens OX=9606 GN=HNRNPM PE=1 SV=3 |
| P23246\|SFPQ_HUMAN | 231.49 | 76150 | Splicing factor proline- and glutamine-rich OS=Homo sapiens OX=9606 GN=SFPQ PE=1 SV=2 |
| Q9UKM9\|RALY_HUMAN | 230.88 | 32463 | RNA-binding protein Raly OS=Homo sapiens OX=9606 GN=RALY PE=1 SV=1 |
| P09382\|LEG1_HUMAN | 230.82 | 14716 | Galectin-1 OS=Homo sapiens OX=9606 GN=LGALS1 PE=1 SV=2 |
| Q06830\|PRDX1_HUMAN | 230.79 | 22110 | Peroxiredoxin-1 OS=Homo sapiens OX=9606 GN=PRDX1 PE=1 SV=1 |
| Q00610\|CLH1_HUMAN | 230.02 | 191613 | Clathrin heavy chain 1 OS=Homo sapiens OX=9606 GN=CLTC PE=1 SV=5 |
| P14618\|KPYM_HUMAN | 229.47 | 57937 | Pyruvate kinase PKM OS=Homo sapiens OX=9606 GN=PKM PE=1 SV=4 |
| P35609\|ACTN2_HUMAN | 229.32 | 103854 | Alpha-actinin-2 OS=Homo sapiens OX=9606 GN=ACTN2 PE=1 SV=1 |
| Q9NZN4\|EHD2_HUMAN | 228.95 | 61162 | EH domain-containing protein 2 OS=Homo sapiens OX=9606 GN=EHD2 PE=1 SV=2 |
| P14868\|SYDC_HUMAN | 228.17 | 57136 | Aspartate--tRNA ligase cytoplasmic OS=Homo sapiens OX=9606 GN=DARS PE=1 SV=2 |
| P61160\|ARP2_HUMAN | 227.7 | 44761 | Actin-related protein 2 OS=Homo sapiens OX=9606 GN=ACTR2 PE=1 SV=1 |
| P07196\|NFL_HUMAN | 227.12 | 61517 | Neurofilament light polypeptide OS=Homo sapiens OX=9606 GN=NEFL PE=1 SV=3 |
| Q15459\|SF3A1_HUMAN | 226.97 | 88886 | Splicing factor 3A subunit 1 OS=Homo sapiens OX=9606 GN=SF3A1 PE=1 SV=1 |
| O15371\|EIF3D_HUMAN | 226.92 | 63973 | Eukaryotic translation initiation factor 3 subunit D OS=Homo sapiens OX=9606 GN=EIF3D PE=1 SV=1 |
| Q7KZF4\|SND1_HUMAN | 226.49 | 101997 | Staphylococcal nuclease domain-containing protein 1 OS=Homo sapiens OX=9606 GN=SND1 PE=1 SV=1 |
| P62081\|RS7_HUMAN | 226.25 | 22127 | 40S ribosomal protein S7 OS=Homo sapiens OX=9606 GN=RPS7 PE=1 SV=1 |
| Q13435\|SF3B2_HUMAN | 225.98 | 100228 | Splicing factor 3B subunit 2 OS=Homo sapiens OX=9606 GN=SF3B2 PE=1 SV=2 |
| P07437\|TBB5_HUMAN | 225.47 | 49671 | Tubulin beta chain OS=Homo sapiens OX=9606 GN=TUBB PE=1 SV=2 |
| P78371\|TCPB_HUMAN | 225 | 57488 | T-complex protein 1 subunit beta OS=Homo sapiens OX=9606 GN=CCT2 PE=1 SV=4 |
| P62987\|RL40_HUMAN | 223.17 | 14728 | Ubiquitin-60S ribosomal protein L40 OS=Homo sapiens OX=9606 GN=UBA52 PE=1 SV=2 |
| P47755\|CAZA2_HUMAN | 222.36 | 32949 | F-actin-capping protein subunit alpha-2 OS=Homo sapiens OX=9606 GN=CAPZA2 PE=1 SV=3 |
| P14923\|PLAK_HUMAN | 222.24 | 81745 | Junction plakoglobin OS=Homo sapiens OX=9606 GN=JUP PE=1 SV=3 |
| P23528\|COF1_HUMAN | 222.1 | 18502 | Cofilin-1 OS=Homo sapiens OX=9606 GN=CFL1 PE=1 SV=3 |
| P26373\|RL13_HUMAN | 221.42 | 24261 | 60S ribosomal protein L13 OS=Homo sapiens OX=9606 GN=RPL13 PE=1 SV=4 |
| P35749\|MYH11_HUMAN | 221.39 | 227337 | Myosin-11 OS=Homo sapiens OX=9606 GN=MYH11 PE=1 SV=3 |
| Q9P0V9\|SEP10_HUMAN | 220.91 | 52593 | Septin-10 OS=Homo sapiens OX=9606 GN=SEPTIN10 PE=1 SV=2 |
| P08123\|CO1A2_HUMAN | 220.61 | 129314 | Collagen alpha-2(I) chain OS=Homo sapiens OX=9606 GN=COL1A2 PE=1 SV=7 |
| P62829\|RL23_HUMAN | 220.08 | 14865 | 60S ribosomal protein L23 OS=Homo sapiens OX=9606 GN=RPL23 PE=1 SV=1 |
| P60866\|RS20_HUMAN | 219.27 | 13373 | 40S ribosomal protein S20 OS=Homo sapiens OX=9606 GN=RPS20 PE=1 SV=1 |
| P25786\|PSA1_HUMAN | 218.77 | 29556 | Proteasome subunit alpha type-1 OS=Homo sapiens OX=9606 GN=PSMA1 PE=1 SV=1 |
| Q9NX63\|MIC19_HUMAN | 218.53 | 26152 | MICOS complex subunit MIC19 OS=Homo sapiens OX=9606 GN=CHCHD3 PE=1 SV=1 |
| P68371\|TBB4B_HUMAN | 218.09 | 49831 | Tubulin beta-4B chain OS=Homo sapiens OX=9606 GN=TUBB4B PE=1 SV=1 |
| Q9NYL9\|TMOD3_HUMAN | 217.07 | 39595 | Tropomodulin-3 OS=Homo sapiens OX=9606 GN=TMOD3 PE=1 SV=1 |
| O75955\|FLOT1_HUMAN | 216.71 | 47355 | Flotillin-1 OS=Homo sapiens OX=9606 GN=FLOT1 PE=1 SV=3 |
| P60228\|EIF3E_HUMAN | 215.98 | 52221 | Eukaryotic translation initiation factor 3 subunit E OS=Homo sapiens OX=9606 GN=EIF3E PE=1 SV=1 |
| P27695\|APEX1_HUMAN | 215.33 | 35555 | DNA-(apurinic or apyrimidinic site) lyase OS=Homo sapiens OX=9606 GN=APEX1 PE=1 SV=2 |
| O94832\|MYO1D_HUMAN | 214.88 | 116202 | Unconventional myosin-Id OS=Homo sapiens OX=9606 GN=MYO1D PE=1 SV=2 |
| P46782\|RS5_HUMAN | 214.28 | 22876 | 40S ribosomal protein S5 OS=Homo sapiens OX=9606 GN=RPS5 PE=1 SV=4 |
| P12111\|CO6A3_HUMAN | 213.9 | 343668 | Collagen alpha-3(VI) chain OS=Homo sapiens OX=9606 GN=COL6A3 PE=1 SV=5 |
| Q15717\|ELAV1_HUMAN | 213.33 | 36092 | ELAV-like protein 1 OS=Homo sapiens OX=9606 GN=ELAVL1 PE=1 SV=2 |
| P62140\|PP1B_HUMAN | 212.75 | 37187 | Serine/threonine-protein phosphatase PP1-beta catalytic subunit OS=Homo sapiens OX=9606 GN=PPP1CB PE=1 SV=3 |
| P84090\|ERH_HUMAN | 212.05 | 12259 | Enhancer of rudimentary homolog OS=Homo sapiens OX=9606 GN=ERH PE=1 SV=1 |
| P26641\|EF1G_HUMAN | 211.07 | 50119 | Elongation factor 1-gamma OS=Homo sapiens OX=9606 GN=EEF1G PE=1 SV=3 |
| P32119\|PRDX2_HUMAN | 211.02 | 21892 | Peroxiredoxin-2 OS=Homo sapiens OX=9606 GN=PRDX2 PE=1 SV=5 |
| O43660\|PLRG1_HUMAN | 210.39 | 57194 | Pleiotropic regulator 1 OS=Homo sapiens OX=9606 GN=PLRG1 PE=1 SV=1 |
| O43684\|BUB3_HUMAN | 210.26 | 37155 | Mitotic checkpoint protein BUB3 OS=Homo sapiens OX=9606 GN=BUB3 PE=1 SV=1 |
| P0DMV8\|HS71A_HUMAN | 210.22 | 70052 | Heat shock 70 kDa protein 1A OS=Homo sapiens OX=9606 GN=HSPA1A PE=1 SV=1 |
| P0DMV9\|HS71B_HUMAN | 210.22 | 70052 | Heat shock 70 kDa protein 1B OS=Homo sapiens OX=9606 GN=HSPA1B PE=1 SV=1 |
| Q9UN86\|G3BP2_HUMAN | 209.88 | 54121 | Ras GTPase-activating protein-binding protein 2 OS=Homo sapiens OX=9606 GN=G3BP2 PE=1 SV=2 |
| P62136\|PP1A_HUMAN | 209.69 | 37512 | Serine/threonine-protein phosphatase PP1-alpha catalytic subunit OS=Homo sapiens OX=9606 GN=PPP1CA PE=1 SV=1 |
| P62913\|RL11_HUMAN | 209.02 | 20252 | 60S ribosomal protein L11 OS=Homo sapiens OX=9606 GN=RPL11 PE=1 SV=2 |
| P50990\|TCPQ_HUMAN | 208.8 | 59621 | T-complex protein 1 subunit theta OS=Homo sapiens OX=9606 GN=CCT8 PE=1 SV=4 |
| P25787\|PSA2_HUMAN | 208.48 | 25899 | Proteasome subunit alpha type-2 OS=Homo sapiens OX=9606 GN=PSMA2 PE=1 SV=2 |
| P37108\|SRP14_HUMAN | 207.97 | 14570 | Signal recognition particle 14 kDa protein OS=Homo sapiens OX=9606 GN=SRP14 PE=1 SV=2 |
| P46783\|RS10_HUMAN | 207.49 | 18898 | 40S ribosomal protein S10 OS=Homo sapiens OX=9606 GN=RPS10 PE=1 SV=1 |
| Q9BR76\|COR1B_HUMAN | 206.53 | 54235 | Coronin-1B OS=Homo sapiens OX=9606 GN=CORO1B PE=1 SV=1 |
| P47914\|RL29_HUMAN | 206.41 | 17752 | 60S ribosomal protein L29 OS=Homo sapiens OX=9606 GN=RPL29 PE=1 SV=2 |
| Q15393\|SF3B3_HUMAN | 206.32 | 135577 | Splicing factor 3B subunit 3 OS=Homo sapiens OX=9606 GN=SF3B3 PE=1 SV=4 |
| P17844\|DDX5_HUMAN | 206.04 | 69148 | Probable ATP-dependent RNA helicase DDX5 OS=Homo sapiens OX=9606 GN=DDX5 PE=1 SV=1 |
| Q7L2H7\|EIF3M_HUMAN | 205.79 | 42503 | Eukaryotic translation initiation factor 3 subunit M OS=Homo sapiens OX=9606 GN=EIF3M PE=1 SV=1 |
| Q9BY44\|EIF2A_HUMAN | 205.17 | 64990 | Eukaryotic translation initiation factor 2A OS=Homo sapiens OX=9606 GN=EIF2A PE=1 SV=3 |
| P60900\|PSA6_HUMAN | 204.59 | 27399 | Proteasome subunit alpha type-6 OS=Homo sapiens OX=9606 GN=PSMA6 PE=1 SV=1 |
| P46779\|RL28_HUMAN | 203.53 | 15747 | 60S ribosomal protein L28 OS=Homo sapiens OX=9606 GN=RPL28 PE=1 SV=3 |
| P36873\|PP1G_HUMAN | 203.31 | 36984 | Serine/threonine-protein phosphatase PP1-gamma catalytic subunit OS=Homo sapiens OX=9606 GN=PPP1CC PE=1 SV=1 |
| P35268\|RL22_HUMAN | 202.9 | 14787 | 60S ribosomal protein L22 OS=Homo sapiens OX=9606 GN=RPL22 PE=1 SV=2 |
| P61158\|ARP3_HUMAN | 202.71 | 47371 | Actin-related protein 3 OS=Homo sapiens OX=9606 GN=ACTR3 PE=1 SV=3 |
| Q16531\|DDB1_HUMAN | 201.9 | 126968 | DNA damage-binding protein 1 OS=Homo sapiens OX=9606 GN=DDB1 PE=1 SV=1 |
| P05387\|RLA2_HUMAN | 201.85 | 11665 | 60S acidic ribosomal protein P2 OS=Homo sapiens OX=9606 GN=RPLP2 PE=1 SV=1 |
| Q96AE4\|FUBP1_HUMAN | 201.85 | 67560 | Far upstream element-binding protein 1 OS=Homo sapiens OX=9606 GN=FUBP1 PE=1 SV=3 |
| Q9BZF9\|UACA_HUMAN | 201.82 | 162504 | Uveal autoantigen with coiled-coil domains and ankyrin repeats OS=Homo sapiens OX=9606 GN=UACA PE=1 SV=2 |
| Q13409\|DC1I2_HUMAN | 201.53 | 71457 | Cytoplasmic dynein 1 intermediate chain 2 OS=Homo sapiens OX=9606 GN=DYNC1I2 PE=1 SV=3 |
| P62873\|GBB1_HUMAN | 201.21 | 37377 | Guanine nucleotide-binding protein G(I)/G(S)/G(T) subunit beta-1 OS=Homo sapiens OX=9606 GN=GNB1 PE=1 SV=3 |
| Q63ZY3\|KANK2_HUMAN | 201.12 | 91174 | KN motif and ankyrin repeat domain-containing protein 2 OS=Homo sapiens OX=9606 GN=KANK2 PE=1 SV=1 |
| Q9BXF6\|RFIP5_HUMAN | 200.24 | 70415 | Rab11 family-interacting protein 5 OS=Homo sapiens OX=9606 GN=RAB11FIP5 PE=1 SV=1 |
| Q6NZI2\|CAVN1_HUMAN | 199.7 | 43476 | Caveolae-associated protein 1 OS=Homo sapiens OX=9606 GN=CAVIN1 PE=1 SV=1 |
| O96019\|ACL6A_HUMAN | 199.47 | 47461 | Actin-like protein 6A OS=Homo sapiens OX=9606 GN=ACTL6A PE=1 SV=1 |
| P24534\|EF1B_HUMAN | 198.67 | 24764 | Elongation factor 1-beta OS=Homo sapiens OX=9606 GN=EEF1B2 PE=1 SV=3 |
| P62318\|SMD3_HUMAN | 197.72 | 13916 | Small nuclear ribonucleoprotein Sm D3 OS=Homo sapiens OX=9606 GN=SNRPD3 PE=1 SV=1 |
| Q9BWS9\|CHID1_HUMAN | 196.55 | 44941 | Chitinase domain-containing protein 1 OS=Homo sapiens OX=9606 GN=CHID1 PE=1 SV=1 |
| P62249\|RS16_HUMAN | 196.36 | 16445 | 40S ribosomal protein S16 OS=Homo sapiens OX=9606 GN=RPS16 PE=1 SV=2 |
| P15311\|EZRI_HUMAN | 194.72 | 69413 | Ezrin OS=Homo sapiens OX=9606 GN=EZR PE=1 SV=4 |
| Q07666\|KHDR1_HUMAN | 193.99 | 48227 | KH domain-containing RNA-binding signal transduction-associated protein 1 OS=Homo sapiens OX=9606 GN=KHDRBS1 PE=1 SV=1 |
| O15061\|SYNEM_HUMAN | 193.56 | 172767 | Synemin OS=Homo sapiens OX=9606 GN=SYNM PE=1 SV=2 |
| P55769\|NH2L1_HUMAN | 193.24 | 14174 | NHP2-like protein 1 OS=Homo sapiens OX=9606 GN=SNU13 PE=1 SV=3 |
| Q9Y224\|RTRAF_HUMAN | 192.93 | 28068 | RNA transcription translation and transport factor protein OS=Homo sapiens OX=9606 GN=RTRAF PE=1 SV=1 |
| Q92743\|HTRA1_HUMAN | 192.73 | 51287 | Serine protease HTRA1 OS=Homo sapiens OX=9606 GN=HTRA1 PE=1 SV=1 |
| P49748\|ACADV_HUMAN | 192.61 | 70390 | Very long-chain specific acyl-CoA dehydrogenase mitochondrial OS=Homo sapiens OX=9606 GN=ACADVL PE=1 SV=1 |
| Q9Y281\|COF2_HUMAN | 192.53 | 18737 | Cofilin-2 OS=Homo sapiens OX=9606 GN=CFL2 PE=1 SV=1 |
| Q96DI7\|SNR40_HUMAN | 192.33 | 39311 | U5 small nuclear ribonucleoprotein 40 kDa protein OS=Homo sapiens OX=9606 GN=SNRNP40 PE=1 SV=1 |
| Q15404\|RSU1_HUMAN | 192.31 | 31540 | Ras suppressor protein 1 OS=Homo sapiens OX=9606 GN=RSU1 PE=1 SV=3 |
| P62280\|RS11_HUMAN | 192.27 | 18431 | 40S ribosomal protein S11 OS=Homo sapiens OX=9606 GN=RPS11 PE=1 SV=3 |
| O43795\|MYO1B_HUMAN | 192.25 | 131985 | Unconventional myosin-Ib OS=Homo sapiens OX=9606 GN=MYO1B PE=1 SV=3 |
| P05198\|IF2A_HUMAN | 191.89 | 36112 | Eukaryotic translation initiation factor 2 subunit 1 OS=Homo sapiens OX=9606 GN=EIF2S1 PE=1 SV=3 |
| P69905\|HBA_HUMAN | 191.83 | 15258 | Hemoglobin subunit alpha OS=Homo sapiens OX=9606 GN=HBA1 PE=1 SV=2 |
| P14625\|ENPL_HUMAN | 191.24 | 92469 | Endoplasmin OS=Homo sapiens OX=9606 GN=HSP90B1 PE=1 SV=1 |
| P19012\|K1C15_HUMAN | 190.09 | 49212 | Keratin type I cytoskeletal 15 OS=Homo sapiens OX=9606 GN=KRT15 PE=1 SV=3 |
| P19013\|K2C4_HUMAN | 190.02 | 57285 | Keratin type II cytoskeletal 4 OS=Homo sapiens OX=9606 GN=KRT4 PE=1 SV=4 |
| Q14974\|IMB1_HUMAN | 189.9 | 97170 | Importin subunit beta-1 OS=Homo sapiens OX=9606 GN=KPNB1 PE=1 SV=2 |
| O15143\|ARC1B_HUMAN | 189.17 | 40950 | Actin-related protein 2/3 complex subunit 1B OS=Homo sapiens OX=9606 GN=ARPC1B PE=1 SV=3 |
| P62879\|GBB2_HUMAN | 189.08 | 37331 | Guanine nucleotide-binding protein G(I)/G(S)/G(T) subunit beta-2 OS=Homo sapiens OX=9606 GN=GNB2 PE=1 SV=3 |
| Q13561\|DCTN2_HUMAN | 188.99 | 44231 | Dynactin subunit 2 OS=Homo sapiens OX=9606 GN=DCTN2 PE=1 SV=4 |
| Q92841\|DDX17_HUMAN | 188.98 | 80273 | Probable ATP-dependent RNA helicase DDX17 OS=Homo sapiens OX=9606 GN=DDX17 PE=1 SV=2 |
| P02768\|ALBU_HUMAN | 188.73 | 69367 | Serum albumin OS=Homo sapiens OX=9606 GN=ALB PE=1 SV=2 |
| Q96IZ0\|PAWR_HUMAN | 188.47 | 36568 | PRKC apoptosis WT1 regulator protein OS=Homo sapiens OX=9606 GN=PAWR PE=1 SV=1 |
| Q13418\|ILK_HUMAN | 188.38 | 51419 | Integrin-linked protein kinase OS=Homo sapiens OX=9606 GN=ILK PE=1 SV=2 |
| Q9NZR1\|TMOD2_HUMAN | 187.73 | 39595 | Tropomodulin-2 OS=Homo sapiens OX=9606 GN=TMOD2 PE=1 SV=1 |
| Q9NZT1\|CALL5_HUMAN | 187.58 | 15893 | Calmodulin-like protein 5 OS=Homo sapiens OX=9606 GN=CALML5 PE=1 SV=2 |
| Q14258\|TRI25_HUMAN | 187.56 | 70974 | E3 ubiquitin/ISG15 ligase TRIM25 OS=Homo sapiens OX=9606 GN=TRIM25 PE=1 SV=2 |
| P35222\|CTNB1_HUMAN | 187.35 | 85497 | Catenin beta-1 OS=Homo sapiens OX=9606 GN=CTNNB1 PE=1 SV=1 |
| P10809\|CH60_HUMAN | 187.09 | 61055 | 60 kDa heat shock protein mitochondrial OS=Homo sapiens OX=9606 GN=HSPD1 PE=1 SV=2 |
| P35241\|RADI_HUMAN | 186.71 | 68564 | Radixin OS=Homo sapiens OX=9606 GN=RDX PE=1 SV=1 |
| P41227\|NAA10_HUMAN | 186.7 | 26458 | N-alpha-acetyltransferase 10 OS=Homo sapiens OX=9606 GN=NAA10 PE=1 SV=1 |
| Q92572\|AP3S1_HUMAN | 186.43 | 21732 | AP-3 complex subunit sigma-1 OS=Homo sapiens OX=9606 GN=AP3S1 PE=1 SV=1 |
| Q8NEU8\|DP13B_HUMAN | 186.3 | 74493 | DCC-interacting protein 13-beta OS=Homo sapiens OX=9606 GN=APPL2 PE=1 SV=3 |
| Q9C005\|DPY30_HUMAN | 186 | 11250 | Protein dpy-30 homolog OS=Homo sapiens OX=9606 GN=DPY30 PE=1 SV=1 |
| Q92499\|DDX1_HUMAN | 185.22 | 82432 | ATP-dependent RNA helicase DDX1 OS=Homo sapiens OX=9606 GN=DDX1 PE=1 SV=2 |
| Q9Y230\|RUVB2_HUMAN | 185.07 | 51157 | RuvB-like 2 OS=Homo sapiens OX=9606 GN=RUVBL2 PE=1 SV=3 |
| Q6P1L8\|RM14_HUMAN | 184.93 | 15948 | 39S ribosomal protein L14 mitochondrial OS=Homo sapiens OX=9606 GN=MRPL14 PE=1 SV=1 |
| P63173\|RL38_HUMAN | 184.19 | 8218 | 60S ribosomal protein L38 OS=Homo sapiens OX=9606 GN=RPL38 PE=1 SV=2 |
| P02452\|CO1A1_HUMAN | 183.98 | 138942 | Collagen alpha-1(I) chain OS=Homo sapiens OX=9606 GN=COL1A1 PE=1 SV=5 |
| P09661\|RU2A_HUMAN | 183.89 | 28416 | U2 small nuclear ribonucleoprotein A' OS=Homo sapiens OX=9606 GN=SNRPA1 PE=1 SV=2 |
| Q8N1F7\|NUP93_HUMAN | 183.25 | 93488 | Nuclear pore complex protein Nup93 OS=Homo sapiens OX=9606 GN=NUP93 PE=1 SV=2 |
| P78406\|RAE1L_HUMAN | 182.99 | 40968 | mRNA export factor OS=Homo sapiens OX=9606 GN=RAE1 PE=1 SV=1 |
| P36543\|VATE1_HUMAN | 182.73 | 26145 | V-type proton ATPase subunit E 1 OS=Homo sapiens OX=9606 GN=ATP6V1E1 PE=1 SV=1 |
| P62258\|1433E_HUMAN | 182.27 | 29174 | 14-3-3 protein epsilon OS=Homo sapiens OX=9606 GN=YWHAE PE=1 SV=1 |
| Q99470\|SDF2_HUMAN | 182.14 | 23026 | Stromal cell-derived factor 2 OS=Homo sapiens OX=9606 GN=SDF2 PE=1 SV=2 |
| P51911\|CNN1_HUMAN | 181.52 | 33170 | Calponin-1 OS=Homo sapiens OX=9606 GN=CNN1 PE=1 SV=2 |
| O43166\|SI1L1_HUMAN | 181.48 | 200028 | Signal-induced proliferation-associated 1-like protein 1 OS=Homo sapiens OX=9606 GN=SIPA1L1 PE=1 SV=4 |
| Q8N1G4\|LRC47_HUMAN | 181.37 | 63473 | Leucine-rich repeat-containing protein 47 OS=Homo sapiens OX=9606 GN=LRRC47 PE=1 SV=1 |
| O95782\|AP2A1_HUMAN | 180.98 | 107546 | AP-2 complex subunit alpha-1 OS=Homo sapiens OX=9606 GN=AP2A1 PE=1 SV=3 |
| P62750\|RL23A_HUMAN | 180.65 | 17695 | 60S ribosomal protein L23a OS=Homo sapiens OX=9606 GN=RPL23A PE=1 SV=1 |
| P61981\|1433G_HUMAN | 180.45 | 28303 | 14-3-3 protein gamma OS=Homo sapiens OX=9606 GN=YWHAG PE=1 SV=2 |
| Q8WXF1\|PSPC1_HUMAN | 180.36 | 58744 | Paraspeckle component 1 OS=Homo sapiens OX=9606 GN=PSPC1 PE=1 SV=1 |
| P62906\|RL10A_HUMAN | 179.58 | 24831 | 60S ribosomal protein L10a OS=Homo sapiens OX=9606 GN=RPL10A PE=1 SV=2 |
| P24844\|MYL9_HUMAN | 179.46 | 19827 | Myosin regulatory light polypeptide 9 OS=Homo sapiens OX=9606 GN=MYL9 PE=1 SV=4 |
| Q6PJT7\|ZC3HE_HUMAN | 179.34 | 82876 | Zinc finger CCCH domain-containing protein 14 OS=Homo sapiens OX=9606 GN=ZC3H14 PE=1 SV=1 |
| P35658\|NU214_HUMAN | 179.03 | 213618 | Nuclear pore complex protein Nup214 OS=Homo sapiens OX=9606 GN=NUP214 PE=1 SV=2 |
| P50479\|PDLI4_HUMAN | 178.93 | 35398 | PDZ and LIM domain protein 4 OS=Homo sapiens OX=9606 GN=PDLIM4 PE=1 SV=2 |
| O15144\|ARPC2_HUMAN | 178.85 | 34333 | Actin-related protein 2/3 complex subunit 2 OS=Homo sapiens OX=9606 GN=ARPC2 PE=1 SV=1 |
| P16989\|YBOX3_HUMAN | 178.77 | 40090 | Y-box-binding protein 3 OS=Homo sapiens OX=9606 GN=YBX3 PE=1 SV=4 |
| Q96I24\|FUBP3_HUMAN | 178.47 | 61640 | Far upstream element-binding protein 3 OS=Homo sapiens OX=9606 GN=FUBP3 PE=1 SV=2 |
| P62753\|RS6_HUMAN | 178.34 | 28681 | 40S ribosomal protein S6 OS=Homo sapiens OX=9606 GN=RPS6 PE=1 SV=1 |
| Q92945\|FUBP2_HUMAN | 178.24 | 73115 | Far upstream element-binding protein 2 OS=Homo sapiens OX=9606 GN=KHSRP PE=1 SV=4 |
| Q14157\|UBP2L_HUMAN | 177.57 | 114534 | Ubiquitin-associated protein 2-like OS=Homo sapiens OX=9606 GN=UBAP2L PE=1 SV=2 |
| P28066\|PSA5_HUMAN | 177.43 | 26411 | Proteasome subunit alpha type-5 OS=Homo sapiens OX=9606 GN=PSMA5 PE=1 SV=3 |
| P28300\|LYOX_HUMAN | 175.95 | 46944 | Protein-lysine 6-oxidase OS=Homo sapiens OX=9606 GN=LOX PE=1 SV=2 |
| P09038\|FGF2_HUMAN | 175.92 | 30770 | Fibroblast growth factor 2 OS=Homo sapiens OX=9606 GN=FGF2 PE=1 SV=3 |
| Q5JTV8\|TOIP1_HUMAN | 175.5 | 66248 | Torsin-1A-interacting protein 1 OS=Homo sapiens OX=9606 GN=TOR1AIP1 PE=1 SV=2 |
| P62316\|SMD2_HUMAN | 175.26 | 13527 | Small nuclear ribonucleoprotein Sm D2 OS=Homo sapiens OX=9606 GN=SNRPD2 PE=1 SV=1 |
| P18754\|RCC1_HUMAN | 175.13 | 44969 | Regulator of chromosome condensation OS=Homo sapiens OX=9606 GN=RCC1 PE=1 SV=1 |
| Q9BVC4\|LST8_HUMAN | 174.89 | 35876 | Target of rapamycin complex subunit LST8 OS=Homo sapiens OX=9606 GN=MLST8 PE=1 SV=1 |
| P25398\|RS12_HUMAN | 174.55 | 14515 | 40S ribosomal protein S12 OS=Homo sapiens OX=9606 GN=RPS12 PE=1 SV=3 |
| Q9Y2T2\|AP3M1_HUMAN | 173.58 | 46939 | AP-3 complex subunit mu-1 OS=Homo sapiens OX=9606 GN=AP3M1 PE=1 SV=1 |
| Q9UQB8\|BAIP2_HUMAN | 173.26 | 60868 | Brain-specific angiogenesis inhibitor 1-associated protein 2 OS=Homo sapiens OX=9606 GN=BAIAP2 PE=1 SV=1 |
| Q9NPE3\|NOP10_HUMAN | 173.25 | 7706 | H/ACA ribonucleoprotein complex subunit 3 OS=Homo sapiens OX=9606 GN=NOP10 PE=1 SV=1 |
| P08708\|RS17_HUMAN | 173.12 | 15550 | 40S ribosomal protein S17 OS=Homo sapiens OX=9606 GN=RPS17 PE=1 SV=2 |
| P04075\|ALDOA_HUMAN | 172.48 | 39420 | Fructose-bisphosphate aldolase A OS=Homo sapiens OX=9606 GN=ALDOA PE=1 SV=2 |
| P30101\|PDIA3_HUMAN | 172.37 | 56782 | Protein disulfide-isomerase A3 OS=Homo sapiens OX=9606 GN=PDIA3 PE=1 SV=4 |
| Q9BUF5\|TBB6_HUMAN | 172.37 | 49857 | Tubulin beta-6 chain OS=Homo sapiens OX=9606 GN=TUBB6 PE=1 SV=1 |
| Q13501\|SQSTM_HUMAN | 171.89 | 47687 | Sequestosome-1 OS=Homo sapiens OX=9606 GN=SQSTM1 PE=1 SV=1 |
| P83731\|RL24_HUMAN | 171.45 | 17779 | 60S ribosomal protein L24 OS=Homo sapiens OX=9606 GN=RPL24 PE=1 SV=1 |
| P62424\|RL7A_HUMAN | 171.45 | 29996 | 60S ribosomal protein L7a OS=Homo sapiens OX=9606 GN=RPL7A PE=1 SV=2 |
| P24593\|IBP5_HUMAN | 171.28 | 30570 | Insulin-like growth factor-binding protein 5 OS=Homo sapiens OX=9606 GN=IGFBP5 PE=1 SV=1 |
| P52926\|HMGA2_HUMAN | 170.97 | 11832 | High mobility group protein HMGI-C OS=Homo sapiens OX=9606 GN=HMGA2 PE=1 SV=1 |
| Q8WX93\|PALLD_HUMAN | 170.81 | 150564 | Palladin OS=Homo sapiens OX=9606 GN=PALLD PE=1 SV=3 |
| Q6ZMI0\|PPR21_HUMAN | 170.48 | 88314 | Protein phosphatase 1 regulatory subunit 21 OS=Homo sapiens OX=9606 GN=PPP1R21 PE=1 SV=1 |
| P0C0S5\|H2AZ_HUMAN | 170.44 | 13553 | Histone H2A.Z OS=Homo sapiens OX=9606 GN=H2AFZ PE=1 SV=2 |
| Q71UI9\|H2AV_HUMAN | 170.44 | 13509 | Histone H2A.V OS=Homo sapiens OX=9606 GN=H2AFV PE=1 SV=3 |
| P62826\|RAN_HUMAN | 170.06 | 24423 | GTP-binding nuclear protein Ran OS=Homo sapiens OX=9606 GN=RAN PE=1 SV=3 |
| O14979\|HNRDL_HUMAN | 169.23 | 46438 | Heterogeneous nuclear ribonucleoprotein D-like OS=Homo sapiens OX=9606 GN=HNRNPDL PE=1 SV=3 |
| P62266\|RS23_HUMAN | 169.14 | 15808 | 40S ribosomal protein S23 OS=Homo sapiens OX=9606 GN=RPS23 PE=1 SV=3 |
| P61254\|RL26_HUMAN | 168.38 | 17258 | 60S ribosomal protein L26 OS=Homo sapiens OX=9606 GN=RPL26 PE=1 SV=1 |
| Q9UNX3\|RL26L_HUMAN | 168.38 | 17256 | 60S ribosomal protein L26-like 1 OS=Homo sapiens OX=9606 GN=RPL26L1 PE=1 SV=1 |
| O75694\|NU155_HUMAN | 167.94 | 155199 | Nuclear pore complex protein Nup155 OS=Homo sapiens OX=9606 GN=NUP155 PE=1 SV=1 |
| Q99536\|VAT1_HUMAN | 167.62 | 41920 | Synaptic vesicle membrane protein VAT-1 homolog OS=Homo sapiens OX=9606 GN=VAT1 PE=1 SV=2 |
| P62857\|RS28_HUMAN | 166.75 | 7841 | 40S ribosomal protein S28 OS=Homo sapiens OX=9606 GN=RPS28 PE=1 SV=1 |
| Q9UM54\|MYO6_HUMAN | 166.66 | 149691 | Unconventional myosin-VI OS=Homo sapiens OX=9606 GN=MYO6 PE=1 SV=4 |
| P06733\|ENOA_HUMAN | 165.8 | 47169 | Alpha-enolase OS=Homo sapiens OX=9606 GN=ENO1 PE=1 SV=2 |
| Q16698\|DECR_HUMAN | 165.54 | 36068 | 2 4-dienoyl-CoA reductase mitochondrial OS=Homo sapiens OX=9606 GN=DECR1 PE=1 SV=1 |
| P62937\|PPIA_HUMAN | 165.19 | 18012 | Peptidyl-prolyl cis-trans isomerase A OS=Homo sapiens OX=9606 GN=PPIA PE=1 SV=2 |
| P68363\|TBA1B_HUMAN | 165.19 | 50152 | Tubulin alpha-1B chain OS=Homo sapiens OX=9606 GN=TUBA1B PE=1 SV=1 |
| Q71U36\|TBA1A_HUMAN | 165.19 | 50136 | Tubulin alpha-1A chain OS=Homo sapiens OX=9606 GN=TUBA1A PE=1 SV=1 |
| P32969\|RL9_HUMAN | 164.39 | 21863 | 60S ribosomal protein L9 OS=Homo sapiens OX=9606 GN=RPL9 PE=1 SV=1 |
| Q08554\|DSC1_HUMAN | 163.98 | 99987 | Desmocollin-1 OS=Homo sapiens OX=9606 GN=DSC1 PE=1 SV=2 |
| Q96C19\|EFHD2_HUMAN | 163.61 | 26697 | EF-hand domain-containing protein D2 OS=Homo sapiens OX=9606 GN=EFHD2 PE=1 SV=1 |
| P49790\|NU153_HUMAN | 163.09 | 153938 | Nuclear pore complex protein Nup153 OS=Homo sapiens OX=9606 GN=NUP153 PE=1 SV=2 |
| Q9BPW8\|NIPS1_HUMAN | 163 | 33310 | Protein NipSnap homolog 1 OS=Homo sapiens OX=9606 GN=NIPSNAP1 PE=1 SV=1 |
| P61513\|RL37A_HUMAN | 162.79 | 10275 | 60S ribosomal protein L37a OS=Homo sapiens OX=9606 GN=RPL37A PE=1 SV=2 |
| Q9NUP9\|LIN7C_HUMAN | 162.13 | 21834 | Protein lin-7 homolog C OS=Homo sapiens OX=9606 GN=LIN7C PE=1 SV=1 |
| Q96JB5\|CK5P3_HUMAN | 162.04 | 56921 | CDK5 regulatory subunit-associated protein 3 OS=Homo sapiens OX=9606 GN=CDK5RAP3 PE=1 SV=2 |
| Q5HYI8\|RABL3_HUMAN | 161.88 | 26423 | Rab-like protein 3 OS=Homo sapiens OX=9606 GN=RABL3 PE=1 SV=1 |
| Q96DA6\|TIM14_HUMAN | 161.56 | 12499 | Mitochondrial import inner membrane translocase subunit TIM14 OS=Homo sapiens OX=9606 GN=DNAJC19 PE=1 SV=3 |
| O75348\|VATG1_HUMAN | 161.5 | 13758 | V-type proton ATPase subunit G 1 OS=Homo sapiens OX=9606 GN=ATP6V1G1 PE=1 SV=3 |
| P47897\|SYQ_HUMAN | 161.24 | 87799 | Glutamine--tRNA ligase OS=Homo sapiens OX=9606 GN=QARS PE=1 SV=1 |
| O00487\|PSDE_HUMAN | 160.93 | 34577 | 26S proteasome non-ATPase regulatory subunit 14 OS=Homo sapiens OX=9606 GN=PSMD14 PE=1 SV=1 |
| Q86U42\|PABP2_HUMAN | 160.9 | 32749 | Polyadenylate-binding protein 2 OS=Homo sapiens OX=9606 GN=PABPN1 PE=1 SV=3 |
| Q9HCN8\|SDF2L_HUMAN | 160.81 | 23598 | Stromal cell-derived factor 2-like protein 1 OS=Homo sapiens OX=9606 GN=SDF2L1 PE=1 SV=2 |
| P50552\|VASP_HUMAN | 160.49 | 39830 | Vasodilator-stimulated phosphoprotein OS=Homo sapiens OX=9606 GN=VASP PE=1 SV=3 |
| P31946\|1433B_HUMAN | 159.66 | 28082 | 14-3-3 protein beta/alpha OS=Homo sapiens OX=9606 GN=YWHAB PE=1 SV=3 |
| C9JLW8\|MCRI1_HUMAN | 159.44 | 10920 | Mapk-regulated corepressor-interacting protein 1 OS=Homo sapiens OX=9606 GN=MCRIP1 PE=1 SV=1 |
| Q13573\|SNW1_HUMAN | 157.84 | 61495 | SNW domain-containing protein 1 OS=Homo sapiens OX=9606 GN=SNW1 PE=1 SV=1 |
| P62241\|RS8_HUMAN | 157.69 | 24205 | 40S ribosomal protein S8 OS=Homo sapiens OX=9606 GN=RPS8 PE=1 SV=2 |
| O75306\|NDUS2_HUMAN | 157.64 | 52546 | NADH dehydrogenase [ubiquinone] iron-sulfur protein 2 mitochondrial OS=Homo sapiens OX=9606 GN=NDUFS2 PE=1 SV=2 |
| Q8N1N4\|K2C78_HUMAN | 157.63 | 56866 | Keratin type II cytoskeletal 78 OS=Homo sapiens OX=9606 GN=KRT78 PE=1 SV=2 |
| P42766\|RL35_HUMAN | 157.12 | 14551 | 60S ribosomal protein L35 OS=Homo sapiens OX=9606 GN=RPL35 PE=1 SV=2 |
| P05362\|ICAM1_HUMAN | 157.09 | 57825 | Intercellular adhesion molecule 1 OS=Homo sapiens OX=9606 GN=ICAM1 PE=1 SV=2 |
| O14818\|PSA7_HUMAN | 157.06 | 27887 | Proteasome subunit alpha type-7 OS=Homo sapiens OX=9606 GN=PSMA7 PE=1 SV=1 |
| Q9Y2W1\|TR150_HUMAN | 156.65 | 108666 | Thyroid hormone receptor-associated protein 3 OS=Homo sapiens OX=9606 GN=THRAP3 PE=1 SV=2 |
| P33240\|CSTF2_HUMAN | 156.36 | 60959 | Cleavage stimulation factor subunit 2 OS=Homo sapiens OX=9606 GN=CSTF2 PE=1 SV=1 |
| Q9Y265\|RUVB1_HUMAN | 156.31 | 50228 | RuvB-like 1 OS=Homo sapiens OX=9606 GN=RUVBL1 PE=1 SV=1 |
| Q9Y3Y2\|CHTOP_HUMAN | 155.06 | 26397 | Chromatin target of PRMT1 protein OS=Homo sapiens OX=9606 GN=CHTOP PE=1 SV=2 |
| Q6KB66\|K2C80_HUMAN | 154.54 | 50525 | Keratin type II cytoskeletal 80 OS=Homo sapiens OX=9606 GN=KRT80 PE=1 SV=2 |
| Q13263\|TIF1B_HUMAN | 154.44 | 88550 | Transcription intermediary factor 1-beta OS=Homo sapiens OX=9606 GN=TRIM28 PE=1 SV=5 |
| Q14CN4\|K2C72_HUMAN | 154.33 | 55877 | Keratin type II cytoskeletal 72 OS=Homo sapiens OX=9606 GN=KRT72 PE=1 SV=2 |
| P07814\|SYEP_HUMAN | 154.29 | 170590 | Bifunctional glutamate/proline--tRNA ligase OS=Homo sapiens OX=9606 GN=EPRS PE=1 SV=5 |
| Q13557\|KCC2D_HUMAN | 154.28 | 56369 | Calcium/calmodulin-dependent protein kinase type II subunit delta OS=Homo sapiens OX=9606 GN=CAMK2D PE=1 SV=3 |
| P17987\|TCPA_HUMAN | 153.98 | 60344 | T-complex protein 1 subunit alpha OS=Homo sapiens OX=9606 GN=TCP1 PE=1 SV=1 |
| O00203\|AP3B1_HUMAN | 153.79 | 121320 | AP-3 complex subunit beta-1 OS=Homo sapiens OX=9606 GN=AP3B1 PE=1 SV=3 |
| Q15050\|RRS1_HUMAN | 153.62 | 41193 | Ribosome biogenesis regulatory protein homolog OS=Homo sapiens OX=9606 GN=RRS1 PE=1 SV=2 |
| Q16270\|IBP7_HUMAN | 153.34 | 29130 | Insulin-like growth factor-binding protein 7 OS=Homo sapiens OX=9606 GN=IGFBP7 PE=1 SV=1 |
| P16278\|BGAL_HUMAN | 152.51 | 76075 | Beta-galactosidase OS=Homo sapiens OX=9606 GN=GLB1 PE=1 SV=2 |
| Q16718\|NDUA5_HUMAN | 152.22 | 13459 | NADH dehydrogenase [ubiquinone] 1 alpha subcomplex subunit 5 OS=Homo sapiens OX=9606 GN=NDUFA5 PE=1 SV=3 |
| P36957\|ODO2_HUMAN | 152.19 | 48755 | Dihydrolipoyllysine-residue succinyltransferase component of 2-oxoglutarate dehydrogenase complex mitochondrial OS=Homo sapiens OX=9606 GN=DLST PE=1 SV=4 |
| P04083\|ANXA1_HUMAN | 151.89 | 38714 | Annexin A1 OS=Homo sapiens OX=9606 GN=ANXA1 PE=1 SV=2 |
| P04080\|CYTB_HUMAN | 151.8 | 11140 | Cystatin-B OS=Homo sapiens OX=9606 GN=CSTB PE=1 SV=2 |
| P09012\|SNRPA_HUMAN | 151.75 | 31280 | U1 small nuclear ribonucleoprotein A OS=Homo sapiens OX=9606 GN=SNRPA PE=1 SV=3 |
| Q15006\|EMC2_HUMAN | 151.58 | 34833 | ER membrane protein complex subunit 2 OS=Homo sapiens OX=9606 GN=EMC2 PE=1 SV=1 |
| Q8NCA5\|FA98A_HUMAN | 151.22 | 55272 | Protein FAM98A OS=Homo sapiens OX=9606 GN=FAM98A PE=1 SV=2 |
| P49458\|SRP09_HUMAN | 150.94 | 10112 | Signal recognition particle 9 kDa protein OS=Homo sapiens OX=9606 GN=SRP9 PE=1 SV=2 |
| Q13425\|SNTB2_HUMAN | 150.77 | 57950 | Beta-2-syntrophin OS=Homo sapiens OX=9606 GN=SNTB2 PE=1 SV=1 |
| P28074\|PSB5_HUMAN | 150.72 | 28480 | Proteasome subunit beta type-5 OS=Homo sapiens OX=9606 GN=PSMB5 PE=1 SV=3 |
| P02511\|CRYAB_HUMAN | 150.18 | 20159 | Alpha-crystallin B chain OS=Homo sapiens OX=9606 GN=CRYAB PE=1 SV=2 |
| O14558\|HSPB6_HUMAN | 149.42 | 17136 | Heat shock protein beta-6 OS=Homo sapiens OX=9606 GN=HSPB6 PE=1 SV=2 |
| P19388\|RPAB1_HUMAN | 149.23 | 24551 | DNA-directed RNA polymerases I II and III subunit RPABC1 OS=Homo sapiens OX=9606 GN=POLR2E PE=1 SV=4 |
| P48643\|TCPE_HUMAN | 149.07 | 59671 | T-complex protein 1 subunit epsilon OS=Homo sapiens OX=9606 GN=CCT5 PE=1 SV=1 |
| P62269\|RS18_HUMAN | 148.46 | 17719 | 40S ribosomal protein S18 OS=Homo sapiens OX=9606 GN=RPS18 PE=1 SV=3 |
| P59998\|ARPC4_HUMAN | 147.92 | 19667 | Actin-related protein 2/3 complex subunit 4 OS=Homo sapiens OX=9606 GN=ARPC4 PE=1 SV=3 |
| P52948\|NUP98_HUMAN | 147.42 | 197578 | Nuclear pore complex protein Nup98-Nup96 OS=Homo sapiens OX=9606 GN=NUP98 PE=1 SV=4 |
| Q00341\|VIGLN_HUMAN | 147.07 | 141455 | Vigilin OS=Homo sapiens OX=9606 GN=HDLBP PE=1 SV=2 |
| Q969G3\|SMCE1_HUMAN | 147.06 | 46649 | SWI/SNF-related matrix-associated actin-dependent regulator of chromatin subfamily E member 1 OS=Homo sapiens OX=9606 GN=SMARCE1 PE=1 SV=2 |
| Q9NP97\|DLRB1_HUMAN | 146.59 | 10922 | Dynein light chain roadblock-type 1 OS=Homo sapiens OX=9606 GN=DYNLRB1 PE=1 SV=3 |
| Q13200\|PSMD2_HUMAN | 145.38 | 100200 | 26S proteasome non-ATPase regulatory subunit 2 OS=Homo sapiens OX=9606 GN=PSMD2 PE=1 SV=3 |
| P60981\|DEST_HUMAN | 145.25 | 18506 | Destrin OS=Homo sapiens OX=9606 GN=DSTN PE=1 SV=3 |
| P62861\|RS30_HUMAN | 145.02 | 6648 | 40S ribosomal protein S30 OS=Homo sapiens OX=9606 GN=FAU PE=1 SV=1 |
| P82979\|SARNP_HUMAN | 144.63 | 23671 | SAP domain-containing ribonucleoprotein OS=Homo sapiens OX=9606 GN=SARNP PE=1 SV=3 |
| P46940\|IQGA1_HUMAN | 144.47 | 189251 | Ras GTPase-activating-like protein IQGAP1 OS=Homo sapiens OX=9606 GN=IQGAP1 PE=1 SV=1 |
| Q9H4G4\|GAPR1_HUMAN | 144.21 | 17218 | Golgi-associated plant pathogenesis-related protein 1 OS=Homo sapiens OX=9606 GN=GLIPR2 PE=1 SV=3 |
| P46776\|RL27A_HUMAN | 144.14 | 16561 | 60S ribosomal protein L27a OS=Homo sapiens OX=9606 GN=RPL27A PE=1 SV=2 |
| P51608\|MECP2_HUMAN | 143.98 | 52441 | Methyl-CpG-binding protein 2 OS=Homo sapiens OX=9606 GN=MECP2 PE=1 SV=1 |
| P04843\|RPN1_HUMAN | 143.91 | 68569 | Dolichyl-diphosphooligosaccharide--protein glycosyltransferase subunit 1 OS=Homo sapiens OX=9606 GN=RPN1 PE=1 SV=1 |
| Q92522\|H1X_HUMAN | 143.83 | 22487 | Histone H1x OS=Homo sapiens OX=9606 GN=H1FX PE=1 SV=1 |
| P46778\|RL21_HUMAN | 143.28 | 18565 | 60S ribosomal protein L21 OS=Homo sapiens OX=9606 GN=RPL21 PE=1 SV=2 |
| P62310\|LSM3_HUMAN | 143.27 | 11845 | U6 snRNA-associated Sm-like protein LSm3 OS=Homo sapiens OX=9606 GN=LSM3 PE=1 SV=2 |
| P30419\|NMT1_HUMAN | 142.74 | 56806 | Glycylpeptide N-tetradecanoyltransferase 1 OS=Homo sapiens OX=9606 GN=NMT1 PE=1 SV=2 |
| O43583\|DENR_HUMAN | 142.67 | 22092 | Density-regulated protein OS=Homo sapiens OX=9606 GN=DENR PE=1 SV=2 |
| Q86Y46\|K2C73_HUMAN | 142.35 | 58923 | Keratin type II cytoskeletal 73 OS=Homo sapiens OX=9606 GN=KRT73 PE=1 SV=1 |
| Q6UXN9\|WDR82_HUMAN | 142.3 | 35079 | WD repeat-containing protein 82 OS=Homo sapiens OX=9606 GN=WDR82 PE=1 SV=1 |
| Q14141\|SEPT6_HUMAN | 141.6 | 49717 | Septin-6 OS=Homo sapiens OX=9606 GN=SEPTIN6 PE=1 SV=4 |
| Q32MZ4\|LRRF1_HUMAN | 141.04 | 89253 | Leucine-rich repeat flightless-interacting protein 1 OS=Homo sapiens OX=9606 GN=LRRFIP1 PE=1 SV=2 |
| Q52LJ0\|FA98B_HUMAN | 141.04 | 45547 | Protein FAM98B OS=Homo sapiens OX=9606 GN=FAM98B PE=1 SV=2 |
| Q9NWH9\|SLTM_HUMAN | 140.64 | 117149 | SAFB-like transcription modulator OS=Homo sapiens OX=9606 GN=SLTM PE=1 SV=2 |
| Q96SB3\|NEB2_HUMAN | 140.61 | 89334 | Neurabin-2 OS=Homo sapiens OX=9606 GN=PPP1R9B PE=1 SV=3 |
| P60953\|CDC42_HUMAN | 140.57 | 21259 | Cell division control protein 42 homolog OS=Homo sapiens OX=9606 GN=CDC42 PE=1 SV=2 |
| P29401\|TKT_HUMAN | 140.01 | 67878 | Transketolase OS=Homo sapiens OX=9606 GN=TKT PE=1 SV=3 |
| O95182\|NDUA7_HUMAN | 139.93 | 12551 | NADH dehydrogenase [ubiquinone] 1 alpha subcomplex subunit 7 OS=Homo sapiens OX=9606 GN=NDUFA7 PE=1 SV=3 |
| O00151\|PDLI1_HUMAN | 139.87 | 36072 | PDZ and LIM domain protein 1 OS=Homo sapiens OX=9606 GN=PDLIM1 PE=1 SV=4 |
| Q7Z794\|K2C1B_HUMAN | 139.36 | 61901 | Keratin type II cytoskeletal 1b OS=Homo sapiens OX=9606 GN=KRT77 PE=2 SV=3 |
| P61289\|PSME3_HUMAN | 139.22 | 29506 | Proteasome activator complex subunit 3 OS=Homo sapiens OX=9606 GN=PSME3 PE=1 SV=1 |
| Q9UPN3\|MACF1_HUMAN | 137.95 | 838323 | Microtubule-actin cross-linking factor 1 isoforms 1/2/3/5 OS=Homo sapiens OX=9606 GN=MACF1 PE=1 SV=4 |
| P27487\|DPP4_HUMAN | 137.95 | 88279 | Dipeptidyl peptidase 4 OS=Homo sapiens OX=9606 GN=DPP4 PE=1 SV=2 |
| P50991\|TCPD_HUMAN | 137.61 | 57924 | T-complex protein 1 subunit delta OS=Homo sapiens OX=9606 GN=CCT4 PE=1 SV=4 |
| P62851\|RS25_HUMAN | 137.13 | 13742 | 40S ribosomal protein S25 OS=Homo sapiens OX=9606 GN=RPS25 PE=1 SV=1 |
| P62072\|TIM10_HUMAN | 136.8 | 10333 | Mitochondrial import inner membrane translocase subunit Tim10 OS=Homo sapiens OX=9606 GN=TIMM10 PE=1 SV=1 |
| P11047\|LAMC1_HUMAN | 136.73 | 177602 | Laminin subunit gamma-1 OS=Homo sapiens OX=9606 GN=LAMC1 PE=1 SV=3 |
| P62304\|RUXE_HUMAN | 136.62 | 10804 | Small nuclear ribonucleoprotein E OS=Homo sapiens OX=9606 GN=SNRPE PE=1 SV=1 |
| Q9Y241\|HIG1A_HUMAN | 136.15 | 10143 | HIG1 domain family member 1A mitochondrial OS=Homo sapiens OX=9606 GN=HIGD1A PE=1 SV=1 |
| P26640\|SYVC_HUMAN | 136.14 | 140476 | Valine--tRNA ligase OS=Homo sapiens OX=9606 GN=VARS PE=1 SV=4 |
| Q9UMS4\|PRP19_HUMAN | 135.19 | 55181 | Pre-mRNA-processing factor 19 OS=Homo sapiens OX=9606 GN=PRPF19 PE=1 SV=1 |
| O15511\|ARPC5_HUMAN | 135.17 | 16320 | Actin-related protein 2/3 complex subunit 5 OS=Homo sapiens OX=9606 GN=ARPC5 PE=1 SV=3 |
| Q99700\|ATX2_HUMAN | 135.03 | 140283 | Ataxin-2 OS=Homo sapiens OX=9606 GN=ATXN2 PE=1 SV=2 |
| P31944\|CASPE_HUMAN | 134.73 | 27680 | Caspase-14 OS=Homo sapiens OX=9606 GN=CASP14 PE=1 SV=2 |
| P55036\|PSMD4_HUMAN | 134.6 | 40737 | 26S proteasome non-ATPase regulatory subunit 4 OS=Homo sapiens OX=9606 GN=PSMD4 PE=1 SV=1 |
| P62917\|RL8_HUMAN | 134.32 | 28025 | 60S ribosomal protein L8 OS=Homo sapiens OX=9606 GN=RPL8 PE=1 SV=2 |
| Q14103\|HNRPD_HUMAN | 133.78 | 38434 | Heterogeneous nuclear ribonucleoprotein D0 OS=Homo sapiens OX=9606 GN=HNRNPD PE=1 SV=1 |
| Q96CW1\|AP2M1_HUMAN | 133.6 | 49655 | AP-2 complex subunit mu OS=Homo sapiens OX=9606 GN=AP2M1 PE=1 SV=2 |
| P09496\|CLCA_HUMAN | 133.1 | 27077 | Clathrin light chain A OS=Homo sapiens OX=9606 GN=CLTA PE=1 SV=1 |
| P37198\|NUP62_HUMAN | 132.86 | 53255 | Nuclear pore glycoprotein p62 OS=Homo sapiens OX=9606 GN=NUP62 PE=1 SV=3 |
| Q8NFH5\|NUP35_HUMAN | 132.75 | 34774 | Nucleoporin NUP35 OS=Homo sapiens OX=9606 GN=NUP35 PE=1 SV=1 |
| Q99714\|HCD2_HUMAN | 132.73 | 26923 | 3-hydroxyacyl-CoA dehydrogenase type-2 OS=Homo sapiens OX=9606 GN=HSD17B10 PE=1 SV=3 |
| P62847\|RS24_HUMAN | 132.45 | 15423 | 40S ribosomal protein S24 OS=Homo sapiens OX=9606 GN=RPS24 PE=1 SV=1 |
| P27348\|1433T_HUMAN | 131.88 | 27764 | 14-3-3 protein theta OS=Homo sapiens OX=9606 GN=YWHAQ PE=1 SV=1 |
| P62910\|RL32_HUMAN | 131.81 | 15860 | 60S ribosomal protein L32 OS=Homo sapiens OX=9606 GN=RPL32 PE=1 SV=2 |
| Q16186\|ADRM1_HUMAN | 131.76 | 42153 | Proteasomal ubiquitin receptor ADRM1 OS=Homo sapiens OX=9606 GN=ADRM1 PE=1 SV=2 |
| O60645\|EXOC3_HUMAN | 131.39 | 85567 | Exocyst complex component 3 OS=Homo sapiens OX=9606 GN=EXOC3 PE=1 SV=3 |
| Q9UBQ5\|EIF3K_HUMAN | 131.32 | 25060 | Eukaryotic translation initiation factor 3 subunit K OS=Homo sapiens OX=9606 GN=EIF3K PE=1 SV=1 |
| P05386\|RLA1_HUMAN | 131.19 | 11514 | 60S acidic ribosomal protein P1 OS=Homo sapiens OX=9606 GN=RPLP1 PE=1 SV=1 |
| P63010\|AP2B1_HUMAN | 131.03 | 104553 | AP-2 complex subunit beta OS=Homo sapiens OX=9606 GN=AP2B1 PE=1 SV=1 |
| P35611\|ADDA_HUMAN | 130.95 | 80955 | Alpha-adducin OS=Homo sapiens OX=9606 GN=ADD1 PE=1 SV=2 |
| P62277\|RS13_HUMAN | 130.73 | 17222 | 40S ribosomal protein S13 OS=Homo sapiens OX=9606 GN=RPS13 PE=1 SV=2 |
| Q96A65\|EXOC4_HUMAN | 130.69 | 110498 | Exocyst complex component 4 OS=Homo sapiens OX=9606 GN=EXOC4 PE=1 SV=1 |
| Q8NC51\|PAIRB_HUMAN | 130.09 | 44965 | Plasminogen activator inhibitor 1 RNA-binding protein OS=Homo sapiens OX=9606 GN=SERBP1 PE=1 SV=2 |
| Q9NYU2\|UGGG1_HUMAN | 129.86 | 177189 | UDP-glucose:glycoprotein glucosyltransferase 1 OS=Homo sapiens OX=9606 GN=UGGT1 PE=1 SV=3 |
| P30050\|RL12_HUMAN | 129.77 | 17819 | 60S ribosomal protein L12 OS=Homo sapiens OX=9606 GN=RPL12 PE=1 SV=1 |
| Q15366\|PCBP2_HUMAN | 128.47 | 38580 | Poly(rC)-binding protein 2 OS=Homo sapiens OX=9606 GN=PCBP2 PE=1 SV=1 |
| P49721\|PSB2_HUMAN | 128.15 | 22836 | Proteasome subunit beta type-2 OS=Homo sapiens OX=9606 GN=PSMB2 PE=1 SV=1 |
| P09936\|UCHL1_HUMAN | 127.37 | 24824 | Ubiquitin carboxyl-terminal hydrolase isozyme L1 OS=Homo sapiens OX=9606 GN=UCHL1 PE=1 SV=2 |
| Q13838\|DX39B_HUMAN | 127.22 | 48991 | Spliceosome RNA helicase DDX39B OS=Homo sapiens OX=9606 GN=DDX39B PE=1 SV=1 |
| O00148\|DX39A_HUMAN | 127.22 | 49130 | ATP-dependent RNA helicase DDX39A OS=Homo sapiens OX=9606 GN=DDX39A PE=1 SV=2 |
| P28070\|PSB4_HUMAN | 126.33 | 29204 | Proteasome subunit beta type-4 OS=Homo sapiens OX=9606 GN=PSMB4 PE=1 SV=4 |
| Q07954\|LRP1_HUMAN | 126.3 | 504610 | Prolow-density lipoprotein receptor-related protein 1 OS=Homo sapiens OX=9606 GN=LRP1 PE=1 SV=2 |
| O94776\|MTA2_HUMAN | 125.99 | 75023 | Metastasis-associated protein MTA2 OS=Homo sapiens OX=9606 GN=MTA2 PE=1 SV=1 |
| Q6RFH5\|WDR74_HUMAN | 125.86 | 42441 | WD repeat-containing protein 74 OS=Homo sapiens OX=9606 GN=WDR74 PE=1 SV=1 |
| O75947\|ATP5H_HUMAN | 125.81 | 18491 | ATP synthase subunit d mitochondrial OS=Homo sapiens OX=9606 GN=ATP5PD PE=1 SV=3 |
| P18206\|VINC_HUMAN | 125.79 | 123799 | Vinculin OS=Homo sapiens OX=9606 GN=VCL PE=1 SV=4 |
| P53582\|MAP11_HUMAN | 125.39 | 43215 | Methionine aminopeptidase 1 OS=Homo sapiens OX=9606 GN=METAP1 PE=1 SV=2 |
| Q8NB37\|GALD1_HUMAN | 125.29 | 23298 | Glutamine amidotransferase-like class 1 domain-containing protein 1 OS=Homo sapiens OX=9606 GN=GATD1 PE=1 SV=1 |
| Q15007\|FL2D_HUMAN | 125.28 | 44244 | Pre-mRNA-splicing regulator WTAP OS=Homo sapiens OX=9606 GN=WTAP PE=1 SV=2 |
| Q8IZP0\|ABI1_HUMAN | 125.1 | 55081 | Abl interactor 1 OS=Homo sapiens OX=9606 GN=ABI1 PE=1 SV=4 |
| Q04917\|1433F_HUMAN | 125.09 | 28219 | 14-3-3 protein eta OS=Homo sapiens OX=9606 GN=YWHAH PE=1 SV=4 |
| Q99567\|NUP88_HUMAN | 124.77 | 83542 | Nuclear pore complex protein Nup88 OS=Homo sapiens OX=9606 GN=NUP88 PE=1 SV=2 |
| Q6NYC8\|PPR18_HUMAN | 124.46 | 67943 | Phostensin OS=Homo sapiens OX=9606 GN=PPP1R18 PE=1 SV=1 |
| P61163\|ACTZ_HUMAN | 124.44 | 42614 | Alpha-centractin OS=Homo sapiens OX=9606 GN=ACTR1A PE=1 SV=1 |
| O00231\|PSD11_HUMAN | 124.3 | 47464 | 26S proteasome non-ATPase regulatory subunit 11 OS=Homo sapiens OX=9606 GN=PSMD11 PE=1 SV=3 |
| P23258\|TBG1_HUMAN | 124.12 | 51170 | Tubulin gamma-1 chain OS=Homo sapiens OX=9606 GN=TUBG1 PE=1 SV=2 |
| Q99832\|TCPH_HUMAN | 124.01 | 59367 | T-complex protein 1 subunit eta OS=Homo sapiens OX=9606 GN=CCT7 PE=1 SV=2 |
| P00352\|AL1A1_HUMAN | 123.27 | 54862 | Retinal dehydrogenase 1 OS=Homo sapiens OX=9606 GN=ALDH1A1 PE=1 SV=2 |
| Q15084\|PDIA6_HUMAN | 122.84 | 48121 | Protein disulfide-isomerase A6 OS=Homo sapiens OX=9606 GN=PDIA6 PE=1 SV=1 |
| O94929\|ABLM3_HUMAN | 122.82 | 77802 | Actin-binding LIM protein 3 OS=Homo sapiens OX=9606 GN=ABLIM3 PE=1 SV=3 |
| Q96EE3\|SEH1_HUMAN | 122.71 | 39649 | Nucleoporin SEH1 OS=Homo sapiens OX=9606 GN=SEH1L PE=1 SV=3 |
| P33176\|KINH_HUMAN | 122.63 | 109685 | Kinesin-1 heavy chain OS=Homo sapiens OX=9606 GN=KIF5B PE=1 SV=1 |
| P55072\|TERA_HUMAN | 122.03 | 89322 | Transitional endoplasmic reticulum ATPase OS=Homo sapiens OX=9606 GN=VCP PE=1 SV=4 |
| Q9BZH6\|WDR11_HUMAN | 121.92 | 136685 | WD repeat-containing protein 11 OS=Homo sapiens OX=9606 GN=WDR11 PE=1 SV=1 |
| P57740\|NU107_HUMAN | 121.83 | 106374 | Nuclear pore complex protein Nup107 OS=Homo sapiens OX=9606 GN=NUP107 PE=1 SV=1 |
| O43396\|TXNL1_HUMAN | 121.78 | 32251 | Thioredoxin-like protein 1 OS=Homo sapiens OX=9606 GN=TXNL1 PE=1 SV=3 |
| P08579\|RU2B_HUMAN | 121.76 | 25486 | U2 small nuclear ribonucleoprotein B'' OS=Homo sapiens OX=9606 GN=SNRPB2 PE=1 SV=1 |
| Q7Z2W4\|ZCCHV_HUMAN | 121.5 | 101431 | Zinc finger CCCH-type antiviral protein 1 OS=Homo sapiens OX=9606 GN=ZC3HAV1 PE=1 SV=3 |
| Q13724\|MOGS_HUMAN | 121.48 | 91918 | Mannosyl-oligosaccharide glucosidase OS=Homo sapiens OX=9606 GN=MOGS PE=1 SV=5 |
| Q7Z7K6\|CENPV_HUMAN | 121.3 | 29946 | Centromere protein V OS=Homo sapiens OX=9606 GN=CENPV PE=1 SV=1 |
| Q9Y3D7\|TIM16_HUMAN | 121.11 | 13825 | Mitochondrial import inner membrane translocase subunit TIM16 OS=Homo sapiens OX=9606 GN=PAM16 PE=1 SV=2 |
| Q08397\|LOXL1_HUMAN | 120.86 | 63110 | Lysyl oxidase homolog 1 OS=Homo sapiens OX=9606 GN=LOXL1 PE=1 SV=2 |
| Q14011\|CIRBP_HUMAN | 120.78 | 18648 | Cold-inducible RNA-binding protein OS=Homo sapiens OX=9606 GN=CIRBP PE=1 SV=1 |
| O95400\|CD2B2_HUMAN | 120.53 | 37646 | CD2 antigen cytoplasmic tail-binding protein 2 OS=Homo sapiens OX=9606 GN=CD2BP2 PE=1 SV=1 |
| P28289\|TMOD1_HUMAN | 120.13 | 40569 | Tropomodulin-1 OS=Homo sapiens OX=9606 GN=TMOD1 PE=1 SV=1 |
| Q13492\|PICAL_HUMAN | 120.13 | 70755 | Phosphatidylinositol-binding clathrin assembly protein OS=Homo sapiens OX=9606 GN=PICALM PE=1 SV=2 |
| P17980\|PRS6A_HUMAN | 119.24 | 49204 | 26S proteasome regulatory subunit 6A OS=Homo sapiens OX=9606 GN=PSMC3 PE=1 SV=3 |
| P05388\|RLA0_HUMAN | 119.23 | 34274 | 60S acidic ribosomal protein P0 OS=Homo sapiens OX=9606 GN=RPLP0 PE=1 SV=1 |
| Q13162\|PRDX4_HUMAN | 118.91 | 30540 | Peroxiredoxin-4 OS=Homo sapiens OX=9606 GN=PRDX4 PE=1 SV=1 |
| O15116\|LSM1_HUMAN | 118.79 | 15179 | U6 snRNA-associated Sm-like protein LSm1 OS=Homo sapiens OX=9606 GN=LSM1 PE=1 SV=1 |
| Q9BWJ5\|SF3B5_HUMAN | 118.71 | 10135 | Splicing factor 3B subunit 5 OS=Homo sapiens OX=9606 GN=SF3B5 PE=1 SV=1 |
| P81605\|DCD_HUMAN | 118.34 | 11284 | Dermcidin OS=Homo sapiens OX=9606 GN=DCD PE=1 SV=2 |
| Q3MHD2\|LSM12_HUMAN | 118.29 | 21701 | Protein LSM12 homolog OS=Homo sapiens OX=9606 GN=LSM12 PE=1 SV=2 |
| P62273\|RS29_HUMAN | 118.09 | 6677 | 40S ribosomal protein S29 OS=Homo sapiens OX=9606 GN=RPS29 PE=1 SV=2 |
| Q14980\|NUMA1_HUMAN | 118.05 | 238257 | Nuclear mitotic apparatus protein 1 OS=Homo sapiens OX=9606 GN=NUMA1 PE=1 SV=2 |
| Q9Y2A7\|NCKP1_HUMAN | 118.03 | 128790 | Nck-associated protein 1 OS=Homo sapiens OX=9606 GN=NCKAP1 PE=1 SV=1 |
| Q13045\|FLII_HUMAN | 117.97 | 144751 | Protein flightless-1 homolog OS=Homo sapiens OX=9606 GN=FLII PE=1 SV=2 |
| P29966\|MARCS_HUMAN | 117.8 | 31555 | Myristoylated alanine-rich C-kinase substrate OS=Homo sapiens OX=9606 GN=MARCKS PE=1 SV=4 |
| P14543\|NID1_HUMAN | 117.78 | 136377 | Nidogen-1 OS=Homo sapiens OX=9606 GN=NID1 PE=1 SV=3 |
| Q96P63\|SPB12_HUMAN | 117.27 | 46276 | Serpin B12 OS=Homo sapiens OX=9606 GN=SERPINB12 PE=1 SV=1 |
| Q9BZL4\|PP12C_HUMAN | 117.26 | 84881 | Protein phosphatase 1 regulatory subunit 12C OS=Homo sapiens OX=9606 GN=PPP1R12C PE=1 SV=1 |
| Q92917\|GPKOW_HUMAN | 117.1 | 52229 | G-patch domain and KOW motifs-containing protein OS=Homo sapiens OX=9606 GN=GPKOW PE=1 SV=2 |
| O00571\|DDX3X_HUMAN | 116.92 | 73244 | ATP-dependent RNA helicase DDX3X OS=Homo sapiens OX=9606 GN=DDX3X PE=1 SV=3 |
| P20618\|PSB1_HUMAN | 116.65 | 26489 | Proteasome subunit beta type-1 OS=Homo sapiens OX=9606 GN=PSMB1 PE=1 SV=2 |
| P62877\|RBX1_HUMAN | 116.26 | 12274 | E3 ubiquitin-protein ligase RBX1 OS=Homo sapiens OX=9606 GN=RBX1 PE=1 SV=1 |
| Q13011\|ECH1_HUMAN | 116.15 | 35816 | Delta(3 5)-Delta(2 4)-dienoyl-CoA isomerase mitochondrial OS=Homo sapiens OX=9606 GN=ECH1 PE=1 SV=2 |
| Q9Y5J9\|TIM8B_HUMAN | 115.71 | 9344 | Mitochondrial import inner membrane translocase subunit Tim8 B OS=Homo sapiens OX=9606 GN=TIMM8B PE=1 SV=1 |
| Q99729\|ROAA_HUMAN | 115.62 | 36225 | Heterogeneous nuclear ribonucleoprotein A/B OS=Homo sapiens OX=9606 GN=HNRNPAB PE=1 SV=2 |
| P60842\|IF4A1_HUMAN | 115.39 | 46154 | Eukaryotic initiation factor 4A-I OS=Homo sapiens OX=9606 GN=EIF4A1 PE=1 SV=1 |
| O15145\|ARPC3_HUMAN | 115.35 | 20547 | Actin-related protein 2/3 complex subunit 3 OS=Homo sapiens OX=9606 GN=ARPC3 PE=1 SV=3 |
| Q15785\|TOM34_HUMAN | 115.04 | 34559 | Mitochondrial import receptor subunit TOM34 OS=Homo sapiens OX=9606 GN=TOMM34 PE=1 SV=2 |
| P08758\|ANXA5_HUMAN | 114.91 | 35937 | Annexin A5 OS=Homo sapiens OX=9606 GN=ANXA5 PE=1 SV=2 |
| Q8NEV1\|CSK23_HUMAN | 114.68 | 45220 | Casein kinase II subunit alpha 3 OS=Homo sapiens OX=9606 GN=CSNK2A3 PE=1 SV=2 |
| P68400\|CSK21_HUMAN | 114.68 | 45144 | Casein kinase II subunit alpha OS=Homo sapiens OX=9606 GN=CSNK2A1 PE=1 SV=1 |
| Q08378\|GOGA3_HUMAN | 114.56 | 167354 | Golgin subfamily A member 3 OS=Homo sapiens OX=9606 GN=GOLGA3 PE=1 SV=2 |
| Q14444\|CAPR1_HUMAN | 114.19 | 78366 | Caprin-1 OS=Homo sapiens OX=9606 GN=CAPRIN1 PE=1 SV=2 |
| P20674\|COX5A_HUMAN | 114.19 | 16762 | Cytochrome c oxidase subunit 5A mitochondrial OS=Homo sapiens OX=9606 GN=COX5A PE=1 SV=2 |
| P40222\|TXLNA_HUMAN | 113.89 | 61891 | Alpha-taxilin OS=Homo sapiens OX=9606 GN=TXLNA PE=1 SV=3 |
| P22392\|NDKB_HUMAN | 113.85 | 17298 | Nucleoside diphosphate kinase B OS=Homo sapiens OX=9606 GN=NME2 PE=1 SV=1 |
| P15531\|NDKA_HUMAN | 113.85 | 17149 | Nucleoside diphosphate kinase A OS=Homo sapiens OX=9606 GN=NME1 PE=1 SV=1 |
| Q92747\|ARC1A_HUMAN | 113.8 | 41569 | Actin-related protein 2/3 complex subunit 1A OS=Homo sapiens OX=9606 GN=ARPC1A PE=2 SV=2 |
| Q6PK04\|CC137_HUMAN | 113.76 | 33231 | Coiled-coil domain-containing protein 137 OS=Homo sapiens OX=9606 GN=CCDC137 PE=1 SV=1 |
| Q7Z3B4\|NUP54_HUMAN | 113.63 | 55436 | Nucleoporin p54 OS=Homo sapiens OX=9606 GN=NUP54 PE=1 SV=2 |
| Q96QD9\|UIF_HUMAN | 113.57 | 35818 | UAP56-interacting factor OS=Homo sapiens OX=9606 GN=FYTTD1 PE=1 SV=3 |
| Q04837\|SSBP_HUMAN | 112.74 | 17260 | Single-stranded DNA-binding protein mitochondrial OS=Homo sapiens OX=9606 GN=SSBP1 PE=1 SV=1 |
| Q8N3V7\|SYNPO_HUMAN | 112.63 | 99463 | Synaptopodin OS=Homo sapiens OX=9606 GN=SYNPO PE=1 SV=2 |
| P62899\|RL31_HUMAN | 112.3 | 14463 | 60S ribosomal protein L31 OS=Homo sapiens OX=9606 GN=RPL31 PE=1 SV=1 |
| O75323\|NIPS2_HUMAN | 112.27 | 33743 | Protein NipSnap homolog 2 OS=Homo sapiens OX=9606 GN=NIPSNAP2 PE=1 SV=1 |
| Q12874\|SF3A3_HUMAN | 112.12 | 58849 | Splicing factor 3A subunit 3 OS=Homo sapiens OX=9606 GN=SF3A3 PE=1 SV=1 |
| P49368\|TCPG_HUMAN | 112.06 | 60534 | T-complex protein 1 subunit gamma OS=Homo sapiens OX=9606 GN=CCT3 PE=1 SV=4 |
| P62841\|RS15_HUMAN | 111.9 | 17040 | 40S ribosomal protein S15 OS=Homo sapiens OX=9606 GN=RPS15 PE=1 SV=2 |
| Q86UE4\|LYRIC_HUMAN | 111.5 | 63837 | Protein LYRIC OS=Homo sapiens OX=9606 GN=MTDH PE=1 SV=2 |
| Q9Y4I1\|MYO5A_HUMAN | 111.32 | 215403 | Unconventional myosin-Va OS=Homo sapiens OX=9606 GN=MYO5A PE=1 SV=2 |
| P98179\|RBM3_HUMAN | 111.23 | 17170 | RNA-binding protein 3 OS=Homo sapiens OX=9606 GN=RBM3 PE=1 SV=1 |
| Q9NR50\|EI2BG_HUMAN | 110.97 | 50240 | Translation initiation factor eIF-2B subunit gamma OS=Homo sapiens OX=9606 GN=EIF2B3 PE=1 SV=1 |
| O75489\|NDUS3_HUMAN | 110.63 | 30242 | NADH dehydrogenase [ubiquinone] iron-sulfur protein 3 mitochondrial OS=Homo sapiens OX=9606 GN=NDUFS3 PE=1 SV=1 |
| P52292\|IMA1_HUMAN | 110.52 | 57862 | Importin subunit alpha-1 OS=Homo sapiens OX=9606 GN=KPNA2 PE=1 SV=1 |
| Q9H444\|CHM4B_HUMAN | 110.2 | 24950 | Charged multivesicular body protein 4b OS=Homo sapiens OX=9606 GN=CHMP4B PE=1 SV=1 |
| P36578\|RL4_HUMAN | 110.15 | 47697 | 60S ribosomal protein L4 OS=Homo sapiens OX=9606 GN=RPL4 PE=1 SV=5 |
| Q96N67\|DOCK7_HUMAN | 109.89 | 242558 | Dedicator of cytokinesis protein 7 OS=Homo sapiens OX=9606 GN=DOCK7 PE=1 SV=4 |
| P11234\|RALB_HUMAN | 109.85 | 23409 | Ras-related protein Ral-B OS=Homo sapiens OX=9606 GN=RALB PE=1 SV=1 |
| P35221\|CTNA1_HUMAN | 109.32 | 100071 | Catenin alpha-1 OS=Homo sapiens OX=9606 GN=CTNNA1 PE=1 SV=1 |
| Q15691\|MARE1_HUMAN | 109.22 | 29999 | Microtubule-associated protein RP/EB family member 1 OS=Homo sapiens OX=9606 GN=MAPRE1 PE=1 SV=3 |
| Q9NXV6\|CARF_HUMAN | 108.65 | 61125 | CDKN2A-interacting protein OS=Homo sapiens OX=9606 GN=CDKN2AIP PE=1 SV=3 |
| Q92973\|TNPO1_HUMAN | 108.58 | 102355 | Transportin-1 OS=Homo sapiens OX=9606 GN=TNPO1 PE=1 SV=2 |
| P00338\|LDHA_HUMAN | 107.09 | 36689 | L-lactate dehydrogenase A chain OS=Homo sapiens OX=9606 GN=LDHA PE=1 SV=2 |
| P12236\|ADT3_HUMAN | 107.08 | 32866 | ADP/ATP translocase 3 OS=Homo sapiens OX=9606 GN=SLC25A6 PE=1 SV=4 |
| Q9Y3F4\|STRAP_HUMAN | 106.93 | 38438 | Serine-threonine kinase receptor-associated protein OS=Homo sapiens OX=9606 GN=STRAP PE=1 SV=1 |
| Q9Y333\|LSM2_HUMAN | 106.5 | 10835 | U6 snRNA-associated Sm-like protein LSm2 OS=Homo sapiens OX=9606 GN=LSM2 PE=1 SV=1 |
| Q9UII2\|ATIF1_HUMAN | 106.37 | 12249 | ATPase inhibitor mitochondrial OS=Homo sapiens OX=9606 GN=ATP5IF1 PE=1 SV=1 |
| Q71UM5\|RS27L_HUMAN | 105.92 | 9477 | 40S ribosomal protein S27-like OS=Homo sapiens OX=9606 GN=RPS27L PE=1 SV=3 |
| O00479\|HMGN4_HUMAN | 105.7 | 9539 | High mobility group nucleosome-binding domain-containing protein 4 OS=Homo sapiens OX=9606 GN=HMGN4 PE=1 SV=3 |
| Q9BSD7\|NTPCR_HUMAN | 105.12 | 20713 | Cancer-related nucleoside-triphosphatase OS=Homo sapiens OX=9606 GN=NTPCR PE=1 SV=1 |
| P46060\|RAGP1_HUMAN | 105.1 | 63542 | Ran GTPase-activating protein 1 OS=Homo sapiens OX=9606 GN=RANGAP1 PE=1 SV=1 |
| E9PRG8\|CK098_HUMAN | 104.83 | 14234 | Uncharacterized protein C11orf98 OS=Homo sapiens OX=9606 GN=C11orf98 PE=4 SV=2 |
| P09497\|CLCB_HUMAN | 104.54 | 25190 | Clathrin light chain B OS=Homo sapiens OX=9606 GN=CLTB PE=1 SV=1 |
| O14974\|MYPT1_HUMAN | 104.02 | 115281 | Protein phosphatase 1 regulatory subunit 12A OS=Homo sapiens OX=9606 GN=PPP1R12A PE=1 SV=1 |
| P61587\|RND3_HUMAN | 103.43 | 27368 | Rho-related GTP-binding protein RhoE OS=Homo sapiens OX=9606 GN=RND3 PE=1 SV=1 |
| O43776\|SYNC_HUMAN | 103.38 | 62943 | Asparagine--tRNA ligase cytoplasmic OS=Homo sapiens OX=9606 GN=NARS PE=1 SV=1 |
| Q8TAF3\|WDR48_HUMAN | 103.14 | 76211 | WD repeat-containing protein 48 OS=Homo sapiens OX=9606 GN=WDR48 PE=1 SV=1 |
| Q6P582\|MZT2A_HUMAN | 102.67 | 16221 | Mitotic-spindle organizing protein 2A OS=Homo sapiens OX=9606 GN=MZT2A PE=1 SV=2 |
| Q15434\|RBMS2_HUMAN | 102.15 | 43959 | RNA-binding motif single-stranded-interacting protein 2 OS=Homo sapiens OX=9606 GN=RBMS2 PE=1 SV=1 |
| Q02878\|RL6_HUMAN | 101.84 | 32728 | 60S ribosomal protein L6 OS=Homo sapiens OX=9606 GN=RPL6 PE=1 SV=3 |
| Q96BP2\|CHCH1_HUMAN | 101.56 | 13475 | Coiled-coil-helix-coiled-coil-helix domain-containing protein 1 OS=Homo sapiens OX=9606 GN=CHCHD1 PE=1 SV=1 |
| P21796\|VDAC1_HUMAN | 101.49 | 30773 | Voltage-dependent anion-selective channel protein 1 OS=Homo sapiens OX=9606 GN=VDAC1 PE=1 SV=2 |
| O43237\|DC1L2_HUMAN | 101.48 | 54099 | Cytoplasmic dynein 1 light intermediate chain 2 OS=Homo sapiens OX=9606 GN=DYNC1LI2 PE=1 SV=1 |
| Q69YQ0\|CYTSA_HUMAN | 100.75 | 124602 | Cytospin-A OS=Homo sapiens OX=9606 GN=SPECC1L PE=1 SV=2 |
| Q9GZZ1\|NAA50_HUMAN | 100.64 | 19398 | N-alpha-acetyltransferase 50 OS=Homo sapiens OX=9606 GN=NAA50 PE=1 SV=1 |
| Q13148\|TADBP_HUMAN | 100.54 | 44740 | TAR DNA-binding protein 43 OS=Homo sapiens OX=9606 GN=TARDBP PE=1 SV=1 |
| O95295\|SNAPN_HUMAN | 100.09 | 14874 | SNARE-associated protein Snapin OS=Homo sapiens OX=9606 GN=SNAPIN PE=1 SV=1 |
| Q99436\|PSB7_HUMAN | 99.57 | 29965 | Proteasome subunit beta type-7 OS=Homo sapiens OX=9606 GN=PSMB7 PE=1 SV=1 |
| P49770\|EI2BB_HUMAN | 99.53 | 38990 | Translation initiation factor eIF-2B subunit beta OS=Homo sapiens OX=9606 GN=EIF2B2 PE=1 SV=3 |
| Q99622\|C10_HUMAN | 99.12 | 13178 | Protein C10 OS=Homo sapiens OX=9606 GN=C12orf57 PE=1 SV=1 |
| P25788\|PSA3_HUMAN | 98.85 | 28433 | Proteasome subunit alpha type-3 OS=Homo sapiens OX=9606 GN=PSMA3 PE=1 SV=2 |
| P80723\|BASP1_HUMAN | 98.82 | 22693 | Brain acid soluble protein 1 OS=Homo sapiens OX=9606 GN=BASP1 PE=1 SV=2 |
| Q13835\|PKP1_HUMAN | 98.67 | 82861 | Plakophilin-1 OS=Homo sapiens OX=9606 GN=PKP1 PE=1 SV=2 |
| O60220\|TIM8A_HUMAN | 98.27 | 10998 | Mitochondrial import inner membrane translocase subunit Tim8 A OS=Homo sapiens OX=9606 GN=TIMM8A PE=1 SV=1 |
| O14497\|ARI1A_HUMAN | 97.45 | 242042 | AT-rich interactive domain-containing protein 1A OS=Homo sapiens OX=9606 GN=ARID1A PE=1 SV=3 |
| P43246\|MSH2_HUMAN | 97.18 | 104743 | DNA mismatch repair protein Msh2 OS=Homo sapiens OX=9606 GN=MSH2 PE=1 SV=1 |
| Q96JJ3\|ELMO2_HUMAN | 97.02 | 82615 | Engulfment and cell motility protein 2 OS=Homo sapiens OX=9606 GN=ELMO2 PE=1 SV=2 |
| Q9UNS2\|CSN3_HUMAN | 96.83 | 47873 | COP9 signalosome complex subunit 3 OS=Homo sapiens OX=9606 GN=COPS3 PE=1 SV=3 |
| Q96C36\|P5CR2_HUMAN | 96.8 | 33637 | Pyrroline-5-carboxylate reductase 2 OS=Homo sapiens OX=9606 GN=PYCR2 PE=1 SV=1 |
| Q9UMY1\|NOL7_HUMAN | 96.64 | 29426 | Nucleolar protein 7 OS=Homo sapiens OX=9606 GN=NOL7 PE=1 SV=2 |
| Q01518\|CAP1_HUMAN | 96.62 | 51902 | Adenylyl cyclase-associated protein 1 OS=Homo sapiens OX=9606 GN=CAP1 PE=1 SV=5 |
| Q92769\|HDAC2_HUMAN | 96.62 | 55364 | Histone deacetylase 2 OS=Homo sapiens OX=9606 GN=HDAC2 PE=1 SV=2 |
| Q13547\|HDAC1_HUMAN | 96.62 | 55103 | Histone deacetylase 1 OS=Homo sapiens OX=9606 GN=HDAC1 PE=1 SV=1 |
| P61421\|VA0D1_HUMAN | 96.45 | 40329 | V-type proton ATPase subunit d 1 OS=Homo sapiens OX=9606 GN=ATP6V0D1 PE=1 SV=1 |
| P13639\|EF2_HUMAN | 96.26 | 95338 | Elongation factor 2 OS=Homo sapiens OX=9606 GN=EEF2 PE=1 SV=4 |
| Q9NX40\|OCAD1_HUMAN | 96.03 | 27626 | OCIA domain-containing protein 1 OS=Homo sapiens OX=9606 GN=OCIAD1 PE=1 SV=1 |
| Q99439\|CNN2_HUMAN | 95.95 | 33697 | Calponin-2 OS=Homo sapiens OX=9606 GN=CNN2 PE=1 SV=4 |
| Q9BU61\|NDUF3_HUMAN | 95.76 | 20350 | NADH dehydrogenase [ubiquinone] 1 alpha subcomplex assembly factor 3 OS=Homo sapiens OX=9606 GN=NDUFAF3 PE=1 SV=1 |
| P63220\|RS21_HUMAN | 95.71 | 9111 | 40S ribosomal protein S21 OS=Homo sapiens OX=9606 GN=RPS21 PE=1 SV=1 |
| P62306\|RUXF_HUMAN | 95.54 | 9725 | Small nuclear ribonucleoprotein F OS=Homo sapiens OX=9606 GN=SNRPF PE=1 SV=1 |
| Q53SF7\|COBL1_HUMAN | 95.48 | 123868 | Cordon-bleu protein-like 1 OS=Homo sapiens OX=9606 GN=COBLL1 PE=1 SV=3 |
| P48960\|CD97_HUMAN | 94.44 | 91869 | CD97 antigen OS=Homo sapiens OX=9606 GN=CD97 PE=1 SV=4 |
| Q9UQN3\|CHM2B_HUMAN | 94.33 | 23907 | Charged multivesicular body protein 2b OS=Homo sapiens OX=9606 GN=CHMP2B PE=1 SV=1 |
| P05089\|ARGI1_HUMAN | 93.83 | 34735 | Arginase-1 OS=Homo sapiens OX=9606 GN=ARG1 PE=1 SV=2 |
| P56385\|ATP5I_HUMAN | 93.73 | 7933 | ATP synthase subunit e mitochondrial OS=Homo sapiens OX=9606 GN=ATP5ME PE=1 SV=2 |
| P21283\|VATC1_HUMAN | 93.48 | 43942 | V-type proton ATPase subunit C 1 OS=Homo sapiens OX=9606 GN=ATP6V1C1 PE=1 SV=4 |
| Q8NFH3\|NUP43_HUMAN | 93.22 | 42151 | Nucleoporin Nup43 OS=Homo sapiens OX=9606 GN=NUP43 PE=1 SV=1 |
| Q9ULC4\|MCTS1_HUMAN | 93.17 | 20555 | Malignant T-cell-amplified sequence 1 OS=Homo sapiens OX=9606 GN=MCTS1 PE=1 SV=1 |
| P28072\|PSB6_HUMAN | 93.06 | 25358 | Proteasome subunit beta type-6 OS=Homo sapiens OX=9606 GN=PSMB6 PE=1 SV=4 |
| P06703\|S10A6_HUMAN | 93.04 | 10180 | Protein S100-A6 OS=Homo sapiens OX=9606 GN=S100A6 PE=1 SV=1 |
| P09874\|PARP1_HUMAN | 92.98 | 113084 | Poly [ADP-ribose] polymerase 1 OS=Homo sapiens OX=9606 GN=PARP1 PE=1 SV=4 |
| Q6YHK3\|CD109_HUMAN | 92.68 | 161688 | CD109 antigen OS=Homo sapiens OX=9606 GN=CD109 PE=1 SV=2 |
| Q9H6F5\|CCD86_HUMAN | 92.67 | 40236 | Coiled-coil domain-containing protein 86 OS=Homo sapiens OX=9606 GN=CCDC86 PE=1 SV=1 |
| Q14112\|NID2_HUMAN | 92.66 | 151254 | Nidogen-2 OS=Homo sapiens OX=9606 GN=NID2 PE=1 SV=3 |
| Q8IVM0\|CCD50_HUMAN | 92.38 | 35822 | Coiled-coil domain-containing protein 50 OS=Homo sapiens OX=9606 GN=CCDC50 PE=1 SV=1 |
| Q16666\|IF16_HUMAN | 92.28 | 88256 | Gamma-interferon-inducible protein 16 OS=Homo sapiens OX=9606 GN=IFI16 PE=1 SV=3 |
| P84098\|RL19_HUMAN | 92.27 | 23466 | 60S ribosomal protein L19 OS=Homo sapiens OX=9606 GN=RPL19 PE=1 SV=1 |
| P07942\|LAMB1_HUMAN | 91.31 | 198037 | Laminin subunit beta-1 OS=Homo sapiens OX=9606 GN=LAMB1 PE=1 SV=2 |
| P27105\|STOM_HUMAN | 90.92 | 31731 | Erythrocyte band 7 integral membrane protein OS=Homo sapiens OX=9606 GN=STOM PE=1 SV=3 |
| Q99584\|S10AD_HUMAN | 90.84 | 11471 | Protein S100-A13 OS=Homo sapiens OX=9606 GN=S100A13 PE=1 SV=1 |
| Q9NSD9\|SYFB_HUMAN | 90.69 | 66116 | Phenylalanine--tRNA ligase beta subunit OS=Homo sapiens OX=9606 GN=FARSB PE=1 SV=3 |
| P53396\|ACLY_HUMAN | 90.63 | 120839 | ATP-citrate synthase OS=Homo sapiens OX=9606 GN=ACLY PE=1 SV=3 |
| P27635\|RL10_HUMAN | 90.53 | 24604 | 60S ribosomal protein L10 OS=Homo sapiens OX=9606 GN=RPL10 PE=1 SV=4 |
| Q96L21\|RL10L_HUMAN | 90.53 | 24519 | 60S ribosomal protein L10-like OS=Homo sapiens OX=9606 GN=RPL10L PE=1 SV=3 |
| P42696\|RBM34_HUMAN | 89.87 | 48565 | RNA-binding protein 34 OS=Homo sapiens OX=9606 GN=RBM34 PE=1 SV=2 |
| P18669\|PGAM1_HUMAN | 89.79 | 28804 | Phosphoglycerate mutase 1 OS=Homo sapiens OX=9606 GN=PGAM1 PE=1 SV=2 |
| Q9UHX1\|PUF60_HUMAN | 89.42 | 59876 | Poly(U)-binding-splicing factor PUF60 OS=Homo sapiens OX=9606 GN=PUF60 PE=1 SV=1 |
| Q9Y295\|DRG1_HUMAN | 89.4 | 40542 | Developmentally-regulated GTP-binding protein 1 OS=Homo sapiens OX=9606 GN=DRG1 PE=1 SV=1 |
| P63162\|RSMN_HUMAN | 89.27 | 24614 | Small nuclear ribonucleoprotein-associated protein N OS=Homo sapiens OX=9606 GN=SNRPN PE=1 SV=1 |
| P14678\|RSMB_HUMAN | 89.27 | 24610 | Small nuclear ribonucleoprotein-associated proteins B and B' OS=Homo sapiens OX=9606 GN=SNRPB PE=1 SV=2 |
| P22695\|QCR2_HUMAN | 89.13 | 48443 | Cytochrome b-c1 complex subunit 2 mitochondrial OS=Homo sapiens OX=9606 GN=UQCRC2 PE=1 SV=3 |
| Q9UI09\|NDUAC_HUMAN | 88.92 | 17114 | NADH dehydrogenase [ubiquinone] 1 alpha subcomplex subunit 12 OS=Homo sapiens OX=9606 GN=NDUFA12 PE=1 SV=1 |
| Q9UQ35\|SRRM2_HUMAN | 88.89 | 299616 | Serine/arginine repetitive matrix protein 2 OS=Homo sapiens OX=9606 GN=SRRM2 PE=1 SV=2 |
| O14639\|ABLM1_HUMAN | 88.8 | 87688 | Actin-binding LIM protein 1 OS=Homo sapiens OX=9606 GN=ABLIM1 PE=1 SV=3 |
| Q13884\|SNTB1_HUMAN | 88.29 | 58061 | Beta-1-syntrophin OS=Homo sapiens OX=9606 GN=SNTB1 PE=1 SV=3 |
| Q8N8S7\|ENAH_HUMAN | 88.14 | 66510 | Protein enabled homolog OS=Homo sapiens OX=9606 GN=ENAH PE=1 SV=2 |
| P49411\|EFTU_HUMAN | 87.58 | 49542 | Elongation factor Tu mitochondrial OS=Homo sapiens OX=9606 GN=TUFM PE=1 SV=2 |
| P08865\|RSSA_HUMAN | 87.31 | 32854 | 40S ribosomal protein SA OS=Homo sapiens OX=9606 GN=RPSA PE=1 SV=4 |
| Q9BRT6\|LLPH_HUMAN | 87.26 | 15225 | Protein LLP homolog OS=Homo sapiens OX=9606 GN=LLPH PE=1 SV=1 |
| P53621\|COPA_HUMAN | 87.2 | 138345 | Coatomer subunit alpha OS=Homo sapiens OX=9606 GN=COPA PE=1 SV=2 |
| P04899\|GNAI2_HUMAN | 87.13 | 40451 | Guanine nucleotide-binding protein G(i) subunit alpha-2 OS=Homo sapiens OX=9606 GN=GNAI2 PE=1 SV=3 |
| Q96B26\|EXOS8_HUMAN | 86.86 | 30040 | Exosome complex component RRP43 OS=Homo sapiens OX=9606 GN=EXOSC8 PE=1 SV=1 |
| P56537\|IF6_HUMAN | 86.41 | 26599 | Eukaryotic translation initiation factor 6 OS=Homo sapiens OX=9606 GN=EIF6 PE=1 SV=1 |
| Q9Y608\|LRRF2_HUMAN | 86.27 | 82171 | Leucine-rich repeat flightless-interacting protein 2 OS=Homo sapiens OX=9606 GN=LRRFIP2 PE=1 SV=1 |
| P17931\|LEG3_HUMAN | 86.16 | 26152 | Galectin-3 OS=Homo sapiens OX=9606 GN=LGALS3 PE=1 SV=5 |
| Q16555\|DPYL2_HUMAN | 86.07 | 62294 | Dihydropyrimidinase-related protein 2 OS=Homo sapiens OX=9606 GN=DPYSL2 PE=1 SV=1 |
| Q6ZUT6\|CCD9B_HUMAN | 85.76 | 57325 | Coiled-coil domain-containing protein 9B OS=Homo sapiens OX=9606 GN=CCDC9B PE=1 SV=1 |
| P61964\|WDR5_HUMAN | 85.44 | 36589 | WD repeat-containing protein 5 OS=Homo sapiens OX=9606 GN=WDR5 PE=1 SV=1 |
| P49207\|RL34_HUMAN | 85.28 | 13293 | 60S ribosomal protein L34 OS=Homo sapiens OX=9606 GN=RPL34 PE=1 SV=3 |
| P55265\|DSRAD_HUMAN | 85.24 | 136066 | Double-stranded RNA-specific adenosine deaminase OS=Homo sapiens OX=9606 GN=ADAR PE=1 SV=4 |
| P55268\|LAMB2_HUMAN | 85.01 | 195980 | Laminin subunit beta-2 OS=Homo sapiens OX=9606 GN=LAMB2 PE=1 SV=2 |
| Q86SQ0\|PHLB2_HUMAN | 84.87 | 142158 | Pleckstrin homology-like domain family B member 2 OS=Homo sapiens OX=9606 GN=PHLDB2 PE=1 SV=2 |
| Q15008\|PSMD6_HUMAN | 84.74 | 45531 | 26S proteasome non-ATPase regulatory subunit 6 OS=Homo sapiens OX=9606 GN=PSMD6 PE=1 SV=1 |
| Q9Y4K0\|LOXL2_HUMAN | 84.69 | 86725 | Lysyl oxidase homolog 2 OS=Homo sapiens OX=9606 GN=LOXL2 PE=1 SV=1 |
| P02461\|CO3A1_HUMAN | 84.43 | 138564 | Collagen alpha-1(III) chain OS=Homo sapiens OX=9606 GN=COL3A1 PE=1 SV=4 |
| Q12959\|DLG1_HUMAN | 84.33 | 100455 | Disks large homolog 1 OS=Homo sapiens OX=9606 GN=DLG1 PE=1 SV=2 |
| Q14232\|EI2BA_HUMAN | 84.28 | 33712 | Translation initiation factor eIF-2B subunit alpha OS=Homo sapiens OX=9606 GN=EIF2B1 PE=1 SV=1 |
| P20908\|CO5A1_HUMAN | 84.17 | 183559 | Collagen alpha-1(V) chain OS=Homo sapiens OX=9606 GN=COL5A1 PE=1 SV=3 |
| P49720\|PSB3_HUMAN | 84.02 | 22949 | Proteasome subunit beta type-3 OS=Homo sapiens OX=9606 GN=PSMB3 PE=1 SV=2 |
| P05023\|AT1A1_HUMAN | 83.89 | 112896 | Sodium/potassium-transporting ATPase subunit alpha-1 OS=Homo sapiens OX=9606 GN=ATP1A1 PE=1 SV=1 |
| Q9UHQ9\|NB5R1_HUMAN | 83.64 | 34095 | NADH-cytochrome b5 reductase 1 OS=Homo sapiens OX=9606 GN=CYB5R1 PE=1 SV=1 |
| P19823\|ITIH2_HUMAN | 83.43 | 106463 | Inter-alpha-trypsin inhibitor heavy chain H2 OS=Homo sapiens OX=9606 GN=ITIH2 PE=1 SV=2 |
| P68871\|HBB_HUMAN | 81.8 | 15998 | Hemoglobin subunit beta OS=Homo sapiens OX=9606 GN=HBB PE=1 SV=2 |
| O60869\|EDF1_HUMAN | 81.57 | 16369 | Endothelial differentiation-related factor 1 OS=Homo sapiens OX=9606 GN=EDF1 PE=1 SV=1 |
| O95777\|LSM8_HUMAN | 81.48 | 10403 | U6 snRNA-associated Sm-like protein LSm8 OS=Homo sapiens OX=9606 GN=LSM8 PE=1 SV=3 |
| Q92890\|UFD1_HUMAN | 80.76 | 34500 | Ubiquitin recognition factor in ER-associated degradation protein 1 OS=Homo sapiens OX=9606 GN=UFD1 PE=1 SV=3 |
| P41221\|WNT5A_HUMAN | 80.61 | 42340 | Protein Wnt-5a OS=Homo sapiens OX=9606 GN=WNT5A PE=1 SV=2 |
| Q9BUP0\|EFHD1_HUMAN | 80.59 | 26928 | EF-hand domain-containing protein D1 OS=Homo sapiens OX=9606 GN=EFHD1 PE=1 SV=1 |
| Q9P2J5\|SYLC_HUMAN | 80.17 | 134466 | Leucine--tRNA ligase cytoplasmic OS=Homo sapiens OX=9606 GN=LARS PE=1 SV=2 |
| O00746\|NDKM_HUMAN | 80 | 20659 | Nucleoside diphosphate kinase mitochondrial OS=Homo sapiens OX=9606 GN=NME4 PE=1 SV=1 |
| A8MWD9\|RUXGL_HUMAN | 79.16 | 8544 | Putative small nuclear ribonucleoprotein G-like protein 15 OS=Homo sapiens OX=9606 GN=SNRPGP15 PE=5 SV=2 |
| P62308\|RUXG_HUMAN | 79.16 | 8496 | Small nuclear ribonucleoprotein G OS=Homo sapiens OX=9606 GN=SNRPG PE=1 SV=1 |
| P23497\|SP100_HUMAN | 78.83 | 100417 | Nuclear autoantigen Sp-100 OS=Homo sapiens OX=9606 GN=SP100 PE=1 SV=3 |
| Q9UHV9\|PFD2_HUMAN | 78.82 | 16648 | Prefoldin subunit 2 OS=Homo sapiens OX=9606 GN=PFDN2 PE=1 SV=1 |
| P56556\|NDUA6_HUMAN | 78.81 | 15137 | NADH dehydrogenase [ubiquinone] 1 alpha subcomplex subunit 6 OS=Homo sapiens OX=9606 GN=NDUFA6 PE=1 SV=4 |
| P48047\|ATPO_HUMAN | 78.77 | 23277 | ATP synthase subunit O mitochondrial OS=Homo sapiens OX=9606 GN=ATP5PO PE=1 SV=1 |
| Q9BV38\|WDR18_HUMAN | 78.58 | 47405 | WD repeat-containing protein 18 OS=Homo sapiens OX=9606 GN=WDR18 PE=1 SV=2 |
| Q9H1A4\|APC1_HUMAN | 78.17 | 216498 | Anaphase-promoting complex subunit 1 OS=Homo sapiens OX=9606 GN=ANAPC1 PE=1 SV=1 |
| Q86YP4\|P66A_HUMAN | 77.09 | 68063 | Transcriptional repressor p66-alpha OS=Homo sapiens OX=9606 GN=GATAD2A PE=1 SV=1 |
| Q9Y678\|COPG1_HUMAN | 76.97 | 97718 | Coatomer subunit gamma-1 OS=Homo sapiens OX=9606 GN=COPG1 PE=1 SV=1 |
| Q969Q0\|RL36L_HUMAN | 76.94 | 12469 | 60S ribosomal protein L36a-like OS=Homo sapiens OX=9606 GN=RPL36AL PE=1 SV=3 |
| P41252\|SYIC_HUMAN | 76.73 | 144498 | Isoleucine--tRNA ligase cytoplasmic OS=Homo sapiens OX=9606 GN=IARS PE=1 SV=2 |
| O14734\|ACOT8_HUMAN | 76.7 | 35914 | Acyl-coenzyme A thioesterase 8 OS=Homo sapiens OX=9606 GN=ACOT8 PE=1 SV=1 |
| Q9UQ03\|COR2B_HUMAN | 76.4 | 54954 | Coronin-2B OS=Homo sapiens OX=9606 GN=CORO2B PE=1 SV=4 |
| Q9BVG4\|PBDC1_HUMAN | 76.12 | 26057 | Protein PBDC1 OS=Homo sapiens OX=9606 GN=PBDC1 PE=1 SV=1 |
| P49792\|RBP2_HUMAN | 75.62 | 358201 | E3 SUMO-protein ligase RanBP2 OS=Homo sapiens OX=9606 GN=RANBP2 PE=1 SV=2 |
| O43678\|NDUA2_HUMAN | 75.32 | 10922 | NADH dehydrogenase [ubiquinone] 1 alpha subcomplex subunit 2 OS=Homo sapiens OX=9606 GN=NDUFA2 PE=1 SV=3 |
| Q07020\|RL18_HUMAN | 74.94 | 21634 | 60S ribosomal protein L18 OS=Homo sapiens OX=9606 GN=RPL18 PE=1 SV=2 |
| O60341\|KDM1A_HUMAN | 74.53 | 92903 | Lysine-specific histone demethylase 1A OS=Homo sapiens OX=9606 GN=KDM1A PE=1 SV=2 |
| Q13185\|CBX3_HUMAN | 74.48 | 20811 | Chromobox protein homolog 3 OS=Homo sapiens OX=9606 GN=CBX3 PE=1 SV=4 |
| Q5VUJ6\|LRCH2_HUMAN | 74.01 | 84588 | Leucine-rich repeat and calponin homology domain-containing protein 2 OS=Homo sapiens OX=9606 GN=LRCH2 PE=2 SV=2 |
| O60684\|IMA7_HUMAN | 73.77 | 60030 | Importin subunit alpha-7 OS=Homo sapiens OX=9606 GN=KPNA6 PE=1 SV=1 |
| Q8WZ42\|TITIN_HUMAN | 73.71 | 3815922 | Titin OS=Homo sapiens OX=9606 GN=TTN PE=1 SV=4 |
| Q15417\|CNN3_HUMAN | 73.14 | 36414 | Calponin-3 OS=Homo sapiens OX=9606 GN=CNN3 PE=1 SV=1 |
| P01116\|RASK_HUMAN | 73.09 | 21656 | GTPase KRas OS=Homo sapiens OX=9606 GN=KRAS PE=1 SV=1 |
| P62701\|RS4X_HUMAN | 72.9 | 29598 | 40S ribosomal protein S4 X isoform OS=Homo sapiens OX=9606 GN=RPS4X PE=1 SV=2 |
| O43324\|MCA3_HUMAN | 72.83 | 19811 | Eukaryotic translation elongation factor 1 epsilon-1 OS=Homo sapiens OX=9606 GN=EEF1E1 PE=1 SV=1 |
| P08238\|HS90B_HUMAN | 72.66 | 83264 | Heat shock protein HSP 90-beta OS=Homo sapiens OX=9606 GN=HSP90AB1 PE=1 SV=4 |
| O75533\|SF3B1_HUMAN | 71.83 | 145830 | Splicing factor 3B subunit 1 OS=Homo sapiens OX=9606 GN=SF3B1 PE=1 SV=3 |
| Q9UNE7\|CHIP_HUMAN | 71.6 | 34856 | E3 ubiquitin-protein ligase CHIP OS=Homo sapiens OX=9606 GN=STUB1 PE=1 SV=2 |
| P51116\|FXR2_HUMAN | 71.01 | 74223 | Fragile X mental retardation syndrome-related protein 2 OS=Homo sapiens OX=9606 GN=FXR2 PE=1 SV=2 |
| P51114\|FXR1_HUMAN | 71.01 | 69721 | Fragile X mental retardation syndrome-related protein 1 OS=Homo sapiens OX=9606 GN=FXR1 PE=1 SV=3 |
| P12109\|CO6A1_HUMAN | 70.8 | 108529 | Collagen alpha-1(VI) chain OS=Homo sapiens OX=9606 GN=COL6A1 PE=1 SV=3 |
| Q99460\|PSMD1_HUMAN | 70.31 | 105836 | 26S proteasome non-ATPase regulatory subunit 1 OS=Homo sapiens OX=9606 GN=PSMD1 PE=1 SV=2 |
| Q6P1N0\|C2D1A_HUMAN | 69.91 | 104062 | Coiled-coil and C2 domain-containing protein 1A OS=Homo sapiens OX=9606 GN=CC2D1A PE=1 SV=1 |
| Q86Y79\|PTH_HUMAN | 69.74 | 22937 | Probable peptidyl-tRNA hydrolase OS=Homo sapiens OX=9606 GN=PTRH1 PE=1 SV=1 |
| Q9NSC5\|HOME3_HUMAN | 69.66 | 39836 | Homer protein homolog 3 OS=Homo sapiens OX=9606 GN=HOMER3 PE=1 SV=2 |
| Q15637\|SF01_HUMAN | 69.65 | 68330 | Splicing factor 1 OS=Homo sapiens OX=9606 GN=SF1 PE=1 SV=4 |
| Q14203\|DCTN1_HUMAN | 69.11 | 141694 | Dynactin subunit 1 OS=Homo sapiens OX=9606 GN=DCTN1 PE=1 SV=3 |
| Q92621\|NU205_HUMAN | 67.42 | 227919 | Nuclear pore complex protein Nup205 OS=Homo sapiens OX=9606 GN=NUP205 PE=1 SV=3 |
| Q08431\|MFGM_HUMAN | 67.26 | 43105 | Lactadherin OS=Homo sapiens OX=9606 GN=MFGE8 PE=1 SV=3 |
| Q5T749\|KPRP_HUMAN | 66.48 | 64136 | Keratinocyte proline-rich protein OS=Homo sapiens OX=9606 GN=KPRP PE=1 SV=1 |
| P35606\|COPB2_HUMAN | 66.33 | 102487 | Coatomer subunit beta' OS=Homo sapiens OX=9606 GN=COPB2 PE=1 SV=2 |
| P05997\|CO5A2_HUMAN | 66.23 | 144910 | Collagen alpha-2(V) chain OS=Homo sapiens OX=9606 GN=COL5A2 PE=1 SV=3 |
| Q86WB0\|NIPA_HUMAN | 66.11 | 55262 | Nuclear-interacting partner of ALK OS=Homo sapiens OX=9606 GN=ZC3HC1 PE=1 SV=1 |
| Q13144\|EI2BE_HUMAN | 65.5 | 80380 | Translation initiation factor eIF-2B subunit epsilon OS=Homo sapiens OX=9606 GN=EIF2B5 PE=1 SV=3 |
| Q9UKX7\|NUP50_HUMAN | 64.92 | 50144 | Nuclear pore complex protein Nup50 OS=Homo sapiens OX=9606 GN=NUP50 PE=1 SV=2 |
| Q9UEY8\|ADDG_HUMAN | 64.49 | 79155 | Gamma-adducin OS=Homo sapiens OX=9606 GN=ADD3 PE=1 SV=1 |
| Q9BSJ2\|GCP2_HUMAN | 64.43 | 102534 | Gamma-tubulin complex component 2 OS=Homo sapiens OX=9606 GN=TUBGCP2 PE=1 SV=2 |
| Q9NR99\|MXRA5_HUMAN | 64.34 | 312149 | Matrix-remodeling-associated protein 5 OS=Homo sapiens OX=9606 GN=MXRA5 PE=1 SV=3 |
| Q9BWM7\|SFXN3_HUMAN | 64.06 | 35503 | Sideroflexin-3 OS=Homo sapiens OX=9606 GN=SFXN3 PE=1 SV=3 |
| P62333\|PRS10_HUMAN | 63.62 | 44173 | 26S proteasome regulatory subunit 10B OS=Homo sapiens OX=9606 GN=PSMC6 PE=1 SV=1 |
| Q14847\|LASP1_HUMAN | 63.49 | 29717 | LIM and SH3 domain protein 1 OS=Homo sapiens OX=9606 GN=LASP1 PE=1 SV=2 |
| Q07157\|ZO1_HUMAN | 62.62 | 195457 | Tight junction protein ZO-1 OS=Homo sapiens OX=9606 GN=TJP1 PE=1 SV=3 |
| Q8WXH0\|SYNE2_HUMAN | 62.5 | 796457 | Nesprin-2 OS=Homo sapiens OX=9606 GN=SYNE2 PE=1 SV=3 |
| Q96CW5\|GCP3_HUMAN | 62.38 | 103571 | Gamma-tubulin complex component 3 OS=Homo sapiens OX=9606 GN=TUBGCP3 PE=1 SV=2 |
| Q5T1M5\|FKB15_HUMAN | 62.37 | 133630 | FK506-binding protein 15 OS=Homo sapiens OX=9606 GN=FKBP15 PE=1 SV=2 |
| Q9NUI1\|DECR2_HUMAN | 62.09 | 30778 | Peroxisomal 2 4-dienoyl-CoA reductase OS=Homo sapiens OX=9606 GN=DECR2 PE=1 SV=1 |
| P83111\|LACTB_HUMAN | 61.93 | 60694 | Serine beta-lactamase-like protein LACTB mitochondrial OS=Homo sapiens OX=9606 GN=LACTB PE=1 SV=2 |
| P0DI83\|NARR_HUMAN | 61.74 | 21118 | Ras-related protein Rab-34 isoform NARR OS=Homo sapiens OX=9606 GN=RAB34 PE=1 SV=1 |
| P50281\|MMP14_HUMAN | 61.52 | 65894 | Matrix metalloproteinase-14 OS=Homo sapiens OX=9606 GN=MMP14 PE=1 SV=3 |
| Q8NFW8\|NEUA_HUMAN | 60.78 | 48379 | N-acylneuraminate cytidylyltransferase OS=Homo sapiens OX=9606 GN=CMAS PE=1 SV=2 |
| P12259\|FA5_HUMAN | 60.74 | 251701 | Coagulation factor V OS=Homo sapiens OX=9606 GN=F5 PE=1 SV=4 |
| P78559\|MAP1A_HUMAN | 60.55 | 305484 | Microtubule-associated protein 1A OS=Homo sapiens OX=9606 GN=MAP1A PE=1 SV=6 |
| P20340\|RAB6A_HUMAN | 60.53 | 23593 | Ras-related protein Rab-6A OS=Homo sapiens OX=9606 GN=RAB6A PE=1 SV=3 |
| Q16576\|RBBP7_HUMAN | 60.16 | 47820 | Histone-binding protein RBBP7 OS=Homo sapiens OX=9606 GN=RBBP7 PE=1 SV=1 |
| O15460\|P4HA2_HUMAN | 60.05 | 60902 | Prolyl 4-hydroxylase subunit alpha-2 OS=Homo sapiens OX=9606 GN=P4HA2 PE=1 SV=1 |
| Q6ZU64\|CFA65_HUMAN | 60 | 217248 | Cilia- and flagella-associated protein 65 OS=Homo sapiens OX=9606 GN=CFAP65 PE=1 SV=2 |
| O00468\|AGRIN_HUMAN | 58.32 | 217318 | Agrin OS=Homo sapiens OX=9606 GN=AGRN PE=1 SV=6 |
| Q9Y285\|SYFA_HUMAN | 58.32 | 57564 | Phenylalanine--tRNA ligase alpha subunit OS=Homo sapiens OX=9606 GN=FARSA PE=1 SV=3 |
| Q6ZU15\|SEP14_HUMAN | 57.1 | 50025 | Septin-14 OS=Homo sapiens OX=9606 GN=SEPTIN14 PE=1 SV=2 |
| P02792\|FRIL_HUMAN | 56.24 | 20020 | Ferritin light chain OS=Homo sapiens OX=9606 GN=FTL PE=1 SV=2 |
| Q99653\|CHP1_HUMAN | 56.18 | 22456 | Calcineurin B homologous protein 1 OS=Homo sapiens OX=9606 GN=CHP1 PE=1 SV=3 |
| P27797\|CALR_HUMAN | 55.82 | 48142 | Calreticulin OS=Homo sapiens OX=9606 GN=CALR PE=1 SV=1 |
| Q9NTI2\|AT8A2_HUMAN | 55.8 | 133598 | Phospholipid-transporting ATPase IB OS=Homo sapiens OX=9606 GN=ATP8A2 PE=1 SV=3 |
| O43447\|PPIH_HUMAN | 55.27 | 19208 | Peptidyl-prolyl cis-trans isomerase H OS=Homo sapiens OX=9606 GN=PPIH PE=1 SV=1 |
| Q9Y305\|ACOT9_HUMAN | 55.25 | 49902 | Acyl-coenzyme A thioesterase 9 mitochondrial OS=Homo sapiens OX=9606 GN=ACOT9 PE=1 SV=2 |
| Q14573\|ITPR3_HUMAN | 54.66 | 304105 | Inositol 1 4 5-trisphosphate receptor type 3 OS=Homo sapiens OX=9606 GN=ITPR3 PE=1 SV=2 |
| Q9NZI8\|IF2B1_HUMAN | 54.15 | 63481 | Insulin-like growth factor 2 mRNA-binding protein 1 OS=Homo sapiens OX=9606 GN=IGF2BP1 PE=1 SV=2 |
| Q15517\|CDSN_HUMAN | 54.01 | 51522 | Corneodesmosin OS=Homo sapiens OX=9606 GN=CDSN PE=1 SV=3 |
| Q8N163\|CCAR2_HUMAN | 53.96 | 102902 | Cell cycle and apoptosis regulator protein 2 OS=Homo sapiens OX=9606 GN=CCAR2 PE=1 SV=2 |
| P49821\|NDUV1_HUMAN | 53.55 | 50817 | NADH dehydrogenase [ubiquinone] flavoprotein 1 mitochondrial OS=Homo sapiens OX=9606 GN=NDUFV1 PE=1 SV=4 |
| Q86V48\|LUZP1_HUMAN | 53.5 | 120275 | Leucine zipper protein 1 OS=Homo sapiens OX=9606 GN=LUZP1 PE=1 SV=2 |
| Q86WX3\|AROS_HUMAN | 53.27 | 15434 | Active regulator of SIRT1 OS=Homo sapiens OX=9606 GN=RPS19BP1 PE=1 SV=1 |
| Q96EY5\|MB12A_HUMAN | 53.14 | 28783 | Multivesicular body subunit 12A OS=Homo sapiens OX=9606 GN=MVB12A PE=1 SV=1 |
| Q9HBH5\|RDH14_HUMAN | 52.96 | 36865 | Retinol dehydrogenase 14 OS=Homo sapiens OX=9606 GN=RDH14 PE=1 SV=1 |
| P12273\|PIP_HUMAN | 52.9 | 16572 | Prolactin-inducible protein OS=Homo sapiens OX=9606 GN=PIP PE=1 SV=1 |
| O95793\|STAU1_HUMAN | 52.26 | 63182 | Double-stranded RNA-binding protein Staufen homolog 1 OS=Homo sapiens OX=9606 GN=STAU1 PE=1 SV=2 |
| O00560\|SDCB1_HUMAN | 52.17 | 32444 | Syntenin-1 OS=Homo sapiens OX=9606 GN=SDCBP PE=1 SV=1 |
| Q9NZM1\|MYOF_HUMAN | 51.87 | 234706 | Myoferlin OS=Homo sapiens OX=9606 GN=MYOF PE=1 SV=1 |
| Q9HD42\|CHM1A_HUMAN | 49.62 | 21703 | Charged multivesicular body protein 1a OS=Homo sapiens OX=9606 GN=CHMP1A PE=1 SV=1 |
| Q9BW19\|KIFC1_HUMAN | 49.34 | 73748 | Kinesin-like protein KIFC1 OS=Homo sapiens OX=9606 GN=KIFC1 PE=1 SV=2 |
| Q9P2B4\|CT2NL_HUMAN | 49.34 | 70158 | CTTNBP2 N-terminal-like protein OS=Homo sapiens OX=9606 GN=CTTNBP2NL PE=1 SV=2 |
| O00217\|NDUS8_HUMAN | 49.18 | 23705 | NADH dehydrogenase [ubiquinone] iron-sulfur protein 8 mitochondrial OS=Homo sapiens OX=9606 GN=NDUFS8 PE=1 SV=1 |
| O00469\|PLOD2_HUMAN | 49.17 | 84686 | Procollagen-lysine 2-oxoglutarate 5-dioxygenase 2 OS=Homo sapiens OX=9606 GN=PLOD2 PE=1 SV=2 |
| Q9NQT4\|EXOS5_HUMAN | 49.02 | 25249 | Exosome complex component RRP46 OS=Homo sapiens OX=9606 GN=EXOSC5 PE=1 SV=1 |
| Q16513\|PKN2_HUMAN | 48.51 | 112035 | Serine/threonine-protein kinase N2 OS=Homo sapiens OX=9606 GN=PKN2 PE=1 SV=1 |
| Q5SRE5\|NU188_HUMAN | 48.27 | 196041 | Nucleoporin NUP188 homolog OS=Homo sapiens OX=9606 GN=NUP188 PE=1 SV=1 |
| P46459\|NSF_HUMAN | 48.19 | 82594 | Vesicle-fusing ATPase OS=Homo sapiens OX=9606 GN=NSF PE=1 SV=3 |
| Q5T7W0\|ZN618_HUMAN | 47.93 | 104955 | Zinc finger protein 618 OS=Homo sapiens OX=9606 GN=ZNF618 PE=1 SV=1 |
| P29279\|CCN2_HUMAN | 46.99 | 38091 | CCN family member 2 OS=Homo sapiens OX=9606 GN=CCN2 PE=1 SV=2 |
| P60174\|TPIS_HUMAN | 46.97 | 30791 | Triosephosphate isomerase OS=Homo sapiens OX=9606 GN=TPI1 PE=1 SV=3 |
| Q9Y5M8\|SRPRB_HUMAN | 46.76 | 29702 | Signal recognition particle receptor subunit beta OS=Homo sapiens OX=9606 GN=SRPRB PE=1 SV=3 |
| Q9UMX5\|NENF_HUMAN | 46.21 | 18856 | Neudesin OS=Homo sapiens OX=9606 GN=NENF PE=1 SV=1 |
| Q92878\|RAD50_HUMAN | 45.56 | 153892 | DNA repair protein RAD50 OS=Homo sapiens OX=9606 GN=RAD50 PE=1 SV=1 |
| Q9P013\|CWC15_HUMAN | 45.38 | 26624 | Spliceosome-associated protein CWC15 homolog OS=Homo sapiens OX=9606 GN=CWC15 PE=1 SV=2 |
| Q6V1P9\|PCD23_HUMAN | 45 | 322234 | Protocadherin-23 OS=Homo sapiens OX=9606 GN=DCHS2 PE=2 SV=1 |
| Q8IWI9\|MGAP_HUMAN | 44.77 | 336160 | MAX gene-associated protein OS=Homo sapiens OX=9606 GN=MGA PE=1 SV=4 |
| Q8IY33\|MILK2_HUMAN | 44.51 | 97502 | MICAL-like protein 2 OS=Homo sapiens OX=9606 GN=MICALL2 PE=1 SV=1 |
| Q9BZQ8\|NIBA1_HUMAN | 43.96 | 103135 | Protein Niban 1 OS=Homo sapiens OX=9606 GN=NIBAN1 PE=1 SV=1 |
| P61326\|MGN_HUMAN | 43.92 | 17164 | Protein mago nashi homolog OS=Homo sapiens OX=9606 GN=MAGOH PE=1 SV=1 |
| Q96A72\|MGN2_HUMAN | 43.92 | 17276 | Protein mago nashi homolog 2 OS=Homo sapiens OX=9606 GN=MAGOHB PE=1 SV=1 |
| O95340\|PAPS2_HUMAN | 43.66 | 69501 | Bifunctional 3'-phosphoadenosine 5'-phosphosulfate synthase 2 OS=Homo sapiens OX=9606 GN=PAPSS2 PE=1 SV=2 |
| O00422\|SAP18_HUMAN | 43.35 | 17561 | Histone deacetylase complex subunit SAP18 OS=Homo sapiens OX=9606 GN=SAP18 PE=1 SV=1 |
| O60568\|PLOD3_HUMAN | 42.19 | 84785 | Multifunctional procollagen lysine hydroxylase and glycosyltransferase LH3 OS=Homo sapiens OX=9606 GN=PLOD3 PE=1 SV=1 |
| P51610\|HCFC1_HUMAN | 42.02 | 208730 | Host cell factor 1 OS=Homo sapiens OX=9606 GN=HCFC1 PE=1 SV=2 |
| P01023\|A2MG_HUMAN | 41.66 | 163290 | Alpha-2-macroglobulin OS=Homo sapiens OX=9606 GN=A2M PE=1 SV=3 |
| Q5VZ89\|DEN4C_HUMAN | 40.54 | 212709 | DENN domain-containing protein 4C OS=Homo sapiens OX=9606 GN=DENND4C PE=1 SV=3 |
| Q86XP3\|DDX42_HUMAN | 39.33 | 102975 | ATP-dependent RNA helicase DDX42 OS=Homo sapiens OX=9606 GN=DDX42 PE=1 SV=1 |
| P35249\|RFC4_HUMAN | 39.13 | 39682 | Replication factor C subunit 4 OS=Homo sapiens OX=9606 GN=RFC4 PE=1 SV=2 |
| Q08211\|DHX9_HUMAN | 38.87 | 140958 | ATP-dependent RNA helicase A OS=Homo sapiens OX=9606 GN=DHX9 PE=1 SV=4 |
| P50570\|DYN2_HUMAN | 38.57 | 98064 | Dynamin-2 OS=Homo sapiens OX=9606 GN=DNM2 PE=1 SV=2 |
| P35637\|FUS_HUMAN | 38.55 | 53426 | RNA-binding protein FUS OS=Homo sapiens OX=9606 GN=FUS PE=1 SV=1 |
| Q9Y6V0\|PCLO_HUMAN | 38.29 | 560707 | Protein piccolo OS=Homo sapiens OX=9606 GN=PCLO PE=1 SV=5 |
| P55145\|MANF_HUMAN | 38.27 | 20700 | Mesencephalic astrocyte-derived neurotrophic factor OS=Homo sapiens OX=9606 GN=MANF PE=1 SV=3 |
| P67775\|PP2AA_HUMAN | 37.69 | 35594 | Serine/threonine-protein phosphatase 2A catalytic subunit alpha isoform OS=Homo sapiens OX=9606 GN=PPP2CA PE=1 SV=1 |
| O14908\|GIPC1_HUMAN | 37.55 | 36049 | PDZ domain-containing protein GIPC1 OS=Homo sapiens OX=9606 GN=GIPC1 PE=1 SV=2 |
| P35030\|TRY3_HUMAN | 37.32 | 32529 | Trypsin-3 OS=Homo sapiens OX=9606 GN=PRSS3 PE=1 SV=2 |
| Q9Y2I7\|FYV1_HUMAN | 36.86 | 237134 | 1-phosphatidylinositol 3-phosphate 5-kinase OS=Homo sapiens OX=9606 GN=PIKFYVE PE=1 SV=3 |
| Q04637\|IF4G1_HUMAN | 36.78 | 175490 | Eukaryotic translation initiation factor 4 gamma 1 OS=Homo sapiens OX=9606 GN=EIF4G1 PE=1 SV=4 |
| Q6WKZ4\|RFIP1_HUMAN | 36.36 | 137167 | Rab11 family-interacting protein 1 OS=Homo sapiens OX=9606 GN=RAB11FIP1 PE=1 SV=3 |
| Q6ZVM7\|TM1L2_HUMAN | 36.04 | 55556 | TOM1-like protein 2 OS=Homo sapiens OX=9606 GN=TOM1L2 PE=1 SV=1 |
| Q6ZN44\|UNC5A_HUMAN | 35.62 | 92931 | Netrin receptor UNC5A OS=Homo sapiens OX=9606 GN=UNC5A PE=1 SV=3 |
| Q96QZ7\|MAGI1_HUMAN | 35.31 | 164580 | Membrane-associated guanylate kinase WW and PDZ domain-containing protein 1 OS=Homo sapiens OX=9606 GN=MAGI1 PE=1 SV=3 |
| P35998\|PRS7_HUMAN | 35.21 | 48634 | 26S proteasome regulatory subunit 7 OS=Homo sapiens OX=9606 GN=PSMC2 PE=1 SV=3 |
| Q02388\|CO7A1_HUMAN | 35.2 | 295216 | Collagen alpha-1(VII) chain OS=Homo sapiens OX=9606 GN=COL7A1 PE=1 SV=2 |
| A4UGR9\|XIRP2_HUMAN | 34.36 | 382302 | Xin actin-binding repeat-containing protein 2 OS=Homo sapiens OX=9606 GN=XIRP2 PE=1 SV=2 |
| Q5TBA9\|FRY_HUMAN | 33.33 | 338875 | Protein furry homolog OS=Homo sapiens OX=9606 GN=FRY PE=1 SV=1 |
| Q12789\|TF3C1_HUMAN | 33.27 | 238873 | General transcription factor 3C polypeptide 1 OS=Homo sapiens OX=9606 GN=GTF3C1 PE=1 SV=4 |
| O95347\|SMC2_HUMAN | 33.1 | 135656 | Structural maintenance of chromosomes protein 2 OS=Homo sapiens OX=9606 GN=SMC2 PE=1 SV=2 |
| Q9BZZ5\|API5_HUMAN | 32.98 | 59005 | Apoptosis inhibitor 5 OS=Homo sapiens OX=9606 GN=API5 PE=1 SV=3 |
| Q15477\|SKIV2_HUMAN | 32.97 | 137755 | Helicase SKI2W OS=Homo sapiens OX=9606 GN=SKIV2L PE=1 SV=3 |
| Q07864\|DPOE1_HUMAN | 32.83 | 261515 | DNA polymerase epsilon catalytic subunit A OS=Homo sapiens OX=9606 GN=POLE PE=1 SV=5 |
| Q9NS87\|KIF15_HUMAN | 32.81 | 160159 | Kinesin-like protein KIF15 OS=Homo sapiens OX=9606 GN=KIF15 PE=1 SV=1 |
| Q07283\|TRHY_HUMAN | 32.75 | 253922 | Trichohyalin OS=Homo sapiens OX=9606 GN=TCHH PE=1 SV=2 |
| P11137\|MTAP2_HUMAN | 32.7 | 199525 | Microtubule-associated protein 2 OS=Homo sapiens OX=9606 GN=MAP2 PE=1 SV=4 |
| Q9Y4A5\|TRRAP_HUMAN | 32.42 | 437603 | Transformation/transcription domain-associated protein OS=Homo sapiens OX=9606 GN=TRRAP PE=1 SV=3 |
| Q63HN8\|RN213_HUMAN | 32.32 | 591415 | E3 ubiquitin-protein ligase RNF213 OS=Homo sapiens OX=9606 GN=RNF213 PE=1 SV=3 |
| Q9H0B3\|IQCN_HUMAN | 32.11 | 127693 | IQ domain-containing protein N OS=Homo sapiens OX=9606 GN=IQCN PE=1 SV=1 |
| P42695\|CNDD3_HUMAN | 31.8 | 168890 | Condensin-2 complex subunit D3 OS=Homo sapiens OX=9606 GN=NCAPD3 PE=1 SV=2 |
| P06239\|LCK_HUMAN | 31.59 | 58001 | Tyrosine-protein kinase Lck OS=Homo sapiens OX=9606 GN=LCK PE=1 SV=6 |
| Q92618\|ZN516_HUMAN | 31.46 | 124289 | Zinc finger protein 516 OS=Homo sapiens OX=9606 GN=ZNF516 PE=1 SV=1 |
| P82987\|ATL3_HUMAN | 31.42 | 188691 | ADAMTS-like protein 3 OS=Homo sapiens OX=9606 GN=ADAMTSL3 PE=1 SV=4 |
| Q8IVF4\|DYH10_HUMAN | 31.32 | 514845 | Dynein heavy chain 10 axonemal OS=Homo sapiens OX=9606 GN=DNAH10 PE=1 SV=4 |
| Q9C0C2\|TB182_HUMAN | 31.23 | 181795 | 182 kDa tankyrase-1-binding protein OS=Homo sapiens OX=9606 GN=TNKS1BP1 PE=1 SV=4 |
| P20930\|FILA_HUMAN | 31.17 | 435180 | Filaggrin OS=Homo sapiens OX=9606 GN=FLG PE=1 SV=3 |
